# Unlocking NIR‐II Photoluminescence in 2D Copper Tetrasilicate Nanosheets through Flame Spray Synthesis

**DOI:** 10.1002/adma.202503159

**Published:** 2025-06-13

**Authors:** Robert Nißler, Quanyu Zhou, Björn Hill, Sabrina L.J. Thomä, Lukas R.H. Gerken, Aurelio Borzi, Kevin Roost, Benjamin Mächler, Xosé Luís Deán‐Ben, Antonia Neels, Sebastian Kruss, Daniel Razansky, Inge K. Herrmann

**Affiliations:** ^1^ Nanoparticle Systems Engineering Laboratory Institute of Energy and Process Engineering (IEPE) Department of Mechanical and Process Engineering (D‐MAVT) ETH Zurich Sonneggstrasse 3 Zurich 8092 Switzerland; ^2^ Particles‐Biology Interactions Department of Materials Meet Life Swiss Federal Laboratories for Materials Science and Technology (Empa) Lerchenfeldstrasse 5 St. Gallen 9014 Switzerland; ^3^ The Ingenuity Lab University Hospital Balgrist University of Zurich Forchstrasse 340 Zurich 8008 Switzerland; ^4^ Institute of Pharmacology and Toxicology Faculty of Medicine University of Zurich Winterthurerstrasse 190 Zurich 8057 Switzerland; ^5^ Institute for Biomedical Engineering Department of Information Technology and Electrical Engineering ETH Zurich Wolfgang‐Pauli‐Strasse 27 Zurich 8093 Switzerland; ^6^ Department of Chemistry Bochum University Universitätsstraße 150 44801 Bochum Germany; ^7^ Center for X‐ray Analytics Swiss Federal Laboratories for Materials Science and Technology (Empa) Ueberlandstrasse 143 Dübendorf 8600 Switzerland; ^8^ Fraunhofer Institute for Microelectronic Circuits and Systems 47057 Duisburg Germany

**Keywords:** 2D nanosheets, copper tetrasilicates, egyptian blue, NIR contrast agent, NIR fluorescence

## Abstract

Expanding fluorescence bioimaging into the second near‐infrared spectrum (NIR‐II, 1000–1700 nm) unlocks advanced possibilities for diagnostics and therapeutics, offering superior tissue penetration and resolution. 2D copper tetrasilicate (CTS) pigments (*M*CuSi_4_O_10_, *M* = Ca, Sr, Ba) are known for their brightness and stability, yet synthetic challenges have curbed their integration into bioimaging. Here, flame‐spray‐pyrolysis (FSP) is introduced as a versatile and scalable synthesis approach to produce ultra‐bright, metastable CTS nanosheets (NS) by annealing multi‐element metal oxide nanoparticles into 2D crystals through calcination or laser irradiation. Group‐II ion incorporation shifts emission into the NIR‐II range, with Ba_0.33_Sr_0.33_Ca_0.33_CuSi_4_O_10_ peaking at 1007 nm, while minor Mg‐doping induces a hypsochromic shift and extends fluorescence lifetimes. The engineered CTS achieves quantum yields of up to 34%, supporting NS high‐frame‐rate imaging (> 200 fps). These unique properties enable CTS‐NS to serve as powerful contrast agents for super‐resolution NIR bioimaging, demonstrated in vivo through transcranial microcirculation mapping and macrophage tracking in mice using diffuse optical localization imaging (DOLI). This pioneering synthesis strategy unlocks wavelength‐tunable NS for advanced NIR‐II bioimaging applications.

## Graphical abstract

A flame‐spray‐pyrolysis method is presented to synthesize ultra‐bright CTS nanosheets with tunable NIR‐II emission. Achieving quantum yields up to 34%, these materials support high‐speed imaging and enable super‐resolution in vivo applications such as transcranial microcirculation mapping and macrophage tracking.

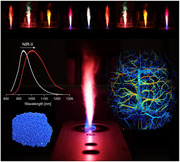

## 1 Introduction

Pushing the boundaries of fluorescence bioimaging into the second near‐infrared spectrum (NIR‐II, 1000–1700 nm) is pivotal for enhancing its diagnostic and therapeutic capabilities in biomedical research. This spectrum holds immense potential for deep‐tissue imaging due to reduced autofluorescence and light scattering.^[^ ^1^, ^2^ ^]^ While conventional agents have demonstrated efficacy in the first near‐infrared (NIR‐I, 700–950 nm) spectral window, they often perform inadequately in the NIR‐II spectrum, underscoring the need for novel contrast agents designed specifically for this range. Current fluorophores used in this bio‐transparency window include organic dyes, such as indocyanine green (ICG),^[^ ^3^ ^]^ as well as inorganic structures like quantum dots (QD),^[^ ^4^, ^6^ ^]^ single‐walled carbon nanotubes (SWCNTs),^[^ ^7^ ^]^ and rare‐earth element‐doped nanoparticles.^[^ ^8^, ^9^ ^]^ Each aforementioned material offers distinct benefits, yet the ideal material would be characterized by photostability, a significant Stokes shift, and a high photoluminescence quantum yield (PL‐QY) within the NIR‐II window. Additionally, the ideal material should ensure biocompatibility, possess advantageous colloidal properties, and be amenable to surface functionalization. Such attributes would greatly enhance the imaging performance of existing bioimaging tools by improving spatial resolution and penetration depth, paving the way for functional and molecular imaging. This, in turn, would expand the applicability of optical bioimaging in disease diagnostics, treatment evaluations, and image‐guided surgeries.^[^ ^10^ ^]^

A particular class of nanostructured pigments, renowned for centuries but recently experiencing a resurgence in interest, are 2D copper tetrasilicates (CTS) with the general structure *M*CuSi_4_O_10_, where *M* denotes the alkaline earth metals Ca, Sr, or Ba.^[^ ^11^, ^13^ ^]^ The most renowned silicate from the gillespite‐group is CaCuSi_4_O_10_, commonly referred to as Egyptian blue. Its synthesis traces back to Ancient Egypt (circa 2500 BC), cementing its status as the earliest known pigment crafted by humanity. Its structural counterpart, BaCuSi_4_O_10_ (known as Han Blue), emerged in ancient Chinese pottery during the Han dynasties (circa 220 BC – 220 AD).^[^ ^14^ ^]^ Intriguingly, the vivid hues of these materials have withstood the toll of time, retaining their unique luminescent attributes, particularly a potent fluorescence emission in the NIR‐I at ≈910 nm.^[^ ^2^, ^15^, ^16^ ^]^ CTSs embodying a multitude of the aforementioned desired characteristics, emerge as promising candidates for bioimaging and sensing applications.^[^ ^17^ ^]^ Yet their full potential, particularly as NIR‐II emissive agents, remains relatively unexplored but holds immense promise. These blue pigments can be synthesized by various approaches,^[^ ^17^ ^]^ creating bulk material with different levels of purities and crystal sizes. Most commonly, salt‐flux or solid‐state synthesis is used, based on, e.g., the ancient process whereas respective minerals are finely grinded and heated to 900 °C over several hours, while sintering to the desired crystal phase takes place at the grain boundaries.^[^ ^14^ ^]^ Additionally, methods like hydrothermal synthesis can produce mesoporous microspheres of narrow size distribution, however without the possibility for straightforward compositional engineering and being limited in production scale.^[^ ^18^ ^]^ Previous studies have unveiled that the laminar architecture of the CTSs can be exfoliated into 2D nanosheets (NS) as thin as a single crystal lattice.^[^ ^11^, ^13^ ^]^ Remarkably, these NS resist photobleaching and exhibit a polarization‐neutral emission, setting them apart as a promising NIR reference material for sensing applications.^[^ ^19^, ^20^ ^]^ To obtain NS, multiple approaches are known, combining breaking down the crystal structure and exfoliating it via ball milling, sonication, prolonged stirring in solvents or a combination of all of them.^[^ ^17^, ^21^, ^22^ ^]^ However, in recent studies it became apparent that intensified mechanical stress due to exfoliating and milling these materials down to the nanoscale substantially degraded their fluorescence emission properties.^[^ ^23^ ^]^ These findings significantly constrain potential applications and underscore the necessity for a scalable and tunable synthesis route that can produce highly fluorescent nanomaterials for use as high‐performance bioimaging agents.

In this work, we report the synthesis of NIR‐II emissive CTS‐NS, offering new possibilities for bioimaging techniques. To enable the NIR‐II emission of CTSs, we demonstrate that bottom‐up NS synthesis can be accomplished by annealing multi‐doped nanoparticles, initially created through flame‐spray synthesis, into 2D nanocrystals. Stoichiometric metal–organic precursor solutions are injected into a CH_4_/O_2_ flame, where controlled combustion of the organic solvent and subsequent nucleation of metal oxides form nanoparticles with a homogeneous elemental distribution. Annealing these FSP‐derived particles can be realized through calcination or laser irradiation and enables the controlled incorporation of group‐II cations into the CTS crystal structure, yielding NIR‐emissive engineered materials that can be exfoliated into 2D‐NS. These NS are then harnessed to assess their potential for real‐time, deep‐tissue, super‐resolution bioimaging, enabling the mapping of the transcranial cortical microcirculation in mice using diffuse optical localization imaging (DOLI) and tracking individual macrophages across cerebral regions.

## 2 Results

### 2.1 Flame‐Spray Pyrolysis‐Based Synthesis of Group‐II Cation‐Doped Copper Silicates

**Figure 1:**
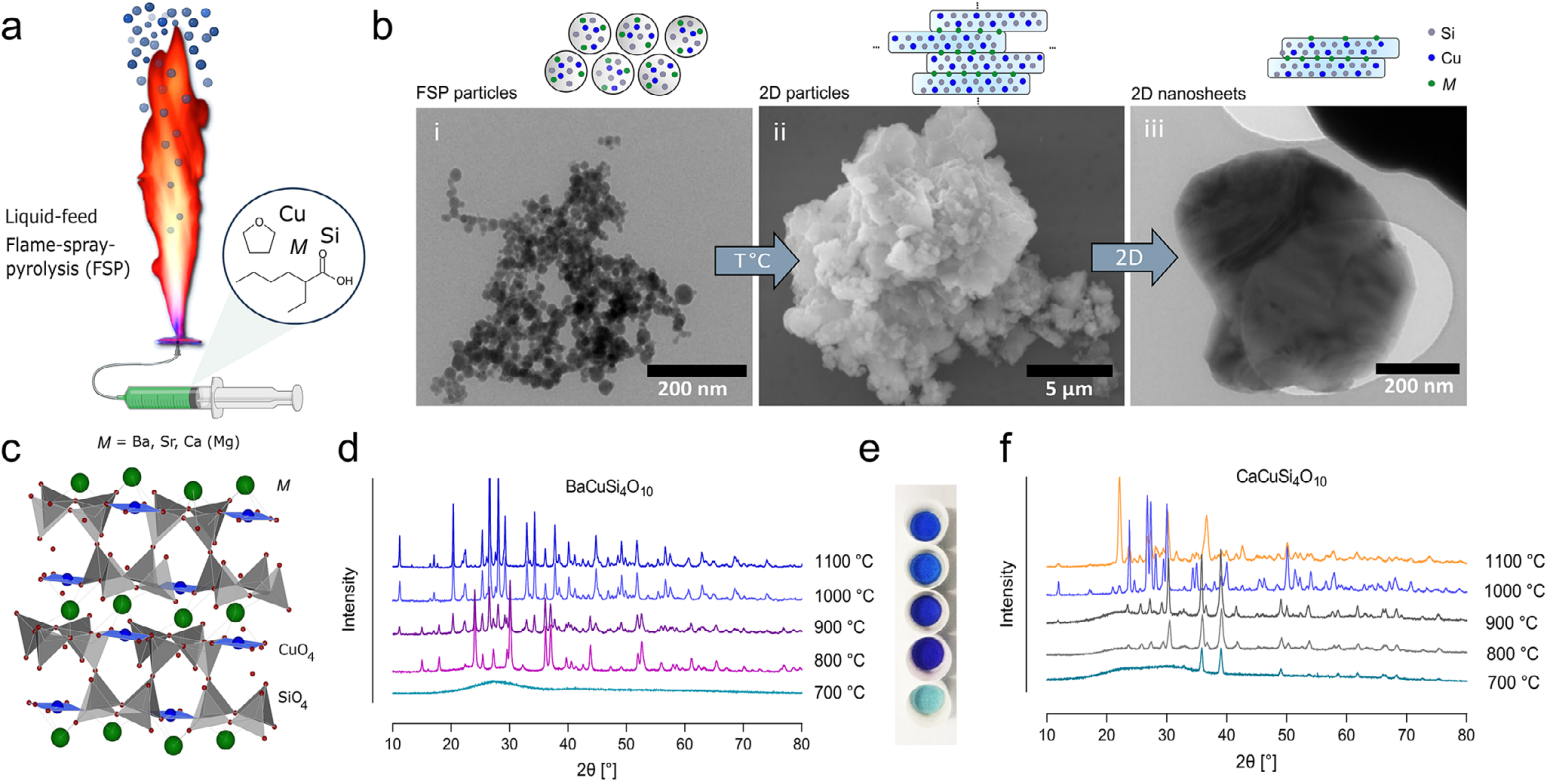
Flame‐spray‐pyrolysis of 2D copper tetrasilicates *M*CuSi_4_O_10_. a) Schematic representation of the synthesis process. Metal–organic precursor solution in tetrahydrofuran (THF) and 2‐ethylhexanoic acid (2‐EHA) is fed into a CH_4_/O_2_ flame, whereas emerging NP are collected on an above‐mounted filter. This synthesis procedure enables compositional engineering of the resulting CTS nanosheets (NS). b) Electron microscopic visualization of the NS synthesis with a schematic representation. 1) TEM image of the ≈30 nm large, spherical as‐prepared FSP NPs with homogeneous elemental distribution. 2) Annealing of as‐prepared FSP particles leads to the formation of 2D materials with the desired crystal structure (SEM image). 3) 2D material can be dispersed and exfoliated down to the nanosheet regime (TEM image). c) Visualization of the tetragonal phase (*P4/n c c*; #130) of *M*CuSi_4_O_10_. Alkaline earth metals (*M*) link monolayers of silica‐tetrahedra with square planar coordinated copper ions. d) XRD patterns of NPs with the nominal chemical composition of BaCuSi_4_O_10_, sintered at different temperatures. Picture of crucibles containing these NP in e). Annealing of as‐prepared FSP particles at 1000 or 1100 °C for 10 min leads to the near‐pure formation of the desired tetragonal crystal phase. f) XRD pattern of annealed NPs forming CaCuSi_4_O_10_ at 1000 °C. The colors of each XRD profile visualize the appearance of the nano‐powder. Likewise, FSP‐synthesis can also be used to produce SrCuSi_4_O_10_ (Figure S2, Supporting Information).

Historical synthesis approaches rely on the mixing and sintering of mineral phases containing elements, leading to phase defects at the grain boundaries. To address this limitation, we employ a highly robust and industrially viable method known as flame‐spray‐pyrolysis (FSP) to obtain metal‐oxide particles.^[^ ^24^ ^]^ In this process, metal–organic precursors with the desired elemental ratio are dissolved in organic solvents and injected into a CH_4_/O_2_ flame. Temperatures within the flame can reach > 3300 K whereas the spray exhibits velocities of ≈150 m s^−1^, causing the organic solvent to combust and leading the metal atoms to nucleate and form respective oxides.^[^ ^25^, ^26^ ^]^ The emerging nanoparticles are then collected on an above‐mounted filter (**Figure** **1**a). However, these FSP‐born silicate particles are initially amorphous and ≈30 nm in size, as observed via TEM analysis (Figure 1b,*i*), and exhibit a homogeneous distribution of the precursor elements Cu, Si, O, and *M*, where *M* = Ca, Sr, or Ba, as shown for multi‐element doped particles later in the manuscript. Annealing the nanoparticles at a specific temperature (Figure 1b,*ii*) results in bulk CTS powder with the desired 2D crystal structure, which can be further exfoliated to the single/few atomic layer NS regime (Figure 1b,*iii*). During this process, the crystal phase can be precisely tuned through screening of different annealing temperatures, facilitated by thermogravimetric analysis (TGA), until the tetragonal *P 4/n c c* space group is observed via X‐ray diffraction (XRD) analysis (Figure 1c). For example, as seen in the Ba‐containing CTS variation (Han blue, Figure 1d,e), the powder with primary FSP particles changes color from green‐cyan (FSP material) to purple (800 °C) to dark blue (1000–1100 °C), consistent with corresponding XRD analysis identifying the formation of tetragonal *I4_1_ /acd* space group at 800 °C, followed by a miscellaneous phase at 900 °C, and finally the desired *P 4/n c c* phase at 1000–1100 °C. To obtain other *M*CuSi_4_O_10_ variations, only the exchange of the respective alkaline earth metal solution during the preparation of the FSP precursor solution is needed. Annealing similar nanoparticles with *M* = Ca results in the formation of CaCuSi_4_O_10_ (Cuprorivaite, Egyptian blue) at 1000 °C (Figure 1f) within just a few minutes (Figure S1, Supporting Information), while the bulk material exhibits a light blue color. This modular synthesis approach is therefore capable of yielding all known CTS materials, including SrCuSi_4_O_10_ (Strontium Blue), solely by modifying the elemental composition and ratio via the FSP process (Figure S2, Supporting Information).

The surface area of the nanoparticles was found to decrease during the annealing process from ≈95–120 g m^−2^ of the FSP material to 3–13 g m^−2^ of the CTS phase, as measured by Brunauer–Emmett–Teller (BET) analysis (Table S1, Supporting Information). Rietveld refinement confirms that NS variations are yielded with high purity, containing > 98% of the desired crystal phase (Figure S3, Supporting Information) with similar lattice parameters as previously reported.^[^ ^27^ ^]^ It should be noted that attempts to directly synthesize CTS material using the FSP process were unsuccessful, even after encasing the flame aperture with a glass mantle to increase temperature and prolong circulation time above the flame.

### 2.2 Synthesis of Assorted Bi‐Doped Group‐II Cation Copper Tetrasilicates

**Figure 2:**
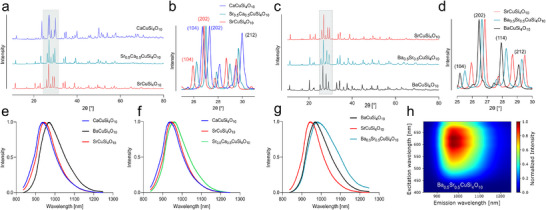
FSP‐based syntheses of mixed Ba–Sr and Sr–Ca copper tetrasilicates. a) XRD pattern of SrCuSi_4_O_10_ and CaCuSi_4_O_10_ and its mixed form Sr_0.5_Ca_0.5_CuSi_4_O_10_ with zoom in b) into the region between 25 and 31 °2θ highlighting the shifted (202) reflex of the mixed material. c) XRD pattern of BaCuSi_4_O_10_ and SrCuSi_4_O_10_ and its stoichiometrically exchanged form Ba_0.5_Sr_0.5_CuSi_4_O_10_ with zoom in d) into the region between 25 and 30 °2θ highlighting the similarly shifted reflexes. e) PL emission spectra of FSP‐synthesized mono‐alkaline earth metal CTS. f) PL emission spectra of Sr_0.5_Ca_0.5_CuSi_4_O_10_. g) PL emission spectra of Ba_0.5_Sr_0.5_CuSi_4_O_10_. h) 2D excitation‐emission PL map for Ba_0.5_Sr_0.5_CuSi_4_O_10_.

Further leveraging the modular and universal FSP synthesis approach, we then synthesized mixed alkaline earth metal CTS. By solely exchanging the respective fraction of alkaline earth metal precursor solution, an equimolar mixture of Ca and Sr led to the synthesis of Sr_0.5_Ca_0.5_CuSi_4_O_10_ (**Figure** **2**a; Figure S4, Supporting Information). The XRD reflexes (Figure 2b) of the mixed phase lie in between both single phases resulting in shifted *P 4/n c c* lattice parameters of *a* = 7.33 Å and *c* = 15.39 Å. Analogous experiments with an equimolar combination of Ba and Sr precursors lead to the formation of Ba_0.5_Sr_0.5_CuSi_4_O_10_ as depicted in Figure 2c. Again, the incorporation of smaller ions (Sr) into the BaCuSi_4_O_10_ crystal lattice resulted in a shift of the XRD reflexes toward greater 2θ values (Figure 2d). The lattice parameters of the Ba_0.5_Sr_0.5_CuSi_4_O_10_ phase are of *a* = 7.39 Å and *c* = 15.89 Å. Rietveld refinement furthermore confirmed the synthesis of high‐purity doped NS material (Figure S5, Supporting Information). NIR‐fluorescence spectroscopic characterization of the resulting material revealed the characteristic emission profile of the CTS with *M*CuSi_4_O_10_ with *M * =  Ca (λ_max_ = 936 nm), Sr (λ_max_ = 946 nm), Ba (λ_max_ = 968 nm) (Figure 2e). Comparing their emission to the Sr–Ca intermixed CTS material (Figure 2f), shows a slightly red‐shifted emission for the formed Sr_0.5_Ca_0.5_CuSi_4_O_10_ with λ_max_ = 957 nm.

The equimolar combination of Ba–Sr, however, showed a significant bathochromic shift with λ_max_ = 981 nm (Figure 2g), accompanied by a general broadening of the NIR‐emission of Ba_0.5_Sr_0.5_CuSi_4_O_10_, as seen in the 2D excitation‐emission PL map in Figure 2h. The PL emission maxima, as well as the UV–vis–NIR absorbance spectra (Figure S6, Supporting Information) of the known CTS are in accordance with previous reports.^[^ ^17^ ^]^ The spectral redshift of Sr_0.5_Ca_0.5_CuSi_4_O_10_ is in agreement with a recent report, identifying an equimolar ratio of Sr to Ca (1:1) as the most optimal ratio to achieve an enhanced bathochromic emission shift.^[^ ^28^ ^]^ To our knowledge, ours is the first report of the significantly bathochromic‐shifted emission properties of Ba_0.5_Sr_0.5_CuSi_4_O_10_. The UV–vis–NIR absorbance spectra of both bi‐doped group‐II cation CTS showed a slight broadening of the B_1g_‐E_g_ transition, as well as a minor hyperchromic shift in B_1g_‐A_1g_ transition (Figure S7, Supporting Information).

### 2.3 FSP‐Synthesis Gives Access to Meta‐Stable Ba–Ca Copper Tetrasilicates

Given that the synthesis of Sr_0.5_Ca_0.5_CuSi_4_O_10_ and Ba_0.5_Sr_0.5_CuSi_4_O_10_ is reported,^[^ ^28^, ^33^ ^]^ albeit using poorly scalable methods, we aimed to evaluate whether the FSP synthesis approach could yield alkaline earth metal combinations explicitly described as “forbidden” or inaccessible through established methods, such as the combination of Ba and Ca cations within the CTS lattice.^[^ ^14^, ^33^ ^]^ We hypothesized that the optimal elemental distribution within the FSP‐derived primary particles could facilitate a homogeneous alkaline earth metal distribution within the annealed CTS‐NSs.

**Figure 3:**
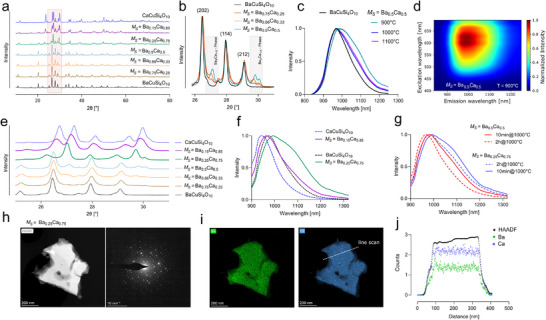
FSP‐based syntheses enable intermixed Ba–Ca copper tetrasilicates. a) XRD pattern of BaCuSi_4_O_10_ and CaCuSi_4_O_10_ and their mixed forms, expressed as the synthesis ratio of the alkaline earth metals (*M_S_*) with zoom in b) visualizing the co‐existence of the BaCuSi_4_O_10_‐phase and an intermixed Ba_x_Ca_1‐x_CuSi_4_O_10_‐phase until *M_S_* = Ba_0.5_Ca_0.5_. c) PL emission spectra of *M_S_* = Ba_0.5_Ca_0.5_ annealed at different temperatures. d) 2D excitation‐emission PL map for *M_S_* = Ba_0.5_Ca_0.5_ annealed at 900 °C, as it contains ≈60% Ba_x_Ca_1‐x_CuSi_4_O_10_ evaluated by Rietveld refinement. e) XRD pattern of BaCuSi_4_O_10_ and CaCuSi_4_O_10_ and their mixed forms, between 25 and 31° 2θ highlighting the existence of a shifted, single phase at *M_S_* = Ba_0.15_Ca_0.85_ and *M_S_* = Ba_0.25_Ca_0.75_. f) PL emission spectra of CTSs, originated from various Ba–Ca synthesis ratios. g) Influence of annealing time on the PL emission spectra of *M_S_* = Ba_0.5_Ca_0.5_ and *M_S_* = Ba_0.25_Ca_0.75_ indicating the existence of a meta‐stable Ba_x_Ca_1‐x_CuSi_4_O_10_‐phase, exhibiting a bathochromic shift in the emission maxima. h) STEM analysis of a single Ba_0.25_Ca_0.75_CuSi_4_O_10_ NS shown as HAADF image and corresponding electron diffraction (ED) pattern. i) EDX‐based elemental mapping of Ba and Ca. j) Line‐profile (50‐pixel width) showing a homogeneous distribution of Ba and Ca within the NS.

The combination of such mixed forms could be tested straightforwardly by altering the Ba and Ca precursor ratios, whereby the XRD pattern of the resulting materials is presented in **Figure** **3**a given as the synthesis ratio of the alkaline earth metals (*M_S_*). Up to an *M_S_* = Ba_0.75_Ca_0.25_ and *M_S_* = Ba_0.5_Ca_0.5_ a predominant, non‐shifted BaCuSi_4_O_10_ phase was observed, with a minor reflex of a shifted, Ba–Ca mixed CTS phase as highlighted in Figure 3a,b. We correlate the formation of such a Ba_x_Ca_1‐x_CuSi_4_O_10_‐phase with a significantly impacted PL emission, as samples exhibited a bathochromic emission shift, highlighted for *M_S_* = Ba_0.5_Ca_0.5_ in Figure 3c. However, these shifted emission features, as measured for a successful Ba_x_Ca_1‐x_CuSi_4_O_10_‐phase formation seemed to be annealing temperature dependent. *M_S_* = Ba_0.5_Ca_0.5_ annealed at 900 °C exhibited an emission maximum of 984 nm with an emission tail spanning far into the NIR‐II spectral window (Figure 3d; Figure S8, Supporting Information), containing ≈60% of a Ba–Ca mixed CTS phase as evaluated by Rietveld refinement (Figure S9, Supporting Information). Annealing at 1000 or 1100 °C showed only a slight emission shift compared to BaCuSi_4_O_10_, containing only ≈31% of Ba_x_Ca_1‐x_CuSi_4_O_10_ (Figure S9, Supporting Information). Most interestingly, a synthesis ratio of *M_S_* = Ba_0.15_Ca_0.85_ and *M_S_* = Ba_0.25_Ca_0.75_ resulted in the near‐exclusive formation of a Ba–Ca mixed copper tetrasilicate phase, possessing intermediate lattice parameters (Figure 3e). Rietveld refinement revealed that *M_S_* = Ba_0.15_Ca_0.85_ annealed for 10 min at 1000 °C contains 87% of a Ba_x_Ca_1‐x_CuSi_4_O_10_ phase with lattice parameters of *a* = 7.31 Å and *c* = 15.18 Å. *M_S_* = Ba_0.25_Ca_0.75_ annealed for 10 min at 1000 °C contained 93% of a Ba_x_Ca_1‐x_CuSi_4_O_10_ phase with lattice parameters of *a* = 7.32 Å and *c* = 15.38 Å. However, both samples surprisingly contained a second inter‐mixed Ba–Ca phase, representing 13% and respectively 7% of the material with significantly larger lattice parameters (Figure S11, Supporting Information). Based on these lattice parameters, it is expected that the observed Ba–Ca mixed CTS phases vary in their Ba–Ca ratios. However, the exact compositions could not be determined through Rietveld refinement due to significant overlap in the reflections of the two different tetragonal *P 4/n c c* phases, particularly in the 25–32° 2θ region. Analyzing the PL emissions of the materials revealed a strong bathochromic shift for *M_S_*  = Ba_0.25_Ca_0.75_ with an λ_max_ = 993 nm and a significant broadening toward the NIR‐II (Figure 3f; Figure S12, Supporting Information). *M_S_* = Ba_0.15_Ca_0.85_ on the other hand, showed a drastic shift with λ_max_ = 963 nm compared to CaCuSi_4_O_10_ (λ_max_ = 936 nm) indicating, that exchanging 15% of the alkaline earth metals with Ba only slightly alters the crystal lattice but strongly impacts the fluorescence emission. The obtained Ba–Ca intermixed phase, however, seemed to be meta‐stable, as extended annealing times of 2–24 h lead to an enhanced segregation into minorly doped Ba‐ and Ca‐phases (Figures S13 and S10, Supporting Information), accompanied by a decreased bathochromic shift (Figure 3g). To further verify the creation of a single Ba_x_Ca_1‐x_CuSi_4_O_10_‐phase on a nanoscale level, we performed scanning transmission electron microscopy (STEM) analysis of single *M_S  _* = Ba_0.25_Ca_0.75 _NS.

The resulting HAADF image and corresponding electron diffraction (ED) pattern are shown in Figure 3h, suggesting a highly crystalline material with a typical ED pattern for materials of the *P 4/n c c* space group. Further EDX‐based elemental mapping showed a strong co‐localization between Ba and Ca (Figure 3i; full elemental mapping in Figure S14, Supporting Information) whereas a line profile horizontally to the NS revealed a homogeneous distribution of both alkaline earth metals within the NS (Figure 3j). Moreover, EDX analysis yielded the alkaline earth metals ratio (*M_EDX_*) between Ba and Ca of *M_EDX_* = Ba_0.78_Ca_0.22_, overall, very close to the FSP‐based precursor ratio. To our knowledge, this is the first time that intermixed copper tetrasilicates have been synthesized, incorporating substantial amounts of both Ba and Ca.^[^ ^34^ ^]^ Likely due to the differences in atomic (metallic) radii with Ba = 2.24 Å and Ca = 1.97 Å, the incorporation is unfavored and allows no continuous mixing under all tested synthesis ratios (*M_S_*). As a consequence, in excess Ba‐conditions (*M_S_* = Ba_0.75_Ca_0.25_) a predominant BaCuSi_4_O_10_ phase will form with shifted emission features, but no significant shift in XRD pattern, whereas under excess Ca‐conditions (*M_S_* = Ba_0.25_Ca_0.75_) nearly a single, intermixed phase is observed with significantly shifted emission properties and XRD reflexes. However, as the PL of *M_S_* = Ba_0.75_Ca_0.25_ shows a red‐shifted emission maxima and a broadening toward the NIR‐II (see Figure S12, Supporting Information), but only a marginal Ba_x_Ca_1‐x_CuSi_4_O_10_‐phase according to the XRD pattern, we attribute the shifted emission to slight doping of the main BaCuSi_4_O_10_ phase, without significantly altering the XRD reflex positions. This implies that the photoluminescence behavior of the NS might be more sensitive to doping with other alkaline earth metals compared to the occurrence of an overall shift in the crystal phase and XRD pattern, as doping levels might differ to induce significant changes in both analytical measurements. As a consequence thereof, we introduce the nomenclature of doped CTS composites in order of heavier to lighter alkaline earth metals, in cases where an altered crystal phase could be confirmed by XRD. Other synthesis ratios (*M_S_*) that resulted in insignificant shifts in XRD but caused significant modulation of the PL emission properties, compared to the native phase, are referred to as *M*‐doped CTS. Previous studies already indicated that, e.g., minor doping of CaCuSi_4_O_10_ could induce a shift in emission spectra, however without significant altering of lattice parameters.^[^ ^34^ ^]^ This leads us to the conclusion that at higher Ba–Ca ratios, a Ca‐doped BaCuSi_4_O_10_ phase is generated, whereas equimolar rations of Ba–Ca leads to the co‐existence of a likely Ca‐doped BaCuSi_4_O_10_ phase and a mixed Ba_x_Ca_1‐x_CuSi_4_O_10_ phase. Finally, under excess Ca to Ba conditions, a near‐exclusive intermixed Ba_x_Ca_1‐x_CuSi_4_O_10_ phase is detected. In contrast to the mixed phases of Sr_0.5_Ca_0.5_CuSi_4_O_10_ and Ba_0.5_Sr_0.5_CuSi_4_O_10_, the Ba‐Ca phases such as Ba_0.25_Ca_0.75_CuSi_4_O_10_ were found to be meta‐stable, as prolonged annealing times would lead to the segregation of the mixed phases. This implies that the intermixed Ba‐Ca sample could not reach its equilibrium, shining light on the synthesis process of the doped CTS‐NS. As starting from homogenously distributed elements within the FSP‐derived particles, the thermal‐induced rearrangement to the 2D crystal lattice seems to occur faster than the segregation into stable, mono‐alkaline earth metal CTS.

### 2.4 Multielement Doping of Copper Tetrasilicates Tailors the PL Emission

**Figure 4:**
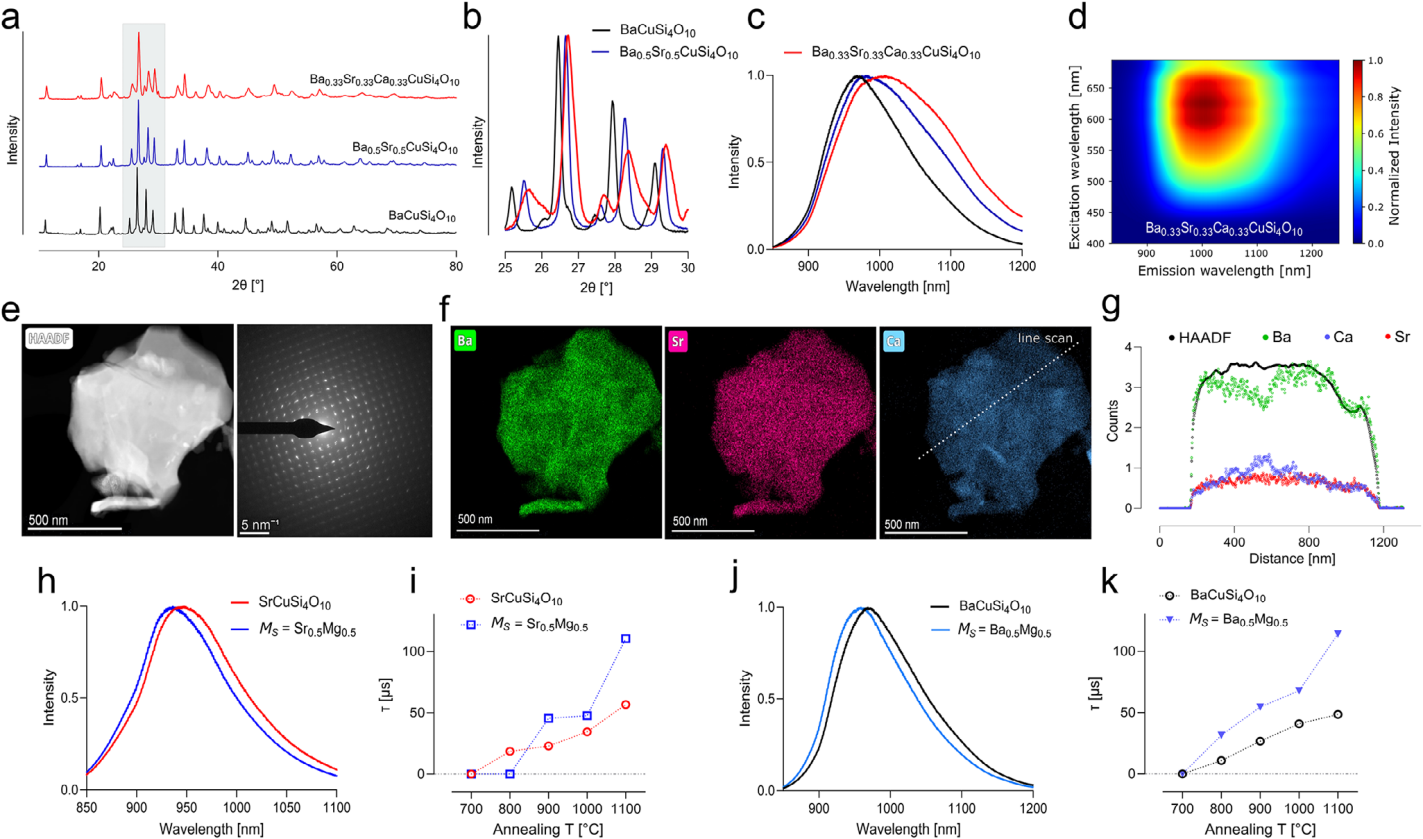
Multielement doping of copper tetrasilicates. a) XRD pattern of BaCuSi_4_O_10_ compared to the multi‐element doped version Ba_0.33_Sr_0.33_Ca_0.33_CuSi_4_O_10_ with zoom in b) into the region between 25 and 30° 2θ visualizing the shifted and broadened reflexes. c) PL emission spectra of Ba_0.33_Sr_0.33_Ca_0.33_CuSi_4_O_10_ show a significant bathochromic shift compared to Ba_0.5_Sr_0.5_CuSi_4_O_10_. d) 2D excitation‐emission PL map of Ba_0.33_Sr_0.33_Ca_0.33_CuSi_4_O_10_. e) STEM analysis of a single Ba_0.33_Sr_0.33_Ca_0.33_CuSi_4_O_10_ NS shown as HAADF image and corresponding electron diffraction (ED) pattern. f) EDX‐based elemental mapping of Ba, Sr, and Ca. g) Line‐profile (50‐pixel width) showing a near homogeneous distribution of Ba, Sr and Ca within the NS. h) PL emission spectra of Mg‐doped‐SrCuSi_4_O_10_, showing a blue‐shifted emission compared to the non‐Mg‐doped material. i) Fluorescence lifetimes of CTSs presented in (h) showing increased lifetime values for Mg‐doped‐SrCuSi_4_O_10_ (mean ± SD). j) PL emission spectra of the Mg‐doped‐BaCuSi_4_O_10_, resulting from *M_S _* = Ba_0.5_Mg_0.5_ showing a blue‐shifted emission maxima compared to bare BaCuSi_4_O_10._ k) Fluorescence lifetime modulation through annealing temperature variation indicating enhanced lifetimes for Mg‐doped‐BaCuSi_4_O_10_ (mean ± SD).

Building upon the successful combination of Ba–Ca, we investigated the multi‐element‐doping of NS and the synthesis with equimolar ratios of Ba, Sr, and Ca precursors, which showed again homogeneous distribution within the FSP‐particles (Figure S15, Supporting Information). Annealing at 1000 °C suggests the formation of Ba_0.33_Sr_0.33_Ca_0.33_CuSi_4_O_10_. The corresponding XRD pattern depicted in **Figure** **4**a,b reveals a slight shift in the peak positions of the doped tetragonal *P4/n c c* phase, along with substantial peak broadening. Notably, Rietveld refinement alone could not quantify the composition of the multielement‐doped material, as all three cations occupy the same position. Similar agreement values would be obtained even when the refinement was performed with only Ba and Sr sharing this position. The fluorescence emission of the resulting nanomaterial shows a broadened, bathochromic shift, compared to Ba_0.5_Sr_0.5_CuSi_4_O_10_ (Figure 4c), with λ_max_ = 1007 nm and Full Width at Half Maximum (FWHM) of 197 nm. This broad emission spans far into the NIR‐II spectral window, as seen in the 2D excitation‐emission PL map in Figure 4d.

The Ba_0.33_Sr_0.33_Ca_0.33_CuSi_4_O_10_ material was found not to undergo significant changes with prolonged annealing times (Figure S16, Supporting Information), indicating a stable formation of multi‐element doped NS. Complementary STEM analysis on a single Ba_0.33_Sr_0.33_Ca_0.33_CuSi_4_O_10_ NS was performed to access the structure and elemental distribution with nanoscale resolution. Figure 4e shows the HAADF image and corresponding electron diffraction pattern of the NS, accentuating the highly crystalline nature of the doped material with a typical ED pattern for the respective CTS material class. EDX‐based elemental mapping indicates a strong co‐localization between Ba, Sr, and Ca (Figure 4f), whereas the corresponding line scan in Figure 4g indicates a near‐homogeneous distribution of the three alkaline earth metals. However, analysis of multiple Ba_0.33_Sr_0.33_Ca_0.33_CuSi_4_O_10_ NSs indicated a general trend of homogeneous distribution of Sr within the NS lattice, whereas the local concentration of Ba and Ca reciprocally could slightly vary, however without segregating into separate phases (Figure S17, Supporting Information). Ba_0.33_Sr_0.33_Ca_0.33_CuSi_4_O_10_ annealed for 24 h at 1000 °C exhibited a minor Ca‐containing phase, resulting in a slight underrepresentation of Ca in the overall EDX analysis of the doped NS. The alkaline earth metals ratio quantified by EDX was *M_EDX_* = Ba_0.36_Sr_0.39_Ca_0.25_, closely matching the FSP‐based precursor ratio. The reproducibility of the synthesis process was assessed by analyzing the EDX‐based elemental ratios from three independent Ba_0.33_Sr_0.33_Ca_0.33_CuSi_4_O_10_ synthesis batches, annealing the as‐produced FSP particles for 2 h at 1000 °C (Figure S18a, Supporting Information). The shorter calcination time reduced the presence of a segregated Ca‐containing phase. The EDX‐based alkaline earth metals ratio from all three replicates was *M_EDX_* = Ba_0.35±0.01_Sr_0.31±0.01_Ca_0.34±0.02_ (mean ± SD, N = 3). With an overall variance of ≈±5% from the desired ratio, the FSP synthesis approach demonstrates its robustness in achieving precise alkaline earth metal ratios, which are essential for consistent nanosheet engineering and production. It should be noted that direct observation of doping sites using advanced electron microscopy techniques, such as aberration‐corrected TEM, was not possible due to the beam sensitivity of the NS samples, which led to significant degradation within minutes at beam energies ≥  200 kV (Figure S18b, Supporting Information).

To complete our efforts to dope the copper tetrasilicates with group‐II cations, we aimed for the introduction of Mg into the NS crystal lattice. Again, solely by exchanging equimolar ratios of the respective alkaline earth metal from the FSP precursors solution, amorphous FSP‐NPs were generated that obtained a homogeneous distribution of all admixed elements (Figure S19, Supporting Information). Annealing of *M_S_*  = Sr_0.5_Mg_0.5_ particles formed CTS material, processing a light blue color, however, further XRD analysis could not identify significant shifts of the *P4/n c c* phase toward larger reflex positions, as they would be expected for incorporation of smaller Mg atoms (1.6 Å) into the crystal lattice (Figure S20, Supporting Information).

In contrast, fluorescence spectroscopic analysis revealed 12 nm blue‐shifted emission maxima for *M_S _*  = Sr_0.5_Mg_0.5_ with λ_max_ = 935 nm, compared to the non‐doped emission of SrCuSi_4_O_10_ (λ_max_ = 947 nm). Simultaneously, the obtained NS material exhibited increased fluorescence lifetime, reaching a maximum when annealed at 1100 °C for 10 min of τ = 110 µs (Figure 4i) and of τ = 153 µs, when annealed for 3 h. Similar trends were observed for *M_S_* = Ba_0.5_Mg_0.5_ NS, possessing 10 nm blue‐shifted emission maxima of λ_max_ = 958 nm compared to BaCuSi_4_O_10_ (Figure 4j). The fluorescence lifetime of the obtained NS increased to τ = 114 µs, when annealed at 1100 °C for 10 min (Figure 4k) and to τ = 128 µs after annealing at 1000 °C for 24 h (Figure S20, Supporting Information). Complementary STEM analysis showed a strong segregation of the Mg‐containing phases, however with traces of Mg (<0.5% atomic fraction) within the non‐segregated CTSs. These findings led us to the conclusion, that the incorporation of Mg into the crystal phase is highly unfavored and happens only in minor quantities, which does not significantly impact the overall lattice parameters as evaluated by Rietveld refinement (Figures S21 and S22, Supporting Information). However, we attribute the altered photoluminescence properties of the materials, such as blue‐shifted emission and increased lifetimes, to an overall slight Mg‐doping of the copper tetrasilicates based on EDX. Further particles were synthesized with *M_S_* = Ca_0.9_Mg_0.1_, *M_S_* = Sr_0.33_Ca_0.33_Mg_0.33_, *M_S_* = Ba_0.33_Sr_0.33_Mg_0.33,_ and *M_S_* = Ba_0.25_Sr_0.25_Ca_0.25_Mg_0.25_. The annealed materials did not show major shifts for the XRD analysis, only for *M_S_* = Ba_0.33_Sr_0.33_Mg_0.33_ and *M_S_* = Ba_0.25_Sr_0.25_Ca_0.25_Mg_0.25_ the *P4/n c c* lattice parameter *c* was found slightly shifted toward smaller values (Figure S23, Supporting Information). All annealed materials, however, obtained a minorly blue‐shifted emission maxima, compared to the non‐Mg‐doped counterpart, whereas for *M_S_* = Sr_0.33_Ca_0.33_Mg_0.33_ also significantly larger fluorescence lifetimes were detected (Figure S24, Supporting Information). STEM analysis found again strong segregation of the Mg‐containing phases, as well as traces of Mg (< 0.5% atomic fraction) within the non‐segregated copper tetrasilicates NSs (Figures S25–S27, Supporting Information). For *M_S_* = Ca_0.9_Mg_0.1_, different trends during annealing were observed, reaching its maximum during 10 min‐sintering at 1100 °C (Figure S28, Supporting Information). Overall, we attribute the shifted fluorescence properties of the described materials to slight Mg‐introduction, yielding Mg‐doped‐CaCuSi_4_O_10_ Mg‐doped‐Ba_0.5_Sr_0.5_CuSi_4_O_10_, Mg‐doped‐Sr_0.5_Ca_0.5_CuSi_4_O_10_ and Mg‐doped‐Ba_0.33_Sr_0.33_Ca_0.33_CuSi_4_O_10_. However, as the emission maxima is known to depend on the exact group‐II cation ratio,^[^ ^28^ ^]^ the introduction and segregation of Mg could possibly change these aspects in the multielement doping of CTSs. Since similar trends of fluorescence modulation were found in Mg‐doped‐SrCuSi_4_O_10_ and Mg‐doped‐BaCuSi_4_O_10_, we attribute the observed changes to an overall minor Mg‐doping, rather than a modulated alkaline earth metal ratio.

### 2.5 Engineering of Copper Tetrasilicates Shifts Emission to NIR‐II Window

**Figure 5:**
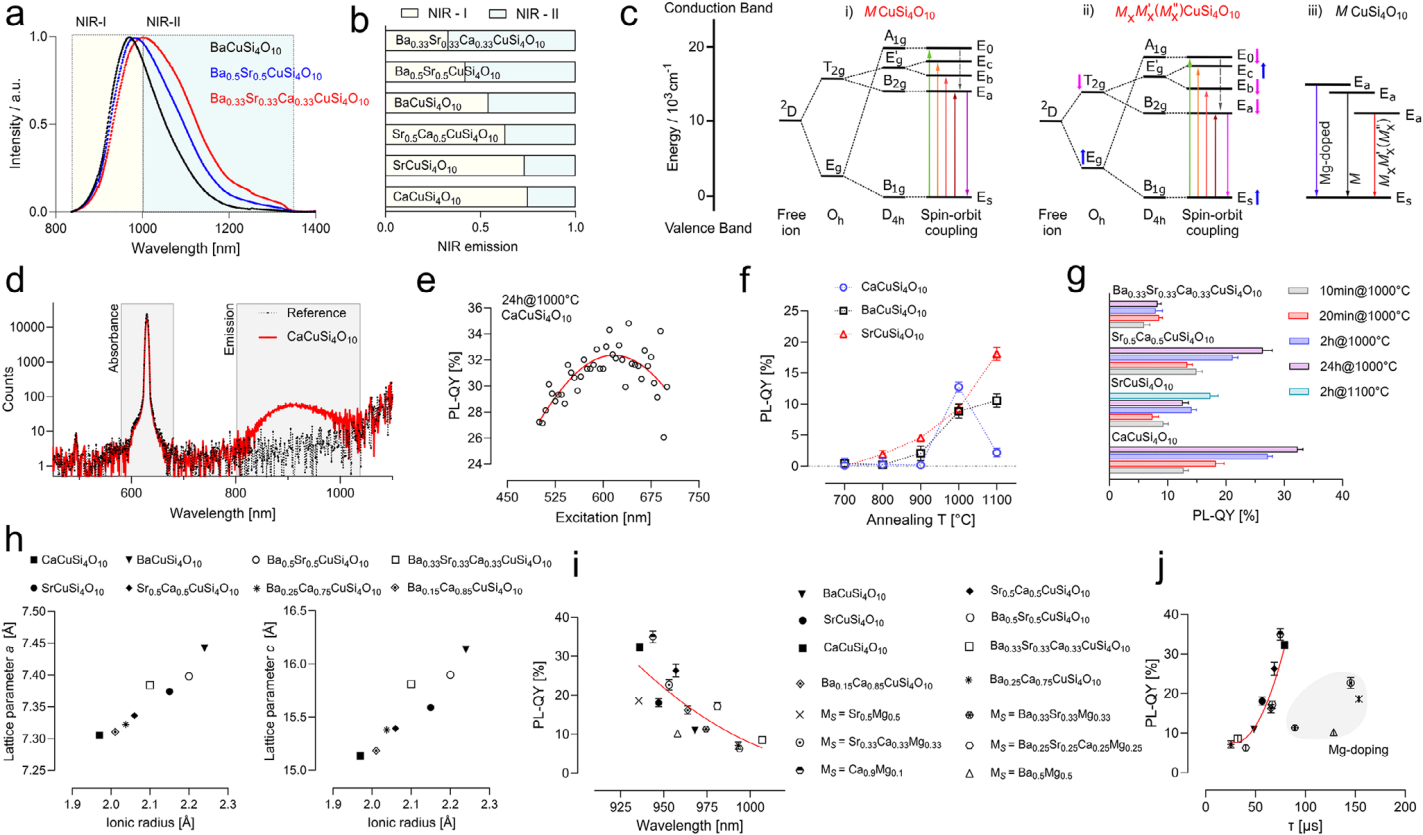
Photoluminescence engineering of copper tetrasilicates enables emission shift to NIR‐II window. a) NIR emission spectrum of BaCuSi_4_O_10_ and its mixed forms showing a significant impact of (multi)element doping toward shifting the emission into the NIR‐II window (> 1000 nm). b) Evaluation of the NIR emission spectra as integrated for NIR‐I (simplified as < 1000 nm) and for NIR‐II (> 1000 nm). c) Simplified energy diagram of Cu^2+^ ion within a tetragonally distorted crystal field, for i) non‐doped, single *M*‐containing NS and ii) multielement doped NS, highlighting the shifted E_a_ energy levels iii). d) Absolute photoluminescence quantum yield (PL‐QY) spectra of CaCuSi_4_O_10_. Integrated photon counts within the gray box, excitation at 630 nm. e) PL‐QY dependency on the excitation wavelengths (red line = Gaussian fit; PL‐QY = 32%). f) PL‐QY engineering through variation of annealing temperature of resynthesized CTS. g) PL‐QY engineering through optimizing annealing time, showing a general trend of increasing PL‐QY with prolonged annealing (mean ± SD). h) Correlation between the lattice parameters *a* and *c*, obtained from Rietveld refinement, and the calculated (mean) ionic radius of the (mixed) alkaline earth metal CTS. i) Correlation of the optimized PL‐QY to the emission wavelengths of all synthesized 2D CTS variations (for comparison with reference values see Figure S30 (Supporting Information), red line = second order polynomial fit). j) Correlation between the PL‐QY and fluorescence lifetime of all obtained materials (red line = second order polynomial fit of non‐Mg containing NS).

After synthesizing this full set of NS combinations, including novel mixed phases of Ba–Ca and Ba–Sr–Ca CTS, their potential for biomedical imaging was assessed. An optimal bioimaging probe would meet two major requirements: a red‐shifted fluorescence emission in the NIR‐II spectral window as imaging modalities improve due to decreased scattering and tissue absorption^[^ ^1^, ^2^ ^]^ and a bright emittance, characterized by a high PL‐QY. Besides the previously mentioned bathochromic shift in emission maxima of the multielement‐doped NS, a general broadening of the emission including a tail reaching far into the NIR‐II was detected (**Figure** **5**a). To quantify the proportion of NIR emission, we subdivided the spectra into two parts, whereas a simplified NIR‐I included the emission up to 1000 nm and the NIR‐II does include all emissions above. As seen from Figure 5b, ≈25% of the emission of SrCuSi_4_O_10_ spans within the NIR‐II, whereas for BaCuSi_4_O_10_ it is 37%. For Ba_0.5_Sr_0.5_CuSi_4_O_10_ already the majority (58%) of the emitted photons are detected within the NIR‐II, whereas a maximum was observed for Ba_0.33_Sr_0.33_Ca_0.33_CuSi_4_O_10_ with 67% and Ba_0.25_Ca_0.75_CuSi_4_O_10_ with 68%. Those observed emission properties translate into altered energy levels for the Cu^2+^ ion, located in the tetragonally distorted crystal field environment, shown as a simplified energy diagram in Figure 5c. For all CTSs with the common structure of *M*CuSi_4_O_10,_ the Cu^2+^ is in square‐planar coordination with four oxygen atoms forming a near‐perfect D_4h_ symmetry due to the Jahn–Teller effect and lattice constraints, whereas in an octahedral ligand field (O_h_ symmetry), the ^2^D ground state of free Cu^2+^ ions split into T_2g_ and E_g_ levels.^[^ ^14^, ^35^ ^]^

This D_4h_ configuration further splits the T_2g_ and E_g_ levels into E_g_, B_2g_, A_1g_, and B_1g_. Additionally, strong spin–orbit coupling causes these energy levels to split further into five distinct levels: E_0_, E_c_, E_b_, E_a_, and E_s_. As for the doped CTS structures, including the mixed forms between Sr–Ca, Ba–Sr, Ba–Ca, and Ba–Sr–Ca a significant bathochromic shift in emission was detected, and the E_a_ energy level needs to be downshifted for multi‐doped CTSs (Figure 5c,ii). This could be caused by a more ordered CuO_4_ tetrahedron, due to impacting the Cu─O bond lengths by replacing the alkaline earth metals within the 2D CTS structure.^[^ ^28^ ^]^ As a consequence, the crystal field splitting of the O_h_ symmetry would be weakened, downshifting the T_2g_ level and upshifting the E_g_ level (Figure 5c,ii).^[^ ^34^, ^36^ ^]^ This would further influence the second splitting of the spin–orbit coupling, overall downshifting the E_a_ energy levels. Similarly, the E_a_ energy levels should increase due to Mg‐doping, as seen for the blue‐shifted emission of Mg‐doped‐SrCuSi_4_O_10_ and Mg‐doped‐BaCuSi_4_O_10_ (Figure 5c,iii).

In the next step, absolute quantum yield spectroscopy was employed to assess the performance and impact of multi‐element doping on the emittance of the different CTSs. Figure 5d shows the PL‐QY spectrum of CaCuSi_4_O_10_ whereas the area for absorbed and emitted photons is highlighted in gray. This direct and absolute method furthermore allows us to evaluate the dependency of the PL‐QY on the excitation wavelengths (Figure 5e), showing maximum emission when excited at 615–630 nm with a mean PL‐QY of 32%. This is in agreement with the maximum of the respective UV–vis–NIR absorption spectra (Figures S6 and S29b, Supporting Information). The observed dependency of the QY on the excitation wavelength could be attributed to excitation‐dependent decay pathways, particularly from the ^2^E_g_ energy level. Using this technique furthermore allows for the photoluminescence engineering of the NS, as optimization of annealing conditions seemed to impact the nanomaterial's emittance significantly. As shown in Figure 5f, different annealing temperatures modulated the PL‐QY of the copper tetrasilicates in a different manner. Emission increases when annealing of the FSP‐particles leads to the desired *P4/n c c* crystal phase and decreases, like in the case of CaCuSi_4_O_10_ if excessive heating led to the degradation of such. However, in the case of SrCuSi_4_O_10_ the PL‐QY was found highest, when sintered at 1100 °C even if structural parameters stayed similar to the material sintered at 1000 °C. In addition to it, also the annealing time was found to drastically impact the PL‐QY of some of the synthesized materials, such as seen for CaCuSi_4_O_10_ or Sr_0.5_Ca_0.5_CuSi_4_O_10_ (Figure 5g), where prolonged annealing improves the emission. For other chemical modifications of the NS structures, such as Ba_0.33_Sr_0.33_Ca_0.33_CuSi_4_O_10_ no significant enhancement could be found (Figure S29, Supporting Information). PL‐QY of 2 h annealed samples were found to be 16% for Ba_0.15_Ca_0.85_CuSi_4_O_10_ and 7% for Ba_0.25_Ca_0.75_CuSi_4_O_10_. This translates to a 50% increased emission of Ba_0.15_Ca_0.85_CuSi_4_O_10_ compared to undoped BaCuSi_4_O_10_, while exhibiting similar fluorescence emission features.

Finally, the structural and optical properties were analyzed across the different phases. Based on the Rietveld refinement of the XRD analysis, the lattice parameters *a* and *c* were plotted against the calculated ionic radii of the alkaline earth metal combinations (Figure 5h). For the newly synthesized Ba–Ca and Ba–Sr–Ca CTS combinations, a minor distortion of the crystal lattice becomes visible, as lattice parameter *c* is slightly shifted toward larger values. Moreover, the mean ionic radii correlated with the unit cell volume and *M*–*M* distances, whereas no straightforward correlation was observed for the *M*─O and Cu─O bond lengths (Figure S30a, Supporting Information). A noticeable trend was observed in the correlation between the lattice parameter *c* and the Cu─O and *M*─O bond lengths (Figure S30b, Supporting Information). When comparing the optimized PL‐QY and the emission wavelengths of each respective CTS, a contrasting trend becomes visible (Figure 5i). The PL‐QY decreases with bathochromic shifted emission maxima, following an overall trend of a second‐order polynomial fit. When comparing the CTS's photoluminescence parameters with previous reports, it becomes evident that the FSP‐synthesized CTS possesses on average significantly higher quantum yields (Figure S30c,d, Supporting Information). Matching the PL‐QY and fluorescence lifetime values resulted in two distinctive groups (Figure 5j). The majority of mixed CTS follows a trend, resembled by a second‐order polynomial fit (R^2^ = 0.89) showing a simultaneous increase of both parameters. However, a second group with larger fluorescence lifetimes was observed, associated with Mg‐doping of the respective CTS structures. We also correlated the CTS lattice parameters with their optical properties by comparing fluorescence emission maxima, QY, and lifetimes with the mean ionic radius, Cu─O bond lengths, mean *M*─O bond lengths, and mean Si─O bond lengths (Figure S31, Supporting Information). However, no clear trends were observed. The bathochromic shift in emission maxima for the doped CTS structures showed only a slight correlation with the Cu─O bond lengths of the square‐planar coordinated Cu^2^⁺ ions but did not exhibit a significant trend when compared to the *M*─O bond lengths.

### 2.6 Laser Irradiation Induced Lattice Rearrangement

**Figure 6:**
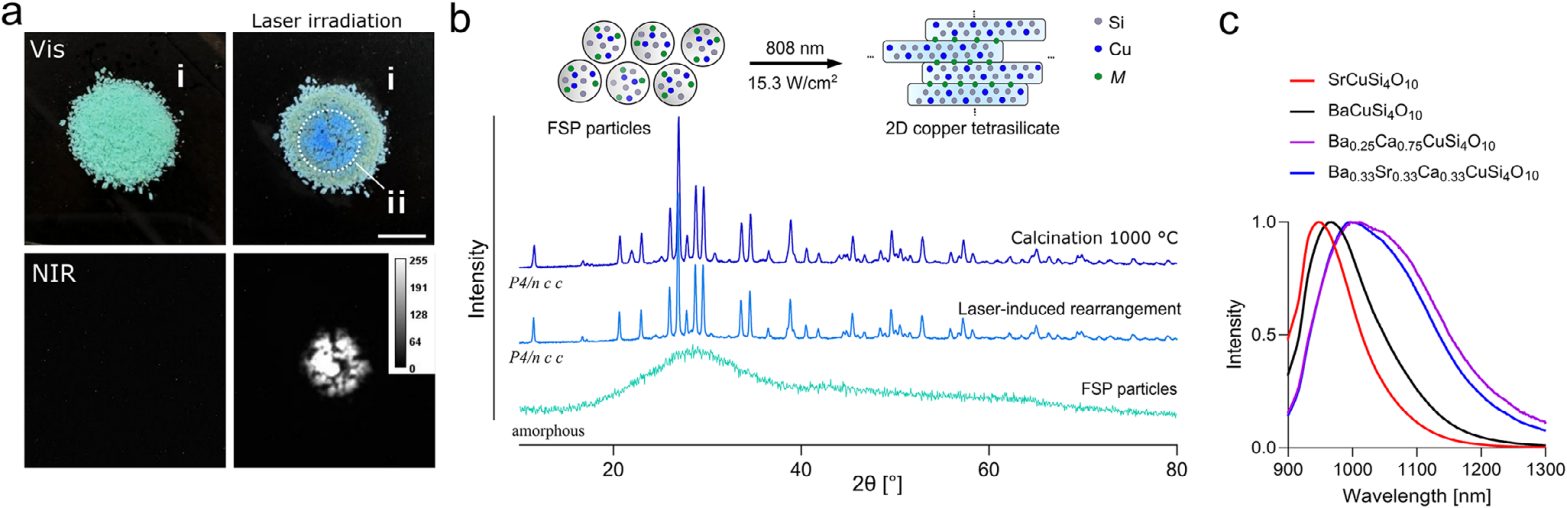
Nanosheet annealing through laser irradiation. a) Photograph of primary FSP particles (i; cyan) rearranged into NIR‐fluorescent SrCuSi_4_O_10_ (ii; blue) through 808 nm laser irradiation (15.3 W cm^−2^, white circle) (scale bar = 0.5 cm). b) Schematic representation of the in situ rearrangement process and XRD pattern of the corresponding particles. The amorphous primary FSP particles anneal within seconds into the characteristic *P4/ncc* tetragonal CTS crystal lattice, similar to a calcination process at 1000 °C (10 min). c) Fluorescence emission spectra of (multielement doped) CTS obtained by laser irradiation.

One of the significant advantages of the FSP‐synthesis process is its ability to decouple elemental mixing from the annealing process. This approach, as described above, creates the platform for multielement doping and rapid sintering processes, enabling the production of metastable CTSs with enhanced emission properties. However, this development prompted the question of whether alternative methods could anneal the FSP primary particles into 2D CTS structures, beyond traditional calcination techniques. Our findings revealed that irradiation of amorphous FSP particles with NIR laser light was sufficient to rearrange their lattice into 2D CTS (**Figure** **6**a). Illumination at a relatively high‐power density of 15.3 W cm^−^^2^ caused the particles to change color from cyan to blue and enabled the detection of strong NIR fluorescence. Remarkably, this stable rearrangement process occurred within seconds, with 2–5s being sufficient to observe a significant emission signal (Figure S32, Supporting Information). It is likely that laser irradiation induces localized heating, which anneals the amorphous primary FSP particles into the characteristic *P4/ncc* tetragonal crystal lattice (Figure 6b). The SrCuSi_4_O₁₀ CTS material obtained via laser irradiation consisted of highly crystalline, interconnected nanosheets with various orientations, as revealed by HR‐TEM investigations.

These nanosheets exhibited crystal lattice spacings of ≈0.8 nm and displayed a minimal amorphous background (Figure S32, Supporting Information). Furthermore, this laser irradiation process can be easily extended to other FSP particles, facilitating the formation of multielement‐doped CTSs with characteristic NIR‐II emission properties (Figure 6c). This localized lattice rearrangement establishes a foundation for in situ, bottom‐up synthesis of engineered nanosheets, paving the way for future advancements in material design.

### 2.7 Brilliant Nanosheets for Super‐Resolution Microcirculation Mapping of the Murine Brain

**Figure 7:**
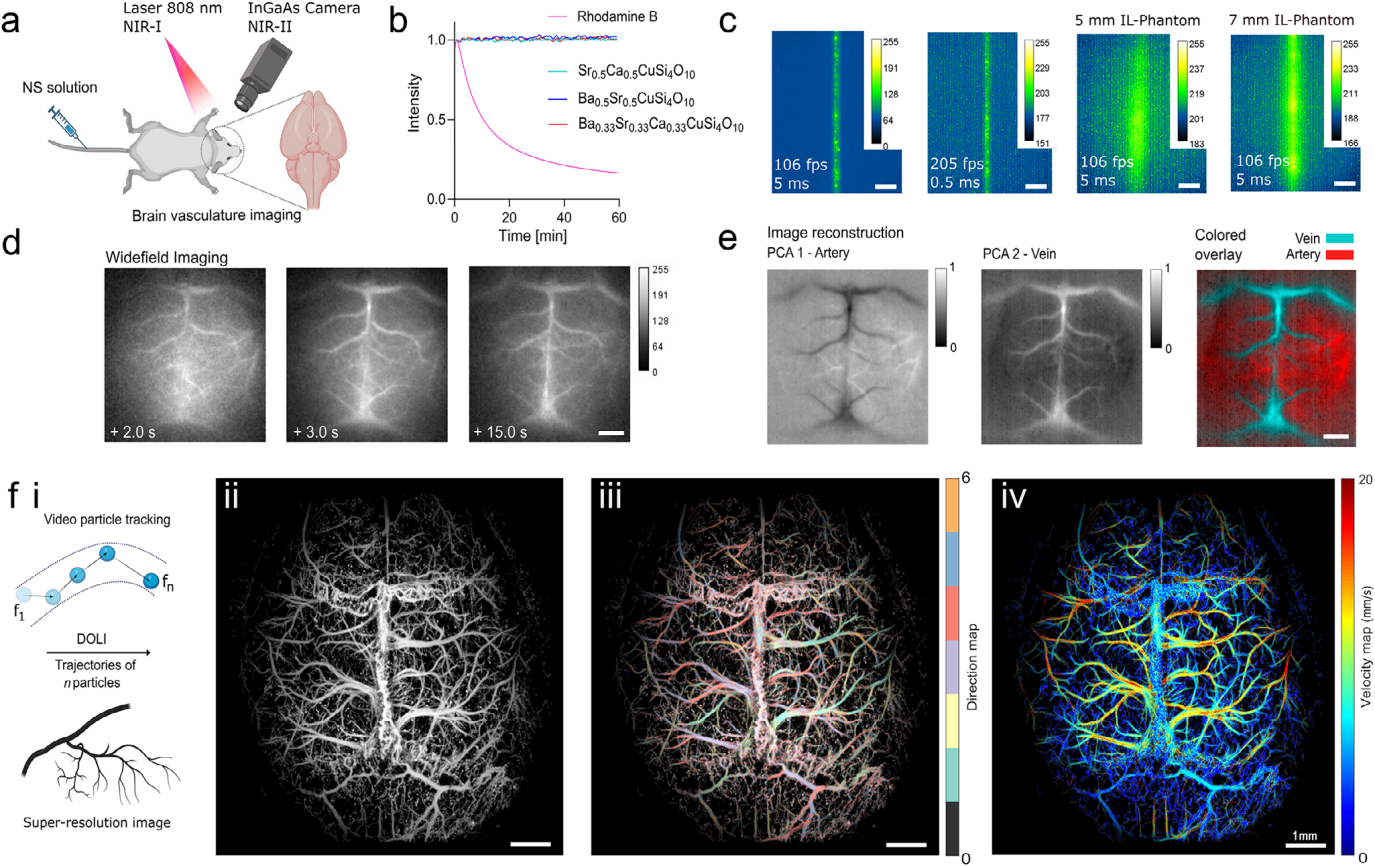
Engineered nanosheets for super‐resolution mapping of the murine brain. a) Schematic of the diffuse optical localization imaging (DOLI) system used for cerebrovascular imaging in the NIR window. A SWIR camera was used to collect the fluorescence emission of a dispersion of stabilized NS injected intravenously (i.v.) under 808 nm excitation (850 mW cm^−2^). b) Photostability of CTS NSs compared to a common organic dye (Rhodamine B). c) High‐frame‐rate imaging of CaCuSi_4_O_10_ NS placed inside a vessel‐mimicking Teflon tube (280 µm inner diameter). Light scattering of brain tissues was mimicked with a 1.2% intralipid (IL) phantom (scale bar = 500 µm). d) Time‐lapse widefield images post DMSA‐stabilized NS injection (scale bar = 1 mm). e) Differentiation of veins and arteries based on their different perfusion patterns, distinguished through principal component analysis (PCA) (scale bar = 1 mm). f) Schematic overview i) of the working principle of DOLI rendering the structural ii), blood flow direction iii) and velocity iv, mm/s) maps of cerebral vasculature from continuous localization and tracking of circulating PEGylated NSs (scale bar = 1 mm).

After obtaining highly fluorescent CTS with engineered emission wavelengths, we evaluated their performance as optical contrast agents for macroscopic brain vasculature imaging. However, to enable their use in vivo, several key challenges had to be addressed to render the CTS‐NS suitable for biological applications. Interestingly, the dispersion of NS in water was found to be highly efficient for the FSP‐derived CTS material, in contrast to the commercial counterpart, while maintaining a high fluorescence (Figure S33a, Supporting Information). We report the PL‐QY of dispersed NS with 13.5%, significantly higher than those generated by mechanical milling processes (Figure S33b, Supporting Information). However, to enable in vivo colloidal stability of the NS after intravenous (i.v.) injection, the surface of the particles was functionalized, as bare NS tended to aggregate in blood. We employed two straightforward strategies to coat the NS: modification with dimercaptosuccinic acid (DMSA) and PEGylation. Both approaches altered the surface charge to more negative values as seen by Zeta‐potential measurements while keeping the size distribution unchanged (Figure S33c,d, Supporting Information). After optimizing the NS dispersion, imaging of the murine brain was performed following i.v. injection of NS, ensuring a homogeneous distribution of NS in the brain vasculature within seconds (**Figure** **7**a). Here, an 808 nm laser was used to excite the NS's B_2g_ energy transition located in the NIR‐I window. Although not being the optimum for NS excitation (see Figure 5e), it offers a balanced compromise between sufficient absorption, enhanced tissue penetration, and minimal photodamage, while obtaining a similar emission profile (Figure S33e, Supporting Information). A short‐wave infrared (SWIR) camera enabled large‐scale detection of fluorescence emission across the entire cortex from ≈20 cm distance above the mouse through the intact skull. An additional feature making these NS promising candidates for imaging applications is their significantly superior photostability compared to organic dyes. The synthesized NS, including the multielement‐doped ones, demonstrated resistance to photobleaching (Figure 7b). High‐frame‐rate imaging of various NS perfused through a vasculature‐mimicking microtubing served to determine the most optimal NS candidate. CaCuSi_4_O_10_ NS were found to exhibit a particularly prominent performance due to their brightest emission, allowing visualization with over 200 fps (Figure 7c) under light exposure conditions suitable for in vivo imaging. Blurring of the acquired fluorescence images was observed when an intralipid (IL) phantom simulating light scattering in brain tissues was placed above the microtubing, as evidenced by an enlarged point spread function (Figure 7c, right).

More interestingly, i.v. injection of the NS in mice revealed that surface modification can strongly impact their performance as contrast agents. Two phases were distinguished following i.v. injection of DMSA‐stabilized and PEGylated NS. The bolus of particles first appeared to uniformly distribute across the vasculature in the collected images in a similar manner to a conventional fluorescent dye (Figure 7d; Figures S34 and S35, Supporting Information). PEGylated CTS‐NS demonstrated widefield imaging performance almost identical to the clinically used contrast agent ICG (Figure S35b, Supporting Information). High‐frame‐rate imaging (69 fps) in this phase enabled the differentiation of the vascular tree into veins and arteries, based on their different perfusion patterns (Figure 7e; Figures S34a and S35a, Supporting Information). This initial NS bolus was followed by dilution of the NS in blood, which resulted in dots distinguishable in the images corresponding to individual or small clusters of NS circulating in the bloodstream (Video S1, Supporting Information). This sparse distribution of circulating particles enabled their individual localization and tracking using the recently reported diffuse optical localization imaging (DOLI) method. By continuously tracking the trajectory of numerous particles, a super‐resolution and background‐free vascular image could be reconstructed (Figure 7f,i). However, the DMSA functionalized NSs were found to circulate for less than a minute before getting cleared completely from the bloodstream, resulting in a fragmentary DOLI image (Figure S34b,c, Supporting Information). On the contrary, PEGylated NS exhibited an extended circulation time. After injection, up to ≈20 000 individual particles per 10 s were detected on the whole cerebral region, whereas 90% were cleared within the first 1.5 min. About 5% of the initial particles were found to circulate continuously, translating into the localization of several hundred particles even 10 min post‐injection (Figure S35d,e, Supporting Information). The reconstructed DOLI images (Figure 7f; Figure S35c, Supporting Information) then revealed cortex‐wide brain vasculature networks in great detail (f,*ii*). Beyond structural information, blood flow direction (f*iii*) and velocity (f*iv*) maps could be reconstructed, revealing regions with circulation speeds exceeding 10 mm s^−1^. This demonstrates the potential of NS to function as fluorescence‐based velocity sensors in the NIR‐II window. To address the aspect of NS biocompatibility, we harvested key organs after the terminal experiments and evaluated potential tissue damage through histological examinations. As shown in Figure S36 (Supporting Information), no significant organ damage was observed, however, extended biotoxicity tests are needed in the future to understand their biosafety beyond terminal experiments.

### 2.8 Nanosheet‐Labeled Macrophages Enable Large‐Scale Single‐Cell Tracking

**Figure 8:**
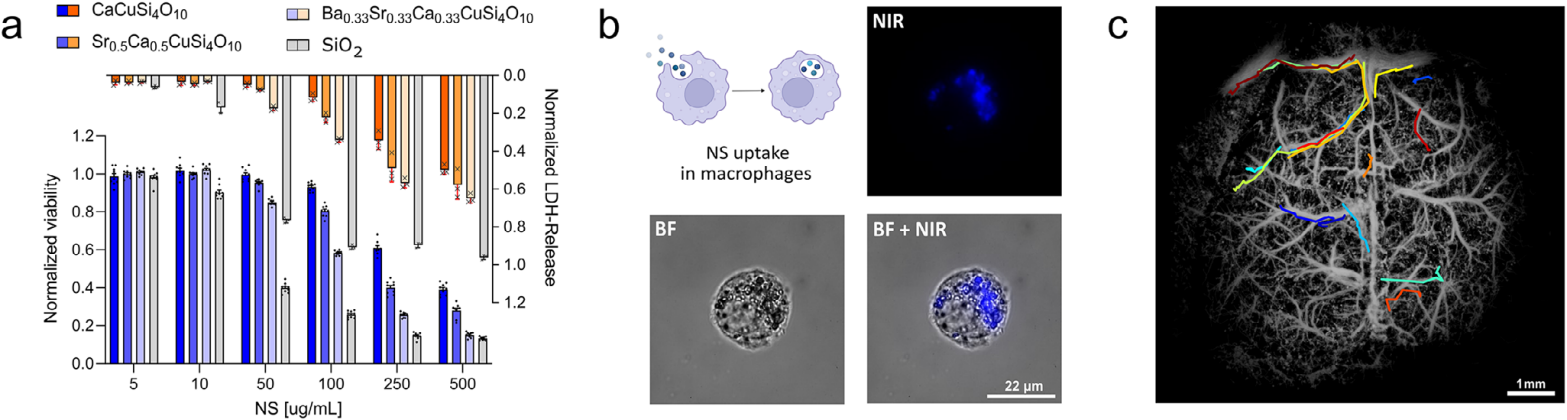
Individual macrophage tracking in vivo. a) Macrophage cell toxicity test for various NS compared to SiO_2_ (Aerosil 90; mean ± SD). b) Schematic representation of NSs uptaken by human macrophages, with respective bright field (BF) and NIR‐fluorescence images of a single NS‐labeled cell. c) Overlay of all tracked macrophages (N = 15) resemble parts of the vasculature tree (DOLI image from Figure S35c, Supporting Information; scale bar = 1 mm).

Inspired by the bright emission of trackable NS, we aimed to use them as a NIR‐fluorescent label for immune cells, potentially enabling single‐cell tracking within the macroscopic imaging system. However, for effective macrophage loading, the NS needed to be applied at the highest possible concentrations, which were determined within a cell viability assay, combined with a marker identifying cell membrane damage. As shown in **Figure** **8**a, we compared undoped and multielement doped NS with bare SiO_2_, identifying the CTS as generally more compatible. CaCuSi_4_O_10_ NS was found to exhibit minimal effects on cell viability up to a concentration of 100 ug mL^−1^, whereas for Ba_0.33_Sr_0.33_Ca_0.33_CuSi_4_O_10_ only half of the cells survived at the same concentration. Cell membrane damage followed a complementary trend, showing increased lactate‐dehydrogenase (LDH) release with decreasing cell survival. Moreover, PEGylating the NS surface further improved cytocompatibility, compared to the non‐functionalized NS (Figure S37, Supporting Information), making them a suitable candidate to boost the overall uptake. Extensive washing was applied, to remove NS not uptaken by the cells from the culture, while loaded macrophages were detached from the culture well surface and fixed as single‐cell suspension, functioning as a carrier for the luminescent cargo. Figure 8b shows a (fluorescence) image of a single macrophage on a microscopic scale, that appears to internalize the NS likely via phagocytosis. When performing high‐frame‐rate imaging tests in the previously mentioned capillary system, single fluorescent spots were clearly visible, getting blurred underneath an intralipid tissue phantom (Figure S38, Supporting Information). These cells were then re‐injected into the mouse model for brain vasculature imaging. A snapshot of a moving cell is presented in Figure S38c (Supporting Information), together with a comparative video of the raw data in Video S2 (Supporting Information), demonstrating that the detected fluorescence signal was strong enough to enable tracking across multiple frames. This allowed us to capture the trajectories of several cells, which remained visible at the cerebral scale for 5–40 frames, corresponding to less than 1 s at a frame rate of 69 fps. When these trajectories are plotted and overlaid onto the high‐resolution vascular image, it becomes clear that macrophage movement closely follows sections of the vascular tree (Figure 8c; each colored marking represents the trajectories of individual macrophages).

## 3 Discussion

The development of new high‐performance NIR‐II contrast agents is set to significantly advance the diagnostic and therapeutic potential of non‐invasive optical imaging and paves the way for molecular imaging. In this context, the introduced FSP‐synthesis route offers a promising approach to compositionally engineer CTSs, creating highly fluorescent nanomaterials suited for bioimaging and biosensing. This modular FSP approach presents several advantages over previous synthesis methods, particularly in eliminating unwanted elements introduced during the synthesis process. By enabling the residual‐free combustion of metal–organic precursors, this technique produces high‐purity CTSs. In contrast, traditional methods such as the melt‐flux technique rely on inorganic salts and flux agents like soda or plant ash to lower the melting point of silica, often leaving contaminants within the material. These contaminants can result in the formation of unwanted crystal phases, including a glassy background, which introduces defects at grain boundaries and diminishes the fluorescence properties of the nanomaterial.^[^ ^37^ ^]^ Moreover, the FSP process can be scaled up to an industrial level, enabling the production of nanoparticles at rates ranging from hundreds of grams per hour to several kilograms per hour.^[^ ^38^ ^]^ The FSP‐derived material itself enables precise study of temperature‐dependent crystal phase transitions and their kinetics. For example, previous reports describe the formation of cuprorivaite powders taking several hours at 1000 °C, annealing the primary FSP material yields CTSs within minutes. This suggests that with a homogeneous elemental distribution, the rearrangement into a 2D crystal lattice occurs ≈2 orders of magnitude faster than previously documented.^[^ ^14^ ^]^ This rapid process forms the basis for effective chemical doping strategies, as it significantly reduces the need for prolonged sintering times, thereby minimizing unwanted phase segregations. Future approaches could further refine this technique, potentially allowing for the bottom‐up synthesis of low‐dimensional NSs through uniform annealing within a circulating gas stream, avoiding excessive sintering of material clusters. Most interestingly on the way to NIR‐emission engineered contrast agents, the FSP‐synthesis allowed for the first time multi‐alkaline earth metal doped CTSs. Uniting Ba and Ca, and Ba, Sr, and Ca within the lattice structure was observed to shift PL emission into the NIR‐II spectral window. Such shift could be attributed to modulation of the square‐planar coordinated Cu^2+^ in the distorted crystal field, as an expansion of the Cu─O bond length and more ordering of the CuO_4_ tetrahedron could occur, resulting in a lowered transition energy of the doped‐NS.^[^ ^28^ ^]^ Previous studies found, that exchange of cations seems to induce a change in the crystal field environment around Cu^2+^ ions, impacting the emission wavelength, however only comparing undoped *M*CuSi_4_O_10_ and a series of Sr_x_Ca_1‐x_CuSi_4_O_10_.^[^ ^28^, ^32^, ^39^ ^]^ As the strongest bathochromic PL shifts were detected in (meta‐stable) NS that unite Ca and Ba in the crystal lattice, we hypothesize, that the substantial change in Ca─O and Ba─O bond lengths (≈0.4 Å),^[^ ^33^ ^]^ may locally distort the crystal lattice, partly contributing to the weakening of the crystal field and hence the pronounced shift in fluorescence emission. These local domains of distorted CTS bond lengths could significantly impact the Cu^2^⁺ environment without being reflected in the averaged *M*─O bond lengths used for PL comparison. However, although previous studies^[^ ^28^, ^33^ ^]^ have indicated that Cu─O bond lengths can increase substantially in doped CTS structures compared to undoped *M*CuSi_4_O_10_, we did not observe a strong correlation between the largest bathochromic emission shifts and the largest Cu─O bond lengths. Nonetheless, the incorporation of Ba and Ca in the crystal structure lead to an anisotropic increase in cell dimensions, as Δ*c* > Δ*a*, related to the inflexibility of the silicate anion within the corner‐linked Si_4_O_10_ rings.^[^ ^27^ ^]^ An impact of oxygen defects was discussed earlier, leading to a local distortion of the alkaline earth metal layer and an improvement of the *M*─O bond strengths.^[^ ^27^ ^]^ We speculate that doped CTS structures may be more prone to oxygen intercalation and the formation of oxygen defects due to local distortions caused by the incorporation of *M* ions of varying sizes into the crystal lattice. This could lead to an overall weakening of the crystal field and the environment surrounding Cu^2^⁺, resulting in the observed bathochromic shift in emission. Since also the electronic states of the Cu^2^⁺ ion are strongly coupled to the vibrational modes of the crystal, doping the lattice may influence vibronic interactions and the resulting energy transitions.^[^ ^40^ ^]^ However, specialized density functional theory (DFT) calculations will be required in the future to gain a fundamental understanding of the bandgap structure in doped CTS materials.^[^ ^35^ ^]^

Moreover, slight doping of the crystal lattice with Mg was achieved. While this doping does not significantly alter the overall lattice parameters, it does induce a noticeable shift in photoluminescence emission toward higher energies and an accompanying increase in fluorescence lifetime. This hypsochromic shift in emission aligns with the modulation of Mg─O bond lengths, as the shorter Mg─O bonds could strengthen the crystal field, thereby increasing the transition energy in the Mg‐doped CTSs. Overall, the increased emission lifetime and hypsochromically shifted emission could be attributed to a slight lattice contraction, which may reduce the likelihood of energy transfer from the activated ions to defects in the contracted lattice.^[^ ^34^, ^41^ ^]^ Building on these findings, we have developed a new series of optically active materials, which hold potential for use as multi‐color NIR‐fluorescent lifetime labels in future applications.^[^ ^42^ ^]^ We overall present a comprehensive set of CTS structures, enabling robust comparison of their optical properties. The observed enhanced photoluminescence properties of the FSP‐derived CTS material may be attributed to a reduced occurrence of vacancies within the crystal lattice and grain boundaries, which lowers non‐radiative rate constants and consequently increases fluorescence lifetimes and quantum yields.

This foundational work expands the range of available NIR‐emission tailored materials, as the FSP synthesis approach opens avenues for studying additional multi‐element systems. This includes the substitution of ions like Zn or La,^[^ ^43^ ^]^ and the development of functional hybrid structures through doping with lanthanides, possibly extending emission wavelengths and unlocking advanced NIR imaging modalities.^[^ ^44^ ^]^ Streamlining the FSP process into an automated, AI‐driven, continuous synthesis platform could facilitate rapid and effective screening of engineered nanostructures in the future, extending applications beyond merely improving emission properties.^[^ ^45^, ^46^ ^]^ In addition to the annealing of CTS from FSP‐derived primary particles via calcination, we report a laser‐induced transformation of the crystal lattice. This process facilitates a stable lattice rearrangement into the 2D structure within seconds, shedding light on the kinetics involved, ≈2 orders of magnitude faster than the minutes‐long calcination process described earlier. This rapid transformation provides a promising platform for future studies, ranging from the in situ formation of luminescent CTSs to the microscopic annealing of individual nanosheets. By accessing highly fluorescent nanostructures and applying biocompatible surface modifications, the engineered NS were successfully employed as in vivo contrast agents in an animal model. To the best of our knowledge, this represents the first reported in vivo bioimaging application for the entire class of CTS materials. This achievement demonstrates the potential applications beyond insect systems,^[^ ^11^ ^]^ showing that small, highly luminescent particles can be obtained without the need for elaborate mechanical degradation of bulk CTS material, known to significantly degrade PL emission and obstruct bioimaging applications.^[^ ^23^ ^]^ Previously reported NIR‐II‐emitting quantum dots, such as InAs (PL‐QY ≈30%) and PbS‐based QDs, have demonstrated capabilities for blood flow velocity reconstruction and DOLI‐based super‐resolution imaging.^[^ ^4^, ^47^ ^]^ Here, we demonstrate that our heavy metal‐free CTS‐NS provide comparable imaging performance, with the added advantage of visualizing fine skull vessels, while avoiding the inherent toxicity associated with heavy metal‐based systems. However, refining the particle size to produce smaller CTS‐NS could be employed in the future to minimize immune cell clearance, thereby prolonging circulation lifetimes and further enhancing in vivo performance. Advancements in the optical detection setup could even enable depth‐resolved 3D localization^[^ ^48^ ^]^ or real‐time augmented fluorescence visualization.^[^ ^49^ ^]^ By targeted functionalization of the NS surface with cell receptor binding units or antibodies, highly specific molecular imaging could be achieved, advancing from in vitro systems to in vivo cancer models.^[^ ^50^ ^]^ The bright emission and biocompatibility of the synthesized NS were further demonstrated through their uptake by macrophages and subsequent in vivo tracking. To the best of our knowledge, this represents the first instance that single immune cells have been imaged in vivo, in real‐time, on a cortex‐wide scale. This lays the foundation for functional and molecular imaging with the proposed approach, including the labeling of other immune cells like T‐cells to track their interactions with malignant tumors or to explore their roles within the tumor microenvironment. However, further research beyond terminal in vivo experiments is required to assess the long‐term biocompatibility of CTS‐NS. Beyond their potential application in biomedical imaging, these engineered CTS could be utilized in light‐emitting devices, such as those for telecommunication platforms, as security inks, or luminescent solar concentrators. In conclusion, this work employs the tunable synthesis approach of flame‐spray‐pyrolysis to develop highly fluorescent, emission‐engineered NSs, well‐suited for next‐generation in vivo bioimaging. The successful transcranial mapping of cortical microcirculation in mice and the tracking of immune cells across the cerebral region further highlights the promise of FSP‐synthesized 2D‐CTS as powerful NIR‐II emissive agents for advanced bioimaging and beyond.

## 4 Experimental Section

### Chemicals

All materials, if not stated otherwise, were purchased from Sigma–Aldrich or VWR Chemicals.

### Flame‐Spray‐Pyrolysis

Organic precursor solutions were prepared to match the elemental composition of the desired copper tetrasilicate, hence a ratio of *M*:Cu:Si was set at 1:1:4 with *M* = Ca, Sr, Ba or Mg. Metal‐acetate precursors were dissolved in 2‐ethylhexanoic acid (2‐EHA) for 2 h at 130 °C while constant stirring under reflux. Borchers Deca Copper 8 was used as Cu precursor and hexamethyldisilane as Si precursor. Conditions for an optimized batch are given in the Table S2 (Supporting Information). Metal–organic precursor solution in 2‐EHA:tetrahydrofuran (THF) (1:1) was injected through a needle into CH_4_/O_2_ flame (5 mL min^−1^ liquid‐feed precursor stream; 5 L min^−1^ O_2_; 3 L min^−1^ CH_4_) and resulting nanoparticles were collected with a fine glass fiber filter located 70 cm above the flame and sieved through a 200 µm mesh. Annealing of the nanomaterial was performed between 700 and 1100 °C for 10 min, using a TGA (METTLER TOLEDO TGA/DSC 3+; 25 k min^−1^ ramp; 40 mL min^−1^ air flow) or at ambient atmosphere for 2–24 h at 900–1100 °C (Sintering furnace Type 4800, Thermolyne). Laser‐induced annealing was performed with 808 nm CW laser (15.3 W cm^−2^; FC‐808‐20 W, CNI, China) fiber coupled to a collimator (SM‐A905 CNI, China) homogenously illuminating the FSP‐derived primary particles within a distance of ≈1 cm for several seconds.

### Photoluminescence and Absorbance Spectroscopy

NIR fluorescence emission spectra were recorded for 0.5 s with a Shamrock 193i spectrometer (slit width = 500 µm) equipped with an InGaAs detector (Andor Technology Ltd., Belfast, Northern Ireland) using a 561 nm Cobolt Jive laser (20 mW, Cobolt AB, Solna, Sweden) as excitation source. A monochromator (MSH150 instrument, equipped with a LSE341 light source, LOT‐Quantum Design GmbH, Darmstadt, Germany) was used for excitation (5 nm steps, 1 s integration time) to obtain 2D‐NIR excitation‐emission spectra. In addition, NIR fluorescence emission spectra were recorded with a NIRQuest+1.7 spectrometer (slit width = 200 µm; InGaAs detector, OceanOptics), fiber‐coupled to a customized Axiovert 40CFL using a 10x objective, 800 nm dichroic mirror (Edmund optics) and 900 nm LP filter (FELH0900, Thorlabs). Excitation was performed with a fiber‐coupled 750 nm CW laser (3 mW, FC‐750‐5 W, CNI, China) and a 785 nm CW laser (3 mW, SLOC lasers, IRM785TA‐3000Fc). Absolute photoluminescence quantum yield (PL‐QY) spectroscopy was performed with a Hamamatsu Quantaurus‐QY (C11347‐12) using solid‐phase quartz glass sample holders. The excitation scan regime was set to 500–700 nm (steps of 5 nm), while the area to detect all emitted photons was adjusted to cover the entire emission. The spectrometer software was used to calculate the PL‐QY, defined as the ratio of the number of photons emitted by the sample to the number of photons absorbed, without the need for reference standards. Absorbance spectroscopy was performed on powder samples using a UV–vis–NIR spectrometer (Cary 5000, Instrument version 3.07; Agilent) scanning from 400 to 850 nm in steps of 1 nm, using a Praying Mantis (HARRICK) sample holder.

### Fluorescence Imaging

For NIR fluorescence photobleaching experiments, fluorescence microscopy was performed using a custom‐made setup containing a 561 nm laser (Jive 500, Cobolt AB, Sweden) and an inverted microscope (IX73, Olympus, Japan) equipped with a 20x objective (MPlanFL N 20, Olympus, Japan). The fluorescence signal was split using a dichroic mirror (R785, AHF Analysentechnik, Germany), and the fluorescence then was captured using two cameras, a cMOS camera (Andor Zyla 5.5, Oxford Instruments, UK) for visible range signals and an InGaAs camera (Xeva 1.7‐320, Xenics, Belgium) for NIR signals. Photobleaching was tested with dispersed NS (1 mg mL^−1^ in H_2_O) and Rhodamine B (1 mg mL^−1^ in H_2_O). 100 µl of the dispersions/solutions were put on glass bottom petri dishes, dried, and imaged at 400 mW excitation power every 15 s for 1 h. The fluorescence intensity corresponds to the mean gray value of averaged over 5 imaged nanoparticles imaged by the NIR camera (NS) or the mean gray value of the complete illuminated area imaged by the VIS camera (Rhodamine B), each after background correction. Camera‐based fluorescence imaging of CTS annealed via laser irradiation was performed as described before,^[^ ^51^ ^]^ using a Ninox SWIR 640 (InGaAs camera, Raptor Photonics) equipped with a StingRay Optics 25 mm SWIR SR2343‐A01 lens and a 900 nm long pass filter (FELH0900, Thorlabs), while images were recorded using the EPIX XCAP V3.8 software at 10 ms integration time. Excitation was performed with a 785 nm CW laser (SLOC lasers, IRM785TA‐3000Fc; 50 mW cm^−2^) fiber‐coupled to a collimator (SMA905 CNI, China) and equipped with a 850 nm short pass filter (FES0850, Thorlabs).

### Fluorescence Lifetime Measurements

For fluorescence lifetime measurements in the frequency domain, a fiber‐optic oxygen sensor (FireSting O_2_, PyroScience, Germanay) was used.^[^ ^21^ ^]^ The sensor emits light with an excitation wavelength of 620 nm; allowing for modulation of the excitation frequency between 100 and 16,000 Hz and detects induced fluorescence. The phase shift between the excitation signal and the detected fluorescence was extracted from the sensor raw data to calculate fluorescence lifetimes. Measurements were performed on dry samples, with the FireSting optical probe mechanically positioned at a fixed distance from the sample of a few milligrams of sample powder placed in a reaction tube. Measurements were conducted at constant conditions and settings (excitation frequency: 4000 Hz, LED intensity: 60%, amplification factor: 400x, signal intensity ≈14 mV). Each sample was measured for 1 min at 1 fps.

### X‐ray Diffraction (XRD)

X‐ray diffraction measurements (XRD) were performed with a Bruker D2 2nd Gen Phaser (30 kV, 10 mA, SSD160 detector, Cu Kα radiation [λ = 1.5418 Å] at 2θ = 10°−80° with a step size of 0.01°). For Rietveld analysis, additional XRD measurements were performed at room temperature on a Malvern PANalytical X`Pert Powder instrument in Bragg–Brentano geometry with Ni‐filtered Cu Kα radiation [λ = 1.5418 Å]. The powders were grinded before measurement and the diffraction patterns were acquired in an angular range of 5–120° 2θ with a measurement time of 19 h per sample. Additional phases besides the expected main “P4/ncc” tetragonal phase were identified with the *Search&Match* option within the software X'Pert HighScore.^[^ ^52^ ^]^ Rietveld analysis with the identified phases was performed using the software TOPAS.^[^ ^53^ ^]^ The crystallographic information files (CIFs) for the refinement were retrieved from the Inorganic Crystal Structure Database (ICSD).^[^ ^54^ ^]^ For the identified phases, Table S3 (Supporting Information) lists the mineral name, chemical formula, crystal system, space group, and ICSD code. Besides the lattice parameters, the scale factors defining the Rietveld quantification and peak shapes were modeled with the Thompson–Cox–Hastings pseudo‐Voigt (TCHZ) model. Preferred orientation in the main “*P4/ncc*” phase was accounted for with spherical harmonics. Retrieved lattice parameters and quantification are given in the Supporting Information. It should be noted that the estimated standard deviations (σ) on these parameters from Rietveld refinement represent rather the precision of these parameters than the accuracy^[^ ^55^ ^]^ and the estimated probable error is rather 2–3σ.^[^ ^56^, ^57^ ^]^ Further, a silicon standard was routinely measured after any configurational changes of the diffractometer. A deviation in the peak position of the (004)‐reflection of 0.003° 2θ was observed within the time frame in which the here presented samples were investigated, which would represent the uncertainty related to the instrumental setup.

### Electron Microscopy

Scanning electron microscopy (SEM) combined with energy‐dispersive X‐ray spectroscopy (EDX) was conducted using an Axia Chemisem (Thermo Fisher, NL). Prior to analysis, the samples were coated with a 10 nm layer of carbon using a Leica EM ACE600. For transmission electron microscopy (TEM) analysis, nanoparticles were dispersed in ethanol, drop‐cast onto nickel grids (EMR, Holey Carbon Film 300 Mesh), air‐dried, and imaged at various magnifications with a Zeiss EM 900 microscope (Carl Zeiss Microscopy GmbH, Germany) operating at 80 kV. Particle size distributions were determined using ImageJ (version 1.54b). These grids were further examined with high‐angle annular dark‐field (HAADF) scanning transmission electron microscopy (STEM) paired with energy‐dispersive X‐ray spectroscopy (EDX) on a Talos F200X TEM microscope (FEI), utilizing four detector configurations (Super‐X EDS) at 200 kV.

### Nanosheet Dispersion and Surface Modification

Typically, 50 mg NP was grinded with a mortar and dispersed in H_2_O (5 mg mL^−1^, 3 min 90% amplitude, Sonics Vibra cell VCX 500, cup horn) followed by 3 min cup‐horn sonication (Sonics Vibra cell VCX 500, CV334 converter). Surface modification with meso‐2,3‐Dimercaptosuccinic acid (DMSA) and via PEGylation was performed by adapting a previously reported protocol.^[^ ^58^ ^]^ Surface grafting with DMSA follows a previous description.^[^ ^59^ ^]^ In brief, 50 mg of respective CTS material was dispersed in 10 mL H_2_O, followed by the addition of 15 mL of DMSA solution (1 mg mL^−1^ in H_2_O). To ensure complete dissolution, the DMSA solution was gently heated prior to addition. The resulting mixture was stirred at 300 rpm for 2.5 h at RT, then centrifuged at 7100 rpm for 20 min. After discarding the supernatant, the pellet was redispersed in 25 mL of H_2_O, and the pH was adjusted to 10 using NaOH while stirring at 300 rpm for 1 h. Finally, the pH was lowered to 7.3, and the mixture was stored at RT. NS fraction was collected as the supernatant after 6 h of sedimentation. For PEGylation, 100 mg of CTS particles were dispersed in 30 mL of ethanol (10 min 95% amplitude, Sonics Vibra cell VCX 500, cup horn), followed by the addition of 1 mL of PEG‐1000‐Si and 100 µL of 32% ammonia solution, based on a reported synthesis protocol for PEG‐silane coupling and subsequent silica nanoparticle modification.^[^ ^60^ ^]^ This mixture was heated under reflux at 75 °C for 24 h. The resulting nanoparticles were centrifuged (10 min, 5000x g) and sequentially washed with 20 mL each of H_2_O, ethanol, THF, ethanol, and H_2_O. The PEGylated material was then dispersed in H_2_O for further use, with the NS fraction collected as the supernatant after 30 min of sedimentation.

### Nanoparticle Characterization

Hydrodynamic size measurements via dynamic light scattering (DLS) and zeta potential determination were conducted using a Zetasizer Nano‐ZS (Malvern Instruments). The surface area was measured using the Brunauer–Emmett–Teller (BET) method at 77 K with a Micromeritics Tristar II Plus.

### Cell Experiments

Human monocytes (THP‐1, ATCC) were cultured in standard conditions at 37 °C in a humidified atmosphere containing 5% CO_2_ and sub‐cultured at 70% confluency. In vitro nanomaterial toxicity and viability of macrophages after 24 h nanomaterial incubation was performed using the LDH‐release (G1780, Promega) and ATP quantification CellTiter‐Glo luminescence assay (G7570, Promega), respectively. In brief, 40,000 THP‐1 cells in 100 uL RPMI‐1640‐Medium (R8758, Sigma–Aldrich, supplemented with 10% FCS, 1% l‐glutamine, 1% Penicillin‐Streptomycin) and 200 nm PMA were seeded in black 96 well plates with transparent bottom. After 3 days, the cell medium of the differentiated macrophages was replaced with PMA‐free cell medium. One day after, the cell medium was exchanged with nanomaterial containing cell medium or the vehicle control medium. All conditions contained 10% milliQ water. After 24 h nanomaterial incubation, 50 uL of supernatant was used for LDH‐release quantification. The assay was performed according to the manufacturer's specifications. For the viability assay, the remaining cell medium was exchanged with fresh cell medium, and CellTiter‐Glo reagent was added (1:1). After 10 min shaking in the dark, luminescence was quantified using a microplate reader (Berthold LB‐943). For macrophage loading with NS, 1 million cells were initially seeded into 6‐well plates in 3 mL cell medium containing 200 nm PMA. The cell medium was replaced with PMA‐free cell medium 3 days after. The next day, nanomaterials solutions were added reaching the desired concentration and a maximum of 10% H_2_O content. A concentration of 200 µg mL^−1^ PEGylated NS was used to label cells for later in vivo tracking experiments. After 24 h nanomaterial incubation, cells were washed twice using PBS and then trypsinized using TrypLE. Additionally, cells were fully detached using a cell scraper. Thereafter, cells were fixed at room temperature using 4% PFA, creating a single‐cell solution. After washing away PFA using PBS twice, fixed and NS‐loaded cells were suspended in PBS containing 1 mm EDTA.

### Animals

Athymic nude‐Foxn1nu mice (N = 4; Envigo BMS B.V., Netherlands) were used for in vivo cerebral microcirculation imaging. The mice were housed in ventilated cages with ad libitum access to food and water. The housing room was kept under a 12 h dark/light cycle, 22 °C room temperature, and ≈50% relative humidity. All animal experiments were performed in accordance with the Swiss Federal Act on Animal Protection and approved by the Cantonal Veterinary Office Zurich. For in vivo imaging, the mice were anesthetized with isoflurane (5% for induction and 1.5% for maintenance) in a mixture of O_2_ and medical air with flow rates of 0.2 L min^−1^ and 0.8 L min^−1^, respectively. For transcranial imaging, the scalp of a mouse was removed post subcutaneous injection of analgesics (Buprenorphine, 0.1 mg kg^−1^). Widefield recording was performed during and after intravenous injection of 100 uL NS solution (10 mg mL^−1^) for a total duration of 10 min. In vivo single‐cell tracking was realized by injecting a total volume of 100 uL PBS solution, containing ≈8000 NS‐loaded macrophages per uL.

### Diffuse Optical Localization Imaging (DOLI) in the NIR‐II Window

DOLI was performed with a custom‐built widefield microscope, as described in detail previously.^[^ ^47^ ^]^ Briefly, an 808 nm CW laser (FC‐808‐20 W, CNI, China) with fiber output was employed to provide epi‐illumination on the sample, with beam size adjustable with a collimator (CNI, China). The fluorescence emission was then collected by the camera lens (LM50HCSW, Kowa, Japan), filtered with a 950 nm long‐pass filter (FELH0950, Thorlabs, USA), focused on the InGaAs‐based SWIR camera (WiDy SenS 640V‐ST, NiT, France). The collected widefield image stack was processed using a localization‐based image reconstruction pipeline, as previously described.^[^ ^47^ ^]^ Here, each frame was first denoised, followed by the localization of individual moving particles. These particles were subsequently tracked across the image stack using u‐track algorithm.^[^ ^61^ ^]^ Localization, tracking, and quantification of circulating nanosheets were performed using the open‐source TrackNTrace toolbox.^[^ ^62^ ^]^ The circulating nanosheets were then counted in 10‐s intervals, across a total of 6900 frames. The trajectories derived from the tracking algorithm provided additional functional information, such as flow velocity and direction at a given frame rate. By superimposing the particle count/flow velocity/direction at each pixel, the structural/flow velocity/direction maps of brain vasculature were rendered accordingly. Similar imaging conditions were used for visualization of NS‐loaded macrophages. Image analysis for macrophages tracking was performed using background‐subtracted images in TrackMate (v7.10.2), ImageJ (1.54).

### Statistical Analysis

Pre‐processing of XRD and PL data was performed using normalization to a [0–1] scale. PL‐QY data were reported as mean ± SD, measured across an excitation range of 580–635 nm in 5 nm steps (N = 1). Fluorescence lifetime data were also presented as mean ± SD, based on 60 measurements acquired at 1‐s intervals (N = 1). In vivo bioimaging experiments were conducted using N = 4 mice. Image post‐processing included denoising and localization of individual moving particles. Cell viability and LDH release assays were reported as mean ± SD (N = 3). Statistical analyses were performed using OriginPro 2025, GraphPad Prism 10, and ImageJ (version 1.54).

## Conflict of Interest

The authors declare no conflict of interest.

## Supporting information



Supporting Information

Supplemental Video 1

Supplemental Video 2

## Data Availability

The data that support the findings of this study are available from the corresponding author upon reasonable request.
