# Supplementary material for: Unlocking NIR‐II Photoluminescence in 2D Copper Tetrasilicate Nanosheets through Flame Spray Synthesis
Source: Adv Mater. 2025 Jun 13;37(40):2503159. doi: 10.1002/adma.202503159 (PMC12510275; doi:10.1002/adma.202503159)
Supplement: Supplementary file 1 — Supporting Information [file ADMA-37-2503159-s002.pdf]

ADVANCED MATERIALS

Supporting Information

for *Adv. Mater.*, DOI 10.1002/adma.202503159

Unlocking NIR-II Photoluminescence in 2D Copper Tetrasilicate Nanosheets through Flame Spray Synthesis

Robert Nißler, Quanyu Zhou, Björn Hill, Sabrina L.J. Thomä, Lukas R.H. Gerken, Aurelio Borzi, Kevin Roost, Benjamin Mächler, Xosé Luís Deán-Ben, Antonia Neels, Sebastian Kruss, Daniel Razansky and Inge K. Herrmann**

Supplementary Information

Unlocking NIR-II Photoluminescence in 2D Copper Tetrasilicate Nanosheets Through Flame Spray Pyrolysis

Robert Nißler[1,2,3], Quanyu Zhou[4,5], Björn Hill[6], Sabrina L.J. Thomä[7], Lukas R.H. Gerken[1,2], Aurelio Borzi[7], Kevin Roost[1], Benjamin Mächler[1], Xosé Luís Deán-Ben[4,5], Antonia Neels[7], Sebastian Kruss[6,8], Daniel Razansky[4,5], Inge K. Herrmann[1,2,3]**

[1] Nanoparticle Systems Engineering Laboratory, Institute of Energy and Process Engineering (IEPE), Department of Mechanical and Process Engineering (D-MAVT), ETH Zurich, Sonneggstrasse 3, 8092 Zurich, Switzerland.

[2] Particles-Biology Interactions, Department of Materials Meet Life, Swiss Federal Laboratories for Materials Science and Technology (Empa), Lerchenfeldstrasse 5, 9014 St. Gallen, Switzerland.

[3] The Ingenuity Lab, University Hospital Balgrist, University of Zurich, Balgrist Campus, Forchstrasse 340, 8008 Zurich, Switzerland.

[4] Institute for Biomedical Engineering and Institute of Pharmacology and Toxicology, Faculty of Medicine, University of Zurich, Switzerland

[5] Institute for Biomedical Engineering, Department of Information Technology and Electrical Engineering, ETH Zurich, Switzerland

[6] Department of Chemistry, Bochum University, Bochum, Germany

[7] Center for X-ray Analytics, Swiss Federal Laboratories for Materials Science and Technology (Empa), Dübendorf, Switzerland

[8] Fraunhofer Institute for Microelectronic Circuits and Systems, 47057 Duisburg, Germany.

* Corresponding authors: rnissler@ethz.ch, ingeh@ethz.ch

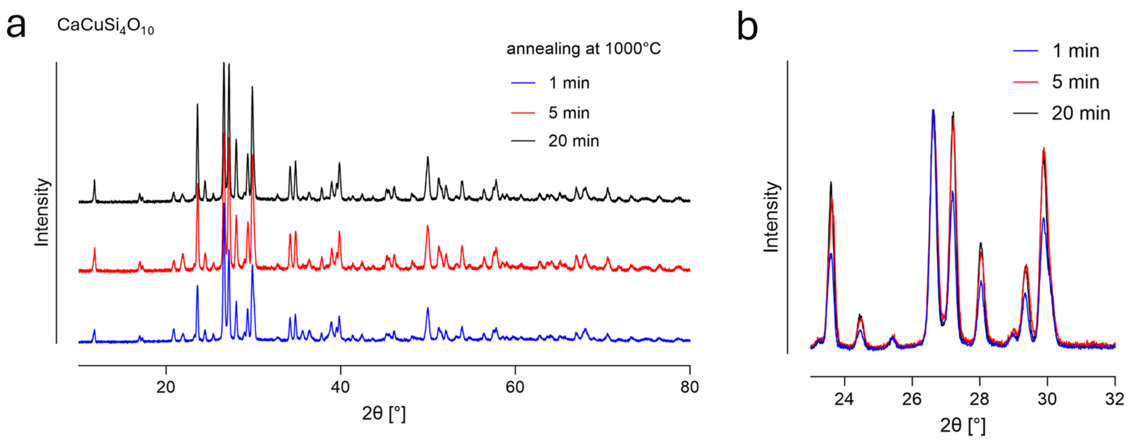

Supplementary Figure S1: a) XRD profiles of FSP-NPs with the nominal chemical composition of $CaCuSi_4O_{10}$, sintered at 1000°C for varying annealing times. b) Zoom into the region between 23-32° 2θ and normalized to the (202) reflex of the $CaCuSi_4O_{10}$ material.

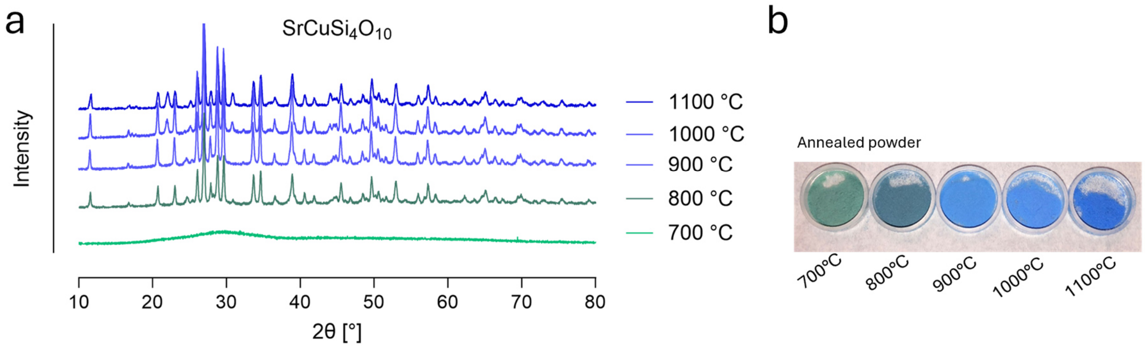

Supplementary Figure S2: a) XRD profiles of FSP-NPs with the nominal chemical composition of $SrCuSi_4O_{10}$, sintered at different temperatures for 10 min. b) Picture of annealed NPs.

Table S1: Surface area of FSP-synthesized nanomaterial before and after annealing measured via Brunauer-Emmett-Teller (BET) analysis. Commercial material purchased from Kremer Pigmente.

Copper tetrasilicate (m^2/g)	$CaCuSi_4O_{10}$	$BaCuSi_4O_{10}$	$SrCuSi_4O_{10}$
commercial	1.5 ± 0.3	1.3 ± 0.1	-
FSP	123.5 ± 0.4	94 ± 0.4	102.5 ± 0.8
Annealed 2 h @ 1000°C	2.8 ± 0.3	1.6 ± 0.3	5.0 ± 0.2
Annealed 20 min @ 1000°C	3.0 ± 0.1	8.2 ± 0.1	13.3 ± 0.1

a

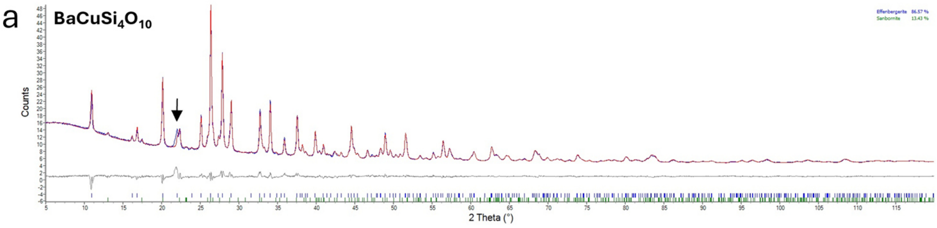

BaCuSi$_4$O$_{10}$ (24h@1000°C)	Main phase = BaCuSi$_4$O$_{10}$	Minor phase = BaSi$_2$O$_5$
Space group	P4/n c c Z ; #130	P c m n ; #62
Cell Volume (Å^3)	893.60	484.7
Lattice parameters (Å)	a = 7.4415<br>c = 16.1351	a = 4.655<br>b= 7.697<br>c= 13.524
Rietveld quantification (%)<br>Rwp = 2.6%	86.6	13.4

b

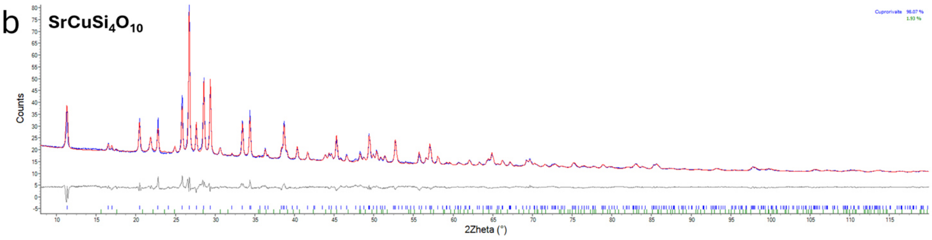

SrCuSi$_4$O$_{10}$ (24h@1000°C)	Main phase = SrCuSi$_4$O$_{10}$	Minor
Space group	P4/n c c Z ; #130	P -4 21 m #113
Cell Volume (Å^3)	847.8	322.6
Lattice parameters (Å)	a = 7.3738<br>c = 15.5894	a = 7.997<br>c = 5.044
Rietveld quantification (%)<br>Rwp = 2.4%	98.0	2.0

c

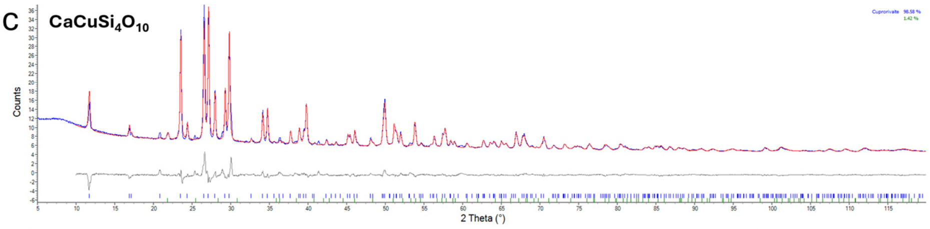

CaCuSi$_4$O$_{10}$ (24h@1000°C)	Main phase = CaCuSi$_4$O$_{10}$	Minor phase = SiO$_2$
Space group	P4/n c c Z ; #130	P41 21 2 ; #92
Cell Volume (Å^3)	807.60 (7)	174.9 (5)
Lattice parameters (Å)	a = 7.3048<br>c = 15.1340	a = 4.944<br>c = 7.15
Rietveld quantification (%)<br>Rwp = 3.6%	98.6	1.4

Supplementary Figure S3: Rietveld analyses of Group-II-cations copper tetrasilicates. a) BaCuSi$_4$O$_{10}$ annealed for 24h at 1000°C. b) SrCuSi$_4$O$_{10}$ annealed for 24h at 1000°C. c) CaCuSi$_4$O$_{10}$ annealed for 24h at 1000°C. Black arrows indicate the presence of a minor, unidentified phase.

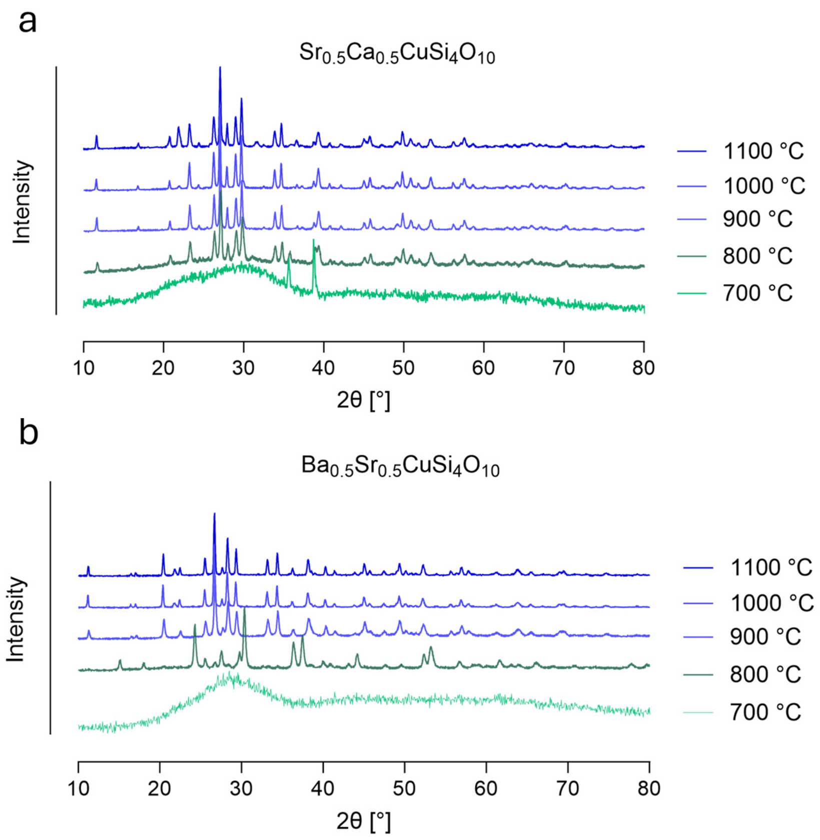

Supplementary Figure S4: XRD profiles of FSP-NPs with the nominal chemical composition of a) $Sr_{0.5}Ca_{0.5}CuSi_4O_{10}$ and b) $Ba_{0.5}Sr_{0.5}CuSi_4O_{10}$ sintered at different temperatures for 10 min.

a

$Ba_{0.5}Sr_{0.5}CuSi_4O_{10}$

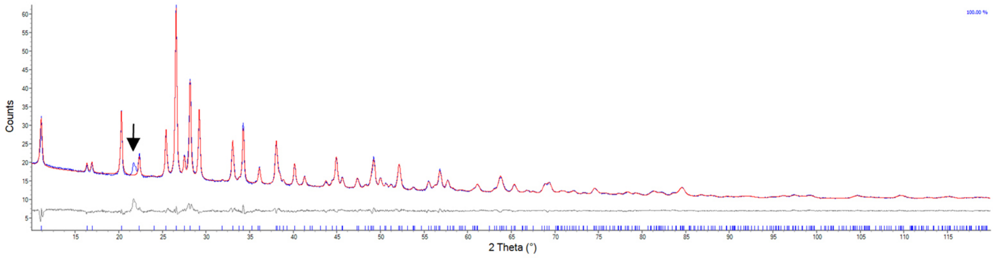

**$Ba_{0.5}Sr_{0.5}CuSi_4O_{10}$ (24h@1000°C)**	**Main**
Space group	P4/n c c Z ; #130
Cell Volume (Å^3)	870.1
Lattice parameters (Å)	a = 7.398<br>c = 15.897

b

$Sr_{0.5}Ca_{0.5}CuSi_4O_{10}$

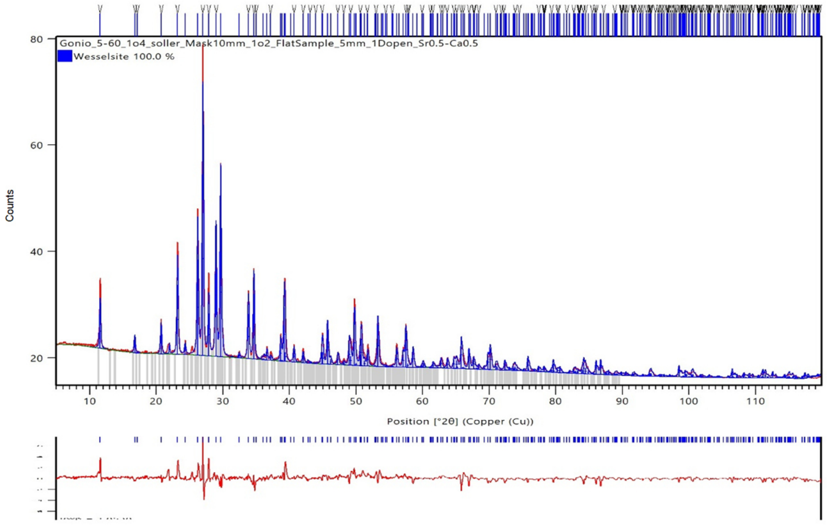

**$Sr_{0.5}Ca_{0.5}CuSi_4O_{10}$ (24h@1000°C)**	**Main**
Space group	P4/n c c Z ; #130
Cell Volume (Å^3)	828.4
Lattice parameters (Å)	a = 7.336<br>c = 15.393

Supplementary Figure S5: Rietveld analyses of mixed group-II-cations copper tetrasilicates. a) $Ba_{0.5}Sr_{0.5}CuSi_4O_{10}$ annealed 24h at 1000°C. b) $Sr_{0.5}Ca_{0.5}CuSi_4O_{10}$ annealed 24h at 1000°C. Black arrows indicate the presence of a minor, unidentified phase.

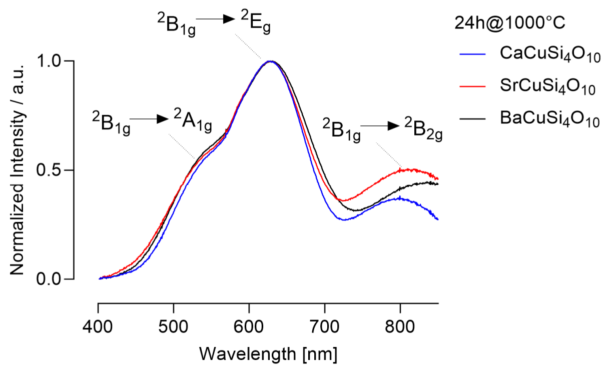

Supplementary Figure S6: UV-Vis-NIR absorption spectra of resynthesized copper tetrasilicates with indicated energy transitions.

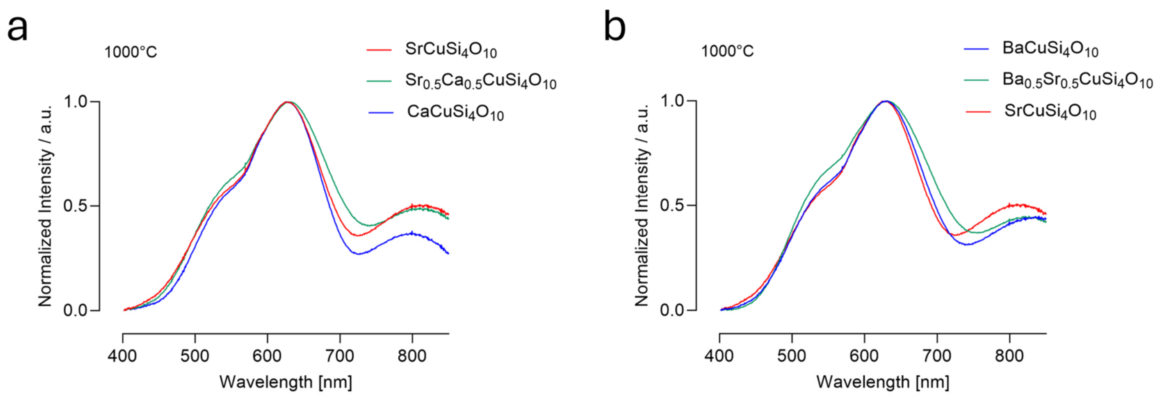

Supplementary Figure S7: UV-Vis-NIR absorption spectra of mixed group-II-cations copper tetrasilicates: $Sr_{0.5}Ca_{0.5}CuSi_4O_{10}$ (a) and $Ba_{0.5}Sr_{0.5}CuSi_4O_{10}$ (b).

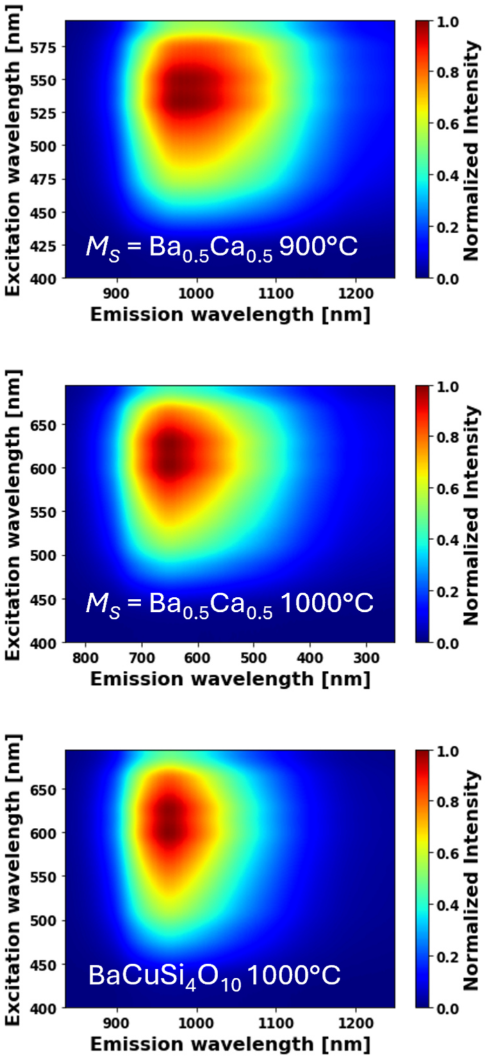

Supplementary Figure S8: 2D excitation-emission PL map for $M_S = Ba_{0.5}Ca_{0.5}$ annealed at different temperatures for 10 min compared to $BaCuSi_4O_{10}$

a

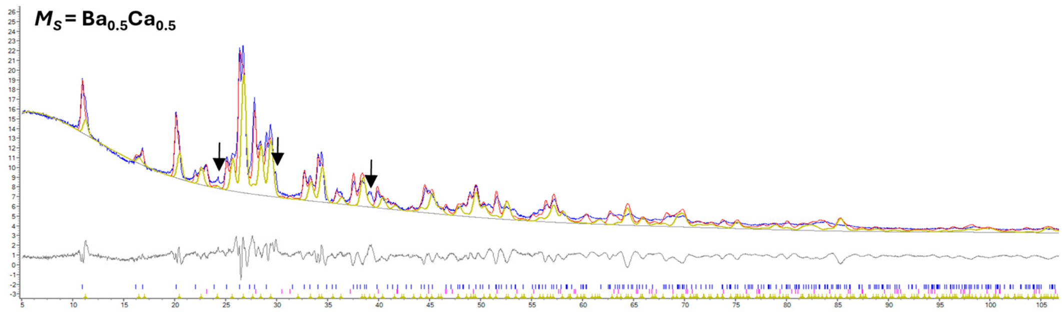

M_S = $Ba_{0.5}Ca_{0.5}$ (10min@900°C)	phase 1 = $BaCuSi_4O_{10}$	phase 2 = $Ba_xCa_{1-x}CuSi_4O_{10}$	phase 3 = SiO_2
Space group	P 4/n c c; #130	C 2/c; #15	P 4_1 2_1 2; #92
lattice parameters (Å)	a = 7.448<br>c = 16.138	a =7.362<br>c = 15.737	a = 4.503<br>c = 7.39
Rietveld quantification (%)	33.7	62.7	3.7
Rwp = 4.77 %			

b

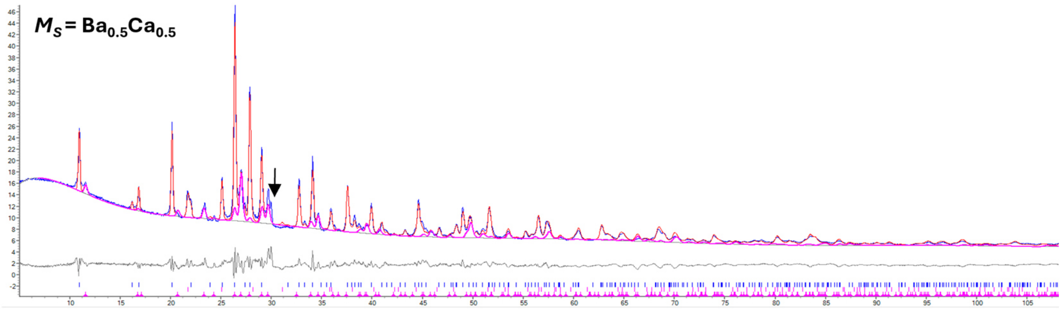

M_S = $Ba_{0.5}Ca_{0.5}$ (10min@1000°C)	phase 1 = $BaCuSi_4O_{10}$	phase 2 = $Ba_xCa_{1-x}CuSi_4O_{10}$	phase 3 = SiO_2
space group	P 4/n c c; #130	P 4/n c c; #130	P 4_1 2_1 2; #92
lattice parameters (Å)	a = 7.4315<br>c = 16.1209	a = 7.3220<br>c = 15.252	a = 5.016<br>c = 7.015
Rietveld quantification (%)	61.6	31.1	7.1
Rwp = 3.58 %			

Supplementary Figure S9: Rietveld analyses of mixed Ba-Ca-cations copper tetrasilicates. a) FSP-based nanomaterial with the group-II-cations synthesis ratio (M_S) M_S = $Ba_{0.5}Ca_{0.5}$ annealed for 10 min at 900°C and in b) for 10 min at 1000°C. Black arrows indicate the presence of a minor, unidentified phase.

a

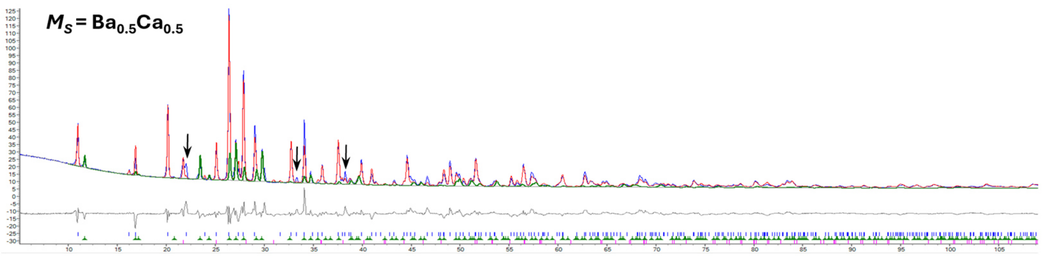

M_S = $Ba_{0.5}Ca_{0.5}$ (24h@1000°C)	phase 1 = $BaCuSi_4O_{10}$	phase 2 = $CaCuSi_4O_{10}$	phase 3 = SiO_2
Space group	P 4/n c c; #130	P 4/n c c; #130	P 4_1 2_1 2; #92
Lattice parameters (Å)	a =7.44<br>c = 16.13	a = 7.30<br>c = 15.18	a = 5.02<br>c = 7.06
Rietveld quantification (%)	57.9	37.5	4.7
Rwp = 7.62 %			

b

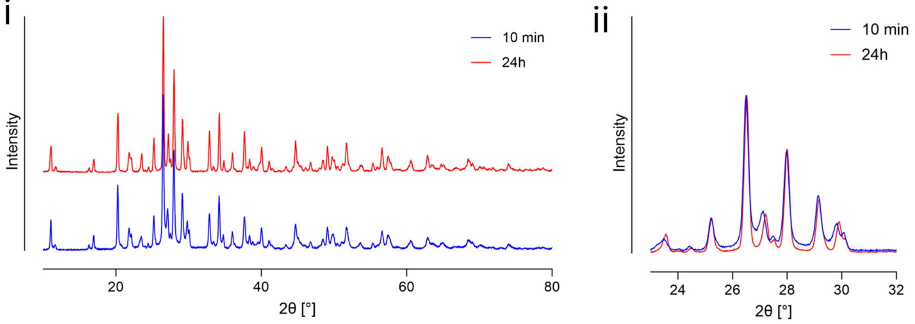

c

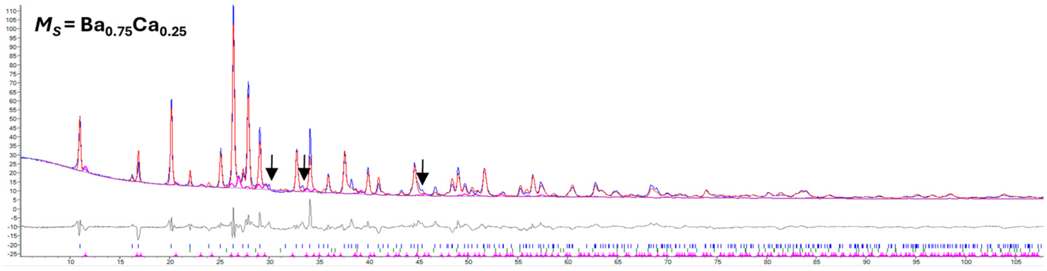

M_S = $Ba_{0.75}Ca_{0.25}$	phase 1 = $BaCuSi_4O_{10}$	phase 2 = $Ba_xCa_{1-x}CuSi_4O_{10}$	phase 3 = SiO_2
Space group	P 4/n c c; #130	P 4/n c c; #130	P 4_1 2_1 2; #92
lattice parameters (Å)	a =7.433<br>c = 16.122	a = 7.345<br>c = 15.345	a = 4.905<br>c = 7.07
Rietveld quantification (%)	85.1	13.4	1.7
Rwp = 7.14 %			

Supplementary Figure S10: Rietveld analyses of mixed group-II-cations copper tetrasilicates. a) M_S = $Ba_{0.5}Ca_{0.5}$ annealed for 24 h at 900°C. b) Comparison of the XRD pattern of M_S = $Ba_{0.5}Ca_{0.5}$ annealed for 10 min and 24 h (i) with zoom-in region (ii) between 23.5-32° 2θ and normalized to the (202) reflex of the copper tetrasilicate material. c) Rietveld analyses of M_S = $Ba_{0.75}Ca_{0.25}$ annealed for 2h at 1000°C. Black arrows indicate the presence of a minor, unidentified phase.

a M_S = $Ba_{0.15}Ca_{0.85}$

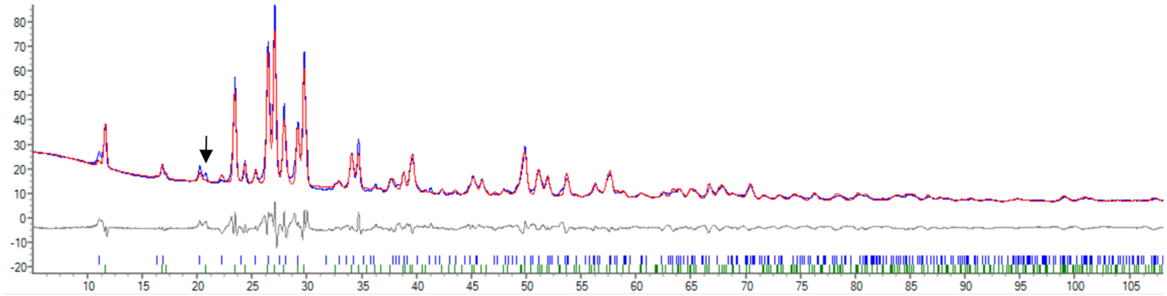

M_S = $Ba_{0.15}Ca_{0.85}$ (10min@1000°C)	phase 1 = $Ba_xCa_{1-x}CuSi_4O_{10}$	phase 2 = $Ba_xCa_{1-x}CuSi_4O_{10}$
Space group	P 4/n c c; #130	P 4/n c c; #130
lattice parameters (Å)	a = 7.3409<br>c = 15.973	a = 7.3104<br>c = 15.184
Rietveld quantification (%)	12.9	86.9
Rwp = 6.08 %		

b M_S = $Ba_{0.25}Ca_{0.75}$

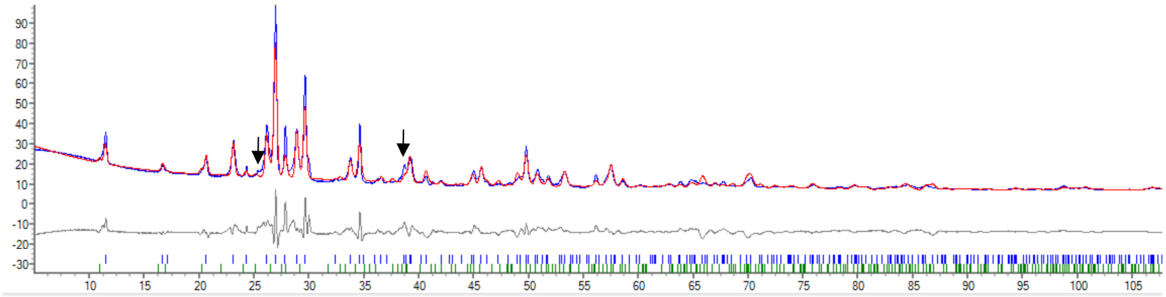

M_S = $Ba_{0.15}Ca_{0.85}$ (10min@1000°C)	phase 1 = $Ba_xCa_{1-x}CuSi_4O_{10}$	phase 2 = $Ba_xCa_{1-x}CuSi_4O_{10}$
Space group	P 4/n c c; #130	P 4/n c c; #130
lattice parameters (Å)	a = 7.3222<br>c = 15.378	a = 7.394<br>c = 16.09
Rietveld quantification (%)	93.7	7.1
Rwp = 8.47 %		

Supplementary Figure S11: Rietveld analyses of near homogeneous Ba-Ca-cation mixed copper tetrasilicates. a) M_S = $Ba_{0.15}Ca_{0.85}$ annealed for 10 min at 1000°C. b) Rietveld analyses of M_S = $Ba_{0.25}Ca_{0.75}$ annealed for 10 min at 1000°C. Black arrows indicate the presence of a minor, unidentified phase. As both samples showed the near exclusive formation of a single $Ba_xCa_{1-x}CuSi_4O_{10}$ phase, this phase is referred as $Ba_{0.15}Ca_{0.85}CuSi_4O_{10}$ (a) and $Ba_{0.25}Ca_{0.75}CuSi_4O_{10}$ (b) respectively.

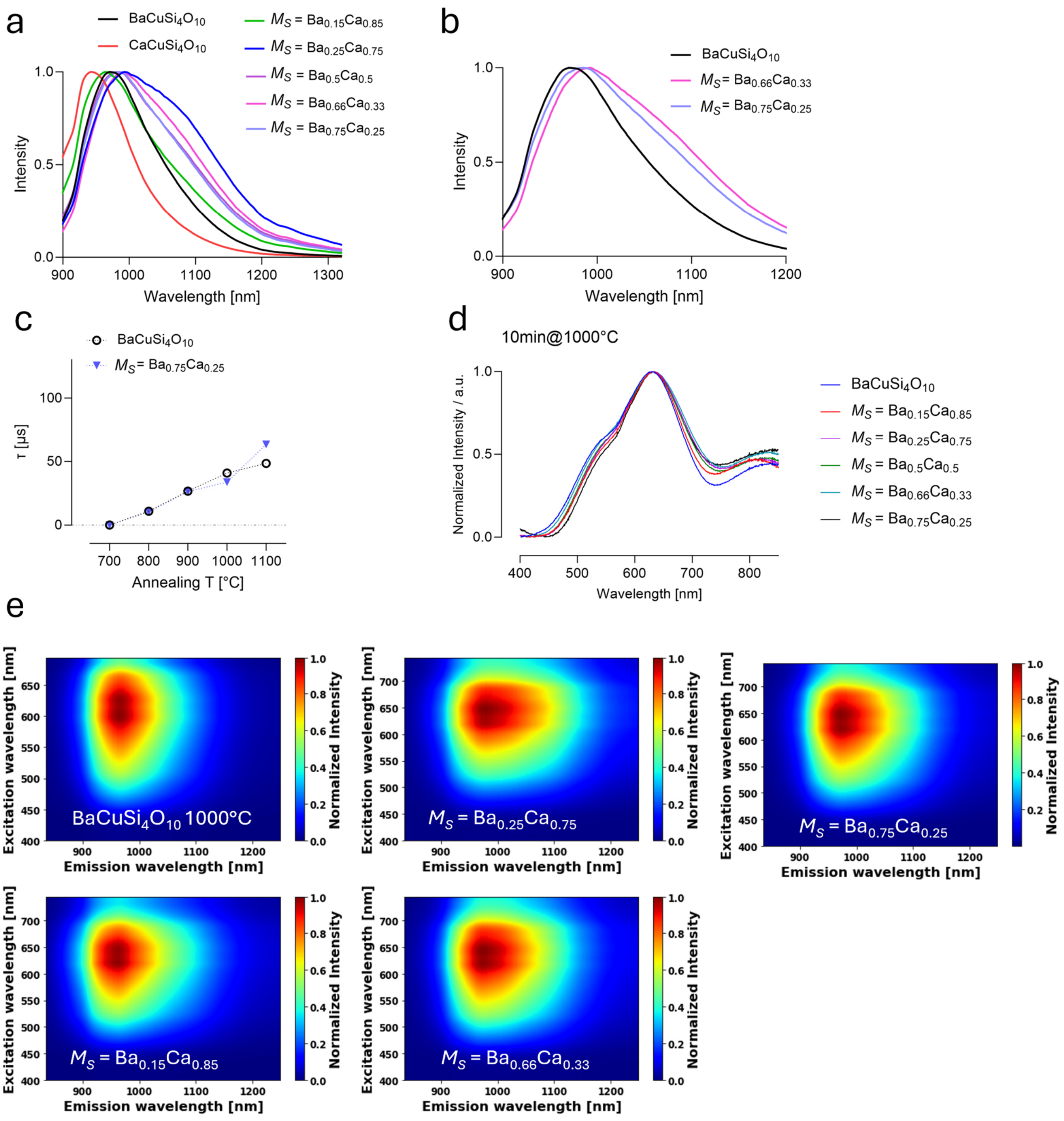

Supplementary Figure S12: Optical properties of Ba-Ca intermixed copper tetrasilicates. a) PL spectra of $CaCuSi_4O_{10}$ and $BaCuSi_4O_{10}$, as well as of different $M_S = Ba_xCa_x$ samples. b) PL emission of $BaCuSi_4O_{10}$ compared to the minorly doped phases, resulting from $M_S = Ba_{0.75}Ca_{0.25}$ and $M_S = Ba_{0.66}Ca_{0.33}$ annealed for 2h at 1000°C. c) Fluorescence lifetimes of $BaCuSi_4O_{10}$ and $M_S = Ba_{0.75}Ca_{0.25}$ annealed at different temperatures. d) Normalized UV-Vis-NIR absorption spectra of Ba-Ca intermixed copper tetrasilicates. e) 2D excitation-emission PL map of different Ba-Ca intermixed copper tetrasilicates.

a M_S = $Ba_{0.5}Ca_{0.5}$ (24h@1000°C)

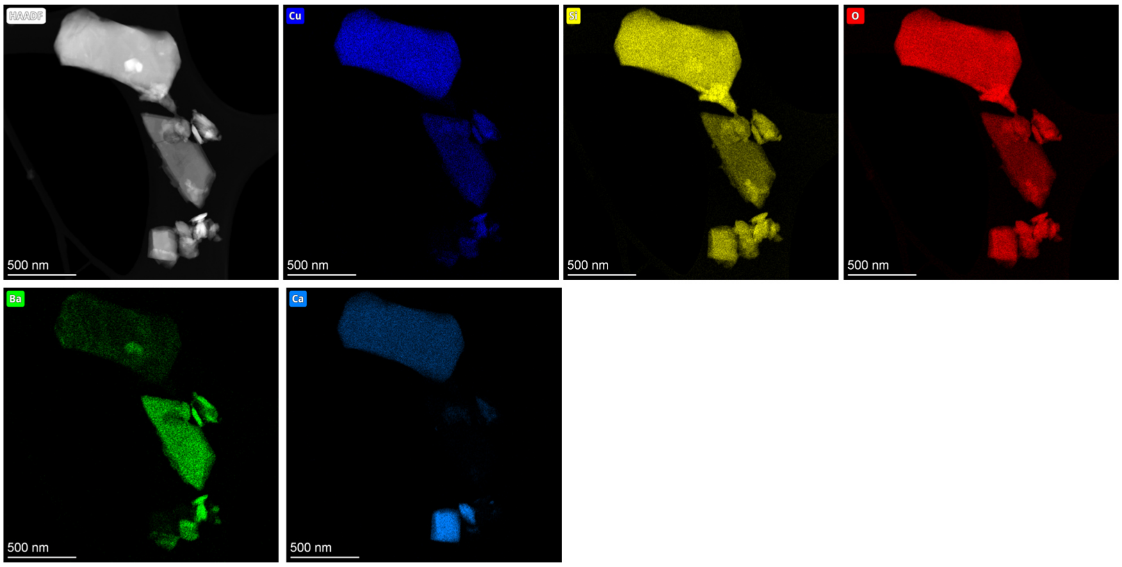

b M_S = $Ba_{0.5}Ca_{0.5}$ (24h@1000°C)

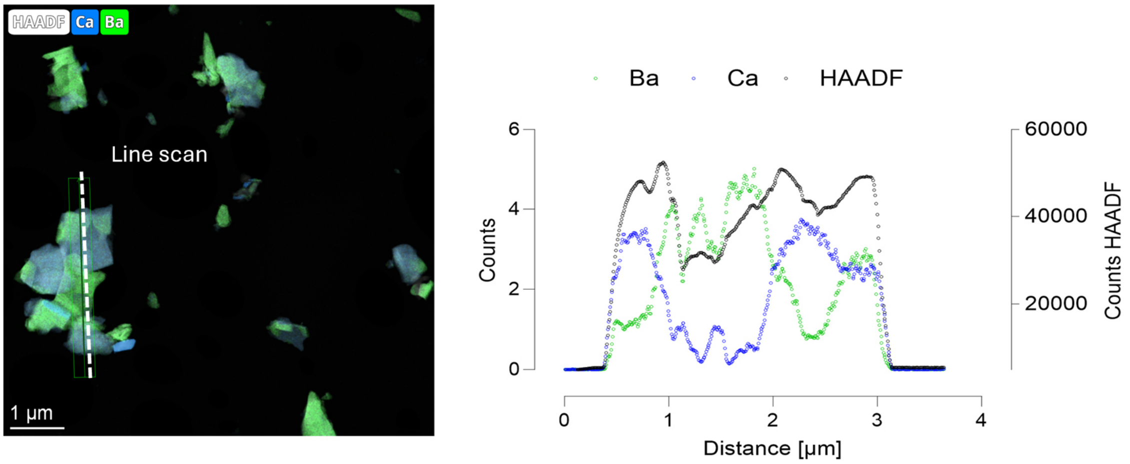

Supplementary Figure S13: STEM analysis of single M_S = $Ba_{0.5}Ca_{0.5}$ nanosheets. a) HAADF image and EDX-based elemental maps show strong segregation of Ba- and Ca-containing copper tetrasilicate phases. b) Overview and line scan of multiple NS indicating a inhomogeneous alkaline earth metal distribution, however with local intermixing.

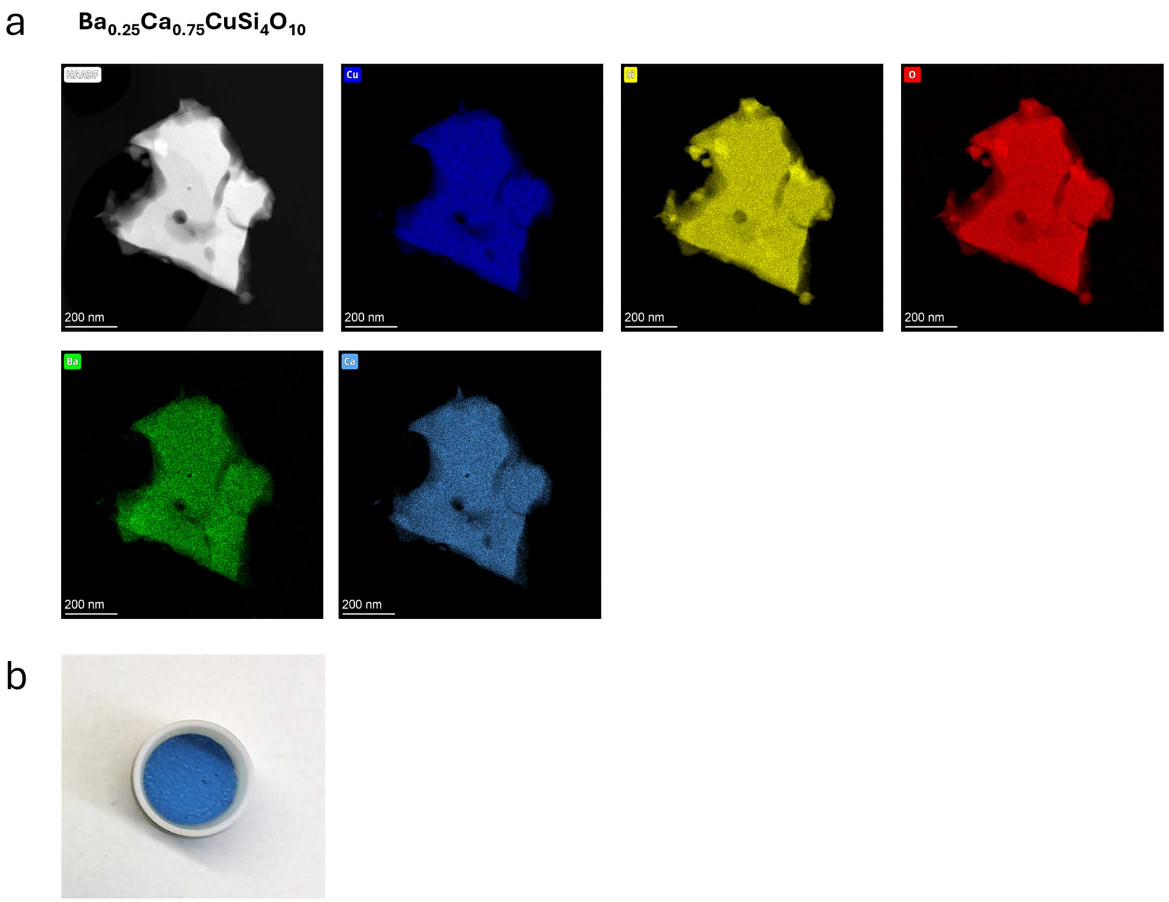

Supplementary Figure S14: STEM analysis of a single $M_S = Ba_{0.25}Ca_{0.75}$ nanosheet. a) HAADF image and corresponding EDX-based elemental maps (full elemental mapping of Figure 3i). b) Photograph of bulk $Ba_{0.25}Ca_{0.75}CuSi_4O_{10}$ material.

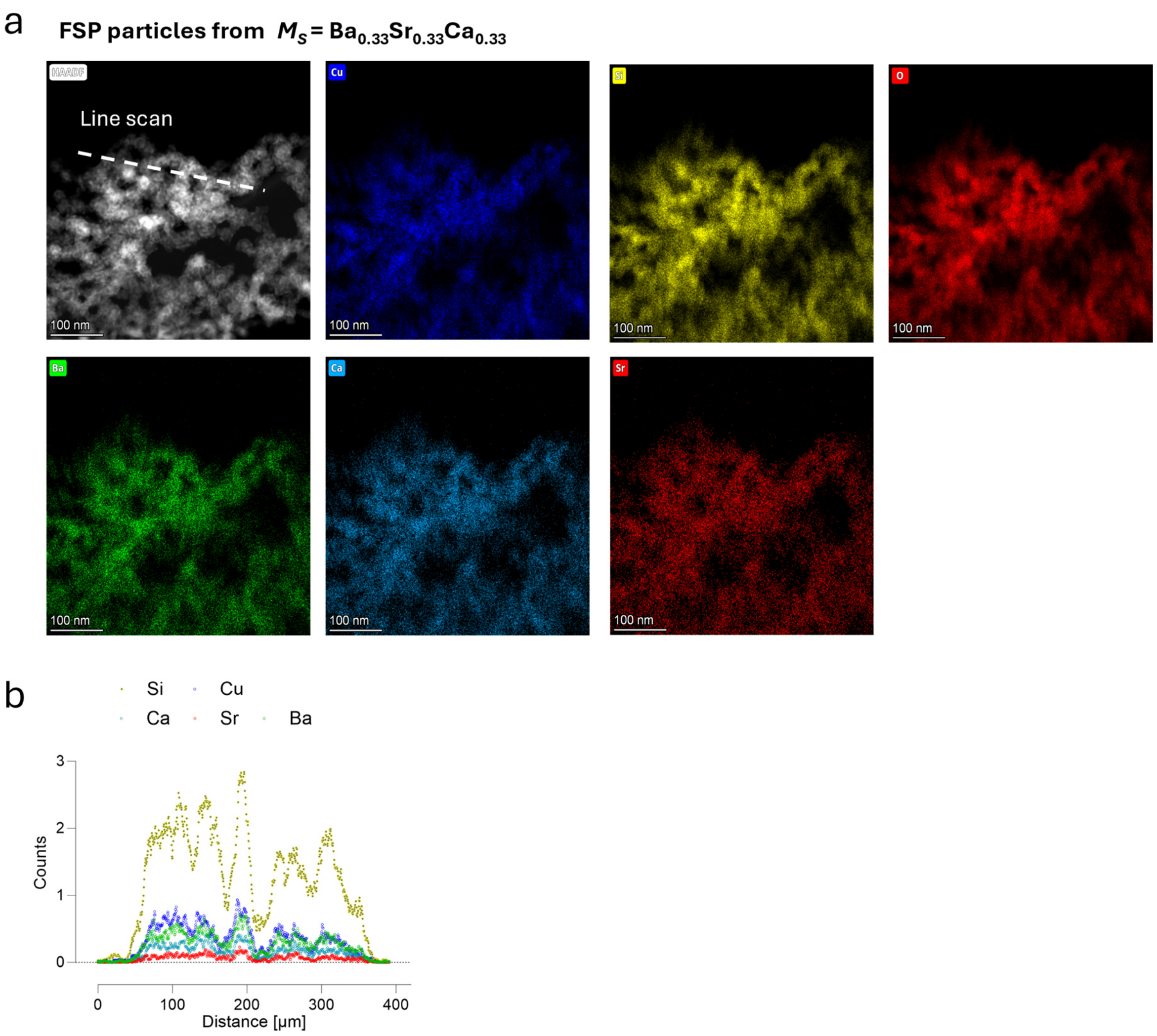

Supplementary Figure S15: STEM analysis of multiple primary FSP-derived nanoparticles with the alkaline earth metal synthesis ratio of $M_S = Ba_{0.33}Sr_{0.33}Ca_{0.33}$. a) HAADF image and corresponding EDX-based elemental maps. b) Corresponding line scan shows a homogeneous distribution of contained elements.

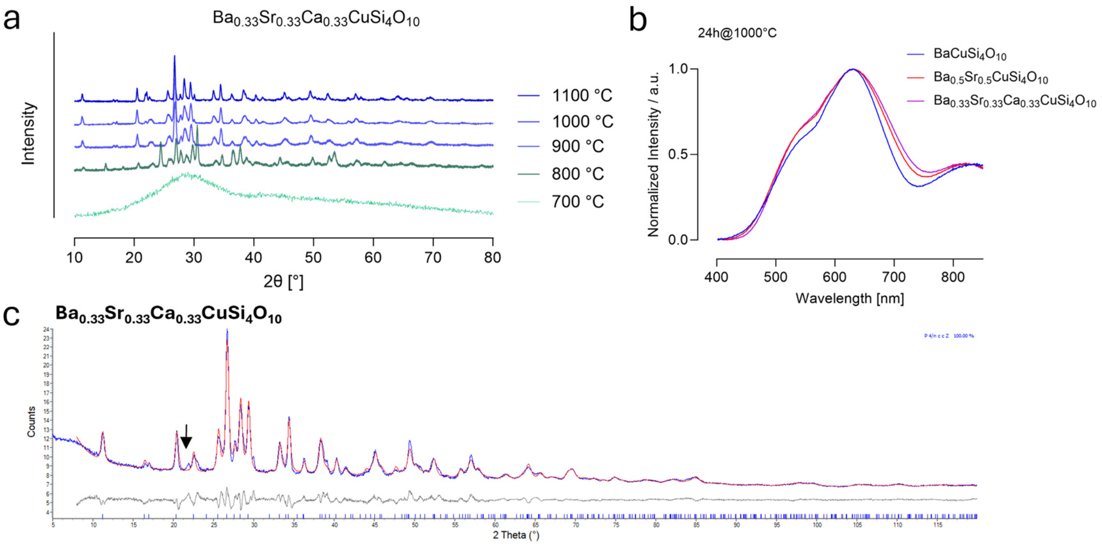

$Ba_{0.33}Sr_{0.33}Ca_{0.33}CuSi_4O_{10}$ (24h@1000°C)	Main
Space group	P4/n c c Z ; #130
Cell Volume (Å^3)	862.1
Lattice parameters (Å)	a = 7.387<br>c = 15.83

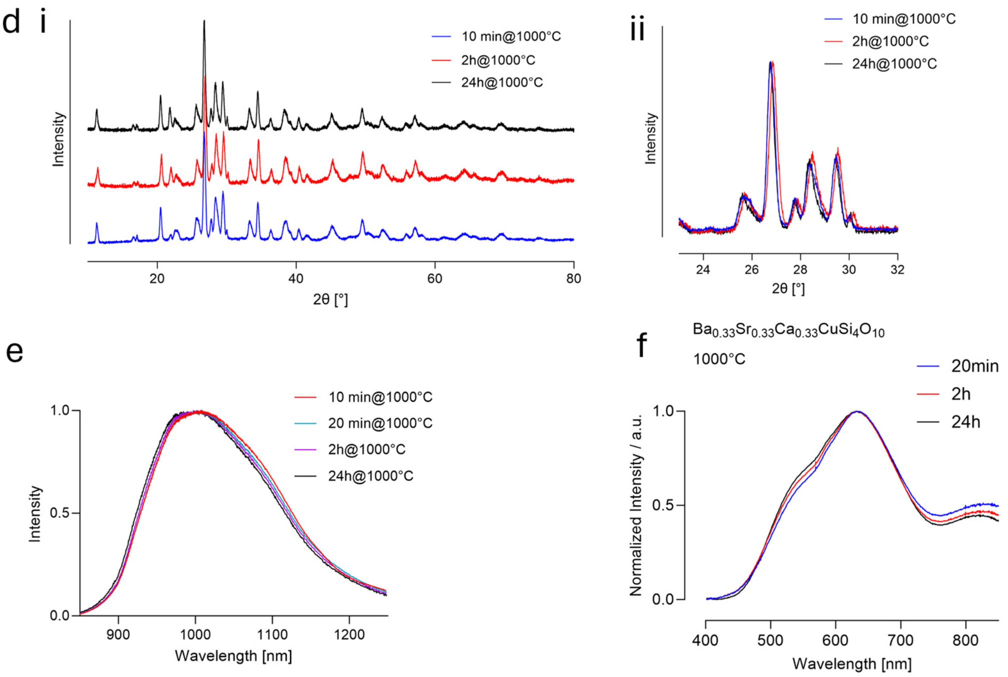

Supplementary Figure S16: Characterization of $Ba_{0.33}Sr_{0.33}Ca_{0.33}CuSi_4O_{10}$. a) XRD profiles of FSP-NPs with the nominal chemical composition $Ba_{0.33}Sr_{0.33}Ca_{0.33}CuSi_4O_{10}$ sintered at different temperatures for 10 min. b) Comparison of UV-Vis-NIR absorption spectra. c) Rietveld analyses. Black arrows indicate the presence of a minor, unidentified phase. d) XRD profiles of material sintered for different timepoints (i) with zoom-in region (ii) between 23.5-32° 2θ and normalized to the (202) reflex of the copper tetrasilicate material. No significant changes and phase separations were observed. e) PL emission and f) UV-Vis-NIR absorption spectra of $Ba_{0.33}Sr_{0.33}Ca_{0.33}CuSi_4O_{10}$ shows no significant changes with prolonged annealing.

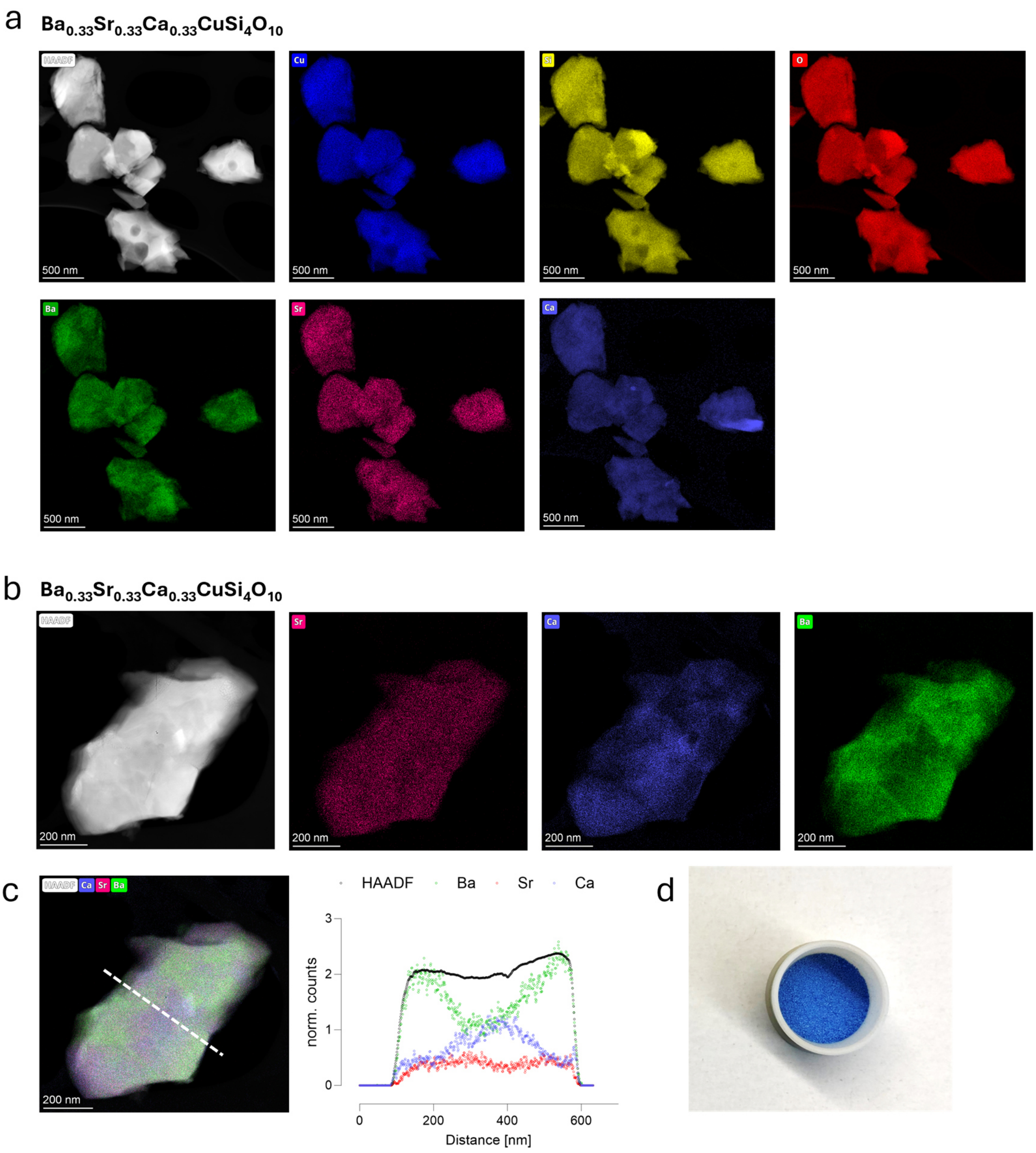

Supplementary Figure S17: STEM analysis of single $Ba_{0.33}Sr_{0.33}Ca_{0.33}CuSi_4O_{10}$ nanosheets. a) Typical HAADF image and EDX-based elemental maps show for most analyzed NS near homogeneous elemental distribution. b) Co-localization between the intermixed alkaline earth metals can vary within a single NS, shown in the corresponding EDX-based elemental maps. c) Overlay and line scan of maps shown in (b). Sr was found homogeneously distributed within the NS, whereas local Ba and Ca levels could reciprocal vary. d) Photograph of bulk $Ba_{0.33}Sr_{0.33}Ca_{0.33}CuSi_4O_{10}$ material.

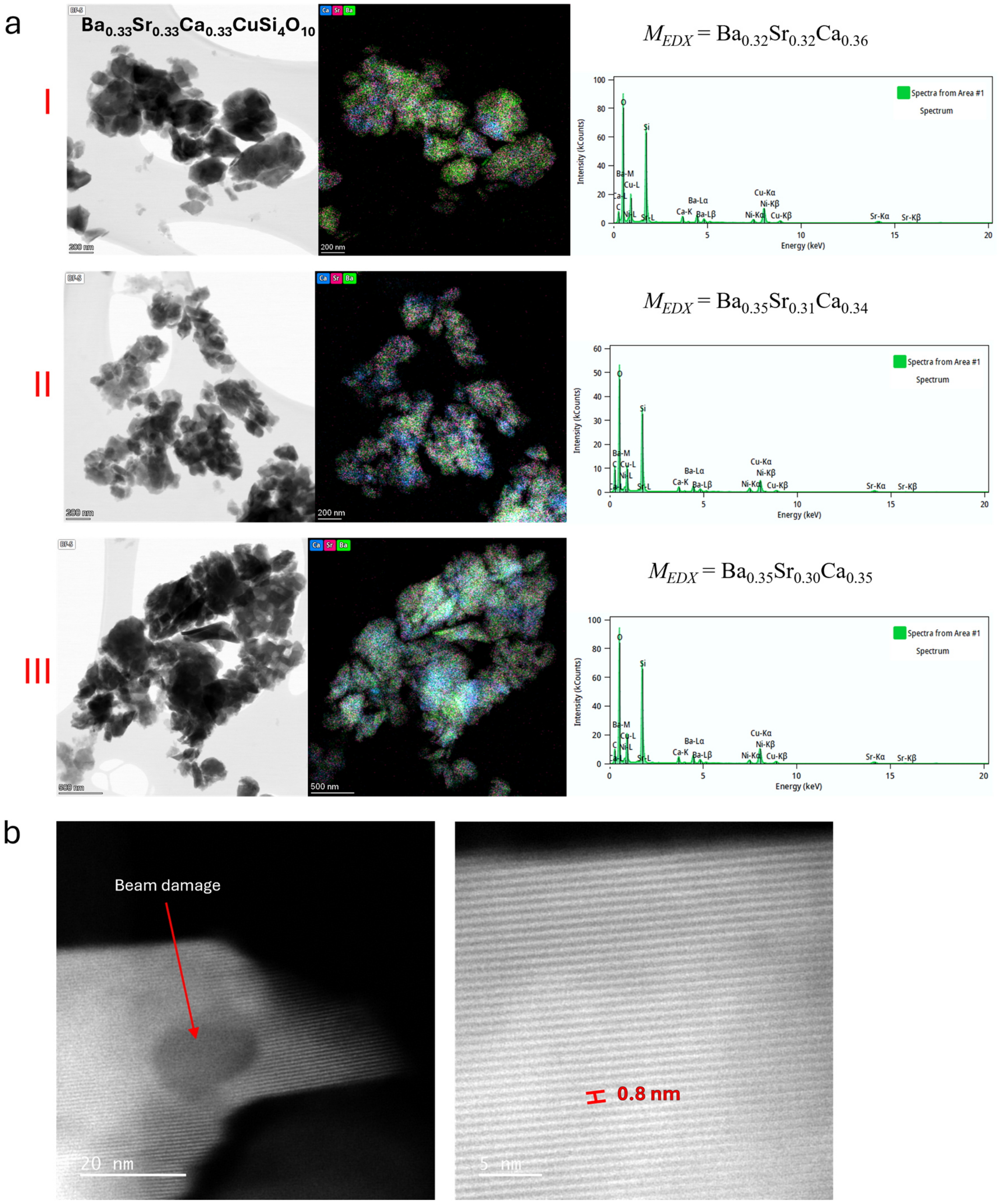

Supplementary Figure S18: Reproducibility analysis of $Ba_{0.33}Sr_{0.33}Ca_{0.33}CuSi_4O_{10}$ nanosheet synthesis. a) Three independent synthesis runs (I-III) are performed, followed by 2h annealing of FSP-particles at 1000°C. STEM analysis of multiple $Ba_{0.33}Sr_{0.33}Ca_{0.33}CuSi_4O_{10}$ nanosheets: Dark field image (left); overlayed EDX-based elemental maps of Ba, Sr and Ca (middle); corresponding EDX-spectra (right). $M_{EDX} = Ba_{0.35\pm0.01}\,Sr_{0.31\pm0.01}\,Ca_{0.34\pm0.02}$ (mean ± SD, N = 3). b) High-resolution TEM analysis using an aberration-corrected JEOL GrandARM TEM at 200 kV. Degradation of the nanosheet due to beam damage occurred within less than a minute (dark field image, right), whereas a lattice spacing of approximately 0.8 nm was clearly visible, as expected for the *M-M* distance in the 2D NS (dark field image, left).

FSP particles from $M_S = Ba_{0.25}Sr_{0.25}Ca_{0.25}Mg_{0.25}$

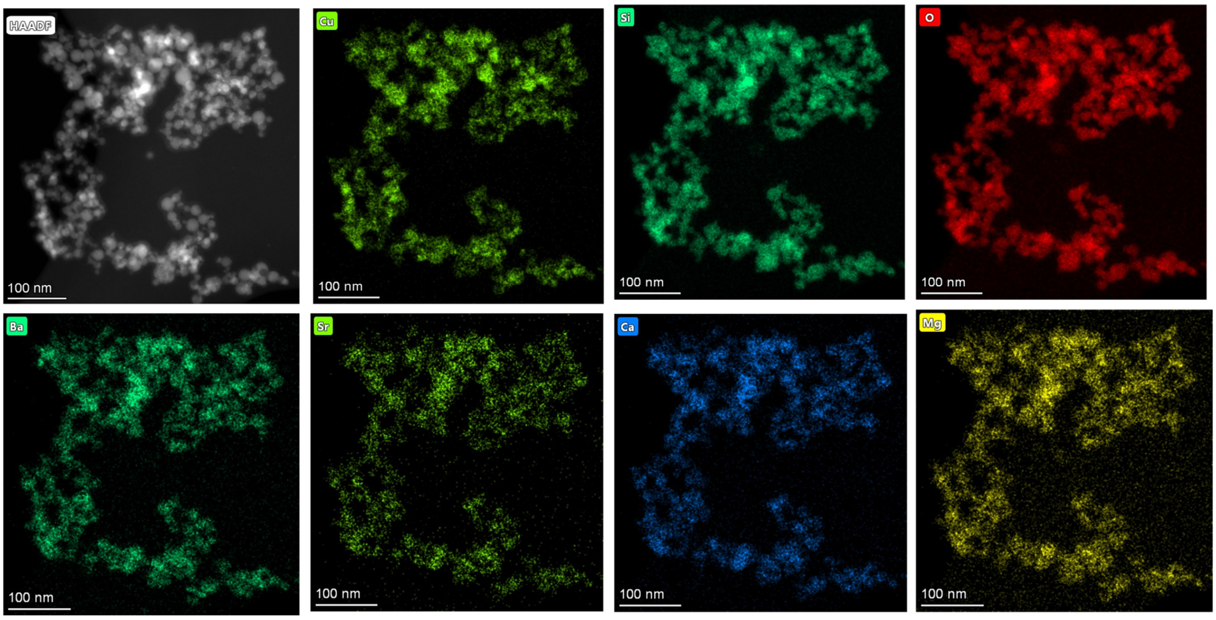

Supplementary Figure S19: STEM analysis of multiple primary FSP-derived nanoparticles with the alkaline earth metal synthesis ratio of $M_S = Ba_{0.25}Sr_{0.25}Ca_{0.25}Mg_{0.25}$ shown as HAADF image and corresponding EDX-based elemental maps. A strong co-localization of all elements is visible, including Mg.

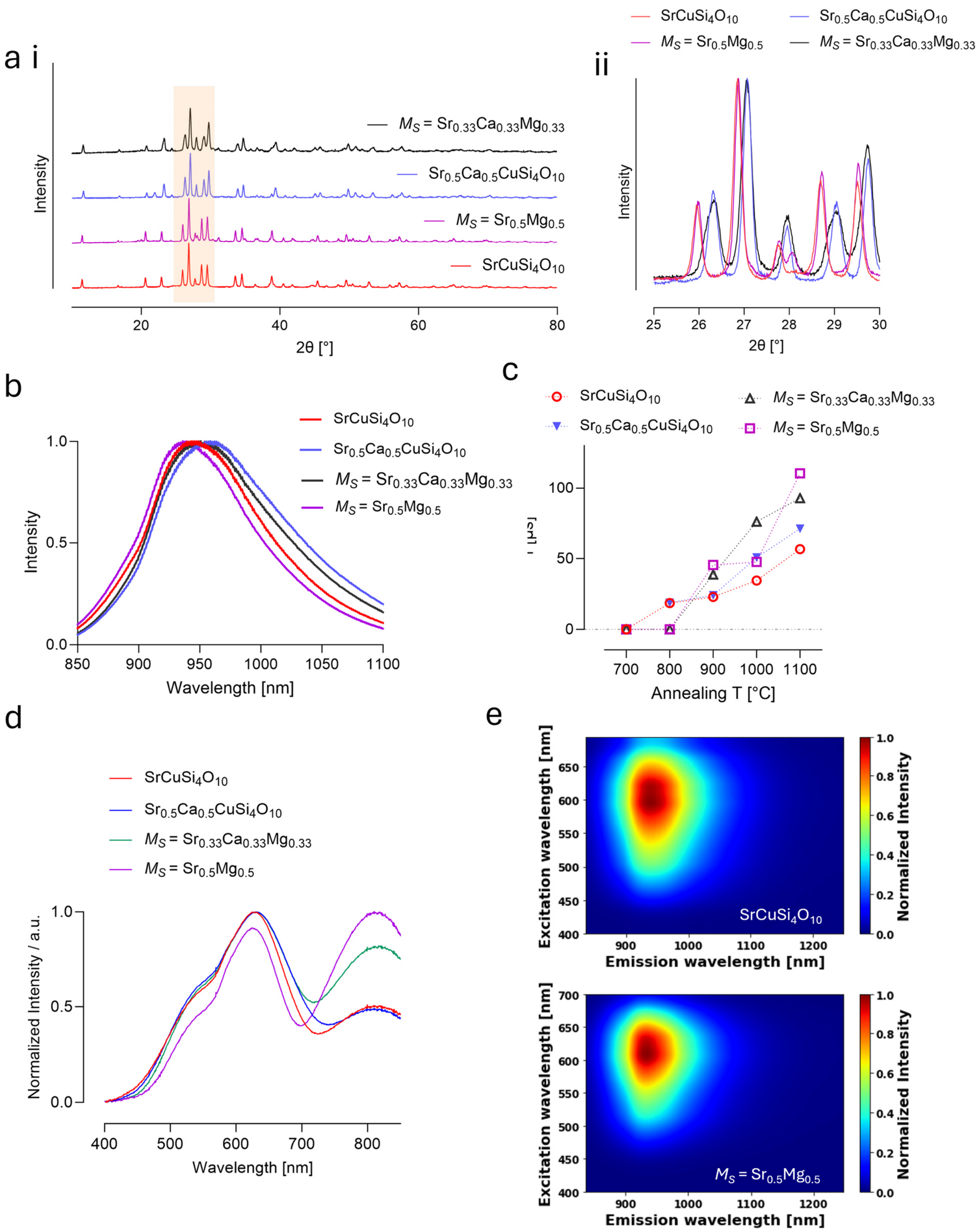

Supplementary Figure S20: Characterization of Mg-doped $SrCuSi_4O_{10}$ and $Sr_{0.5}Ca_{0.5}CuSi_4O_{10}$. a) XRD profiles of different copper tetrasilicate materials (i) with zoom-in region (ii) between 23.5-32° 2θ and normalized to the (202) reflex of the copper tetrasilicate material. No significant shift in reflex position is visible after Mg-doping. b) PL emission spectra. c) Fluorescence lifetime values depending on the annealing temperature. d) UV-Vis-NIR absorption spectra show a enhanced B_{1g}-B_{2g} transition. e) 2D excitation-emission PL map for Mg-doped $SrCuSi_4O_{10}$ compared to undoped $SrCuSi_4O_{10}$.

a **M_S = $Sr_{0.5}Mg_{0.5}$**

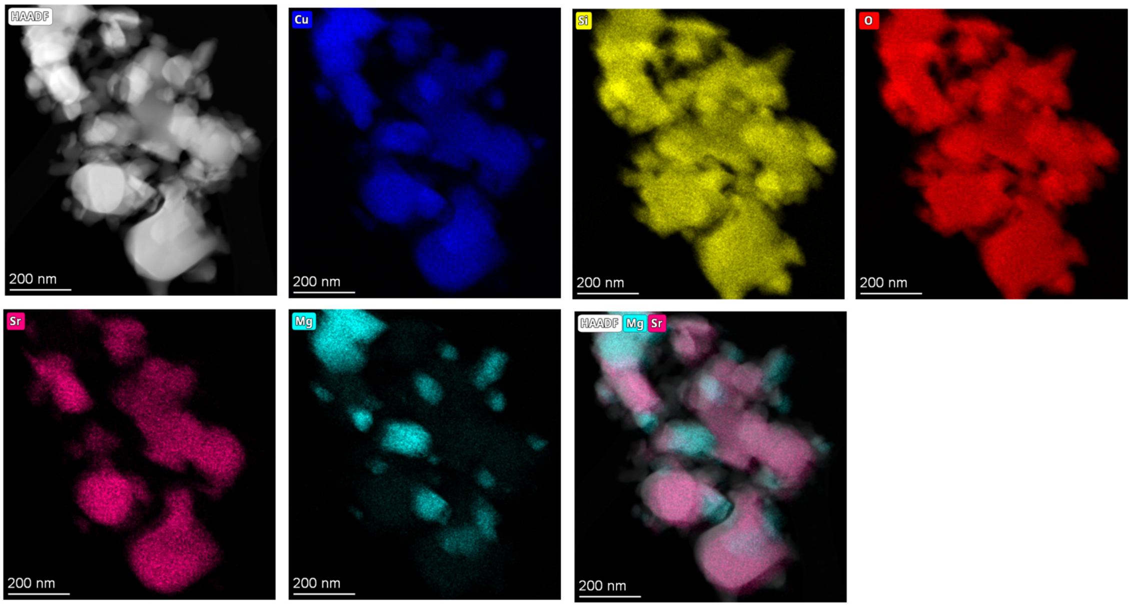

b

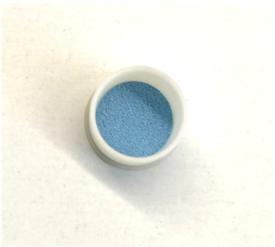

c

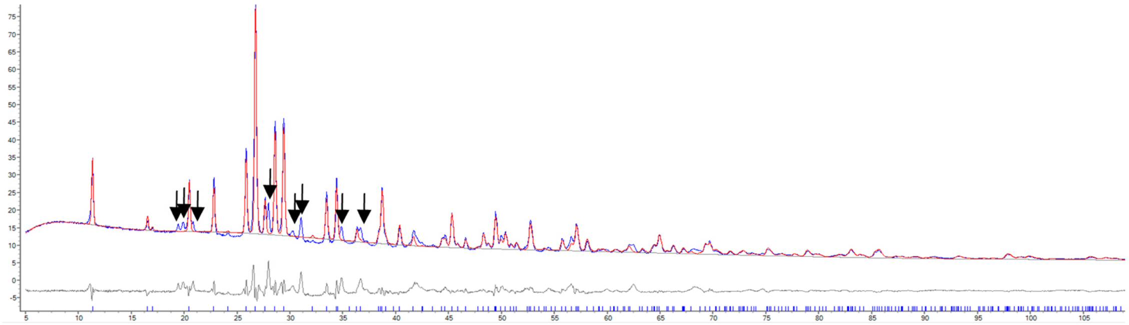

**M_S = $Sr_{0.5}Mg_{0.5}$ (2h@1000°C)**	**phase 1 = $SrCuSi_4O_{10}$**
Space group	P 4/n c c; #130
Lattice parameters (Å)	a = 7.3722<br>c = 15.583
**Rwp = 5.61 %**	

Supplementary Figure S21: Characterization of Mg-doped $SrCuSi_4O_{10}$. a) STEM analysis of nanosheets shown as HAADF image and EDX-based elemental maps. Strong segregation of Mg-containing phases becomes visible on single NS level. b) Photograph of bulk M_S = $Sr_{0.5}Mg_{0.5}$ material. c) Rietveld analyses reveal no significant shift in lattice parameters compared to undoped $SrCuSi_4O_{10}$. Black arrows indicate the presence of a minor, unidentified phase, most likely containing Mg.

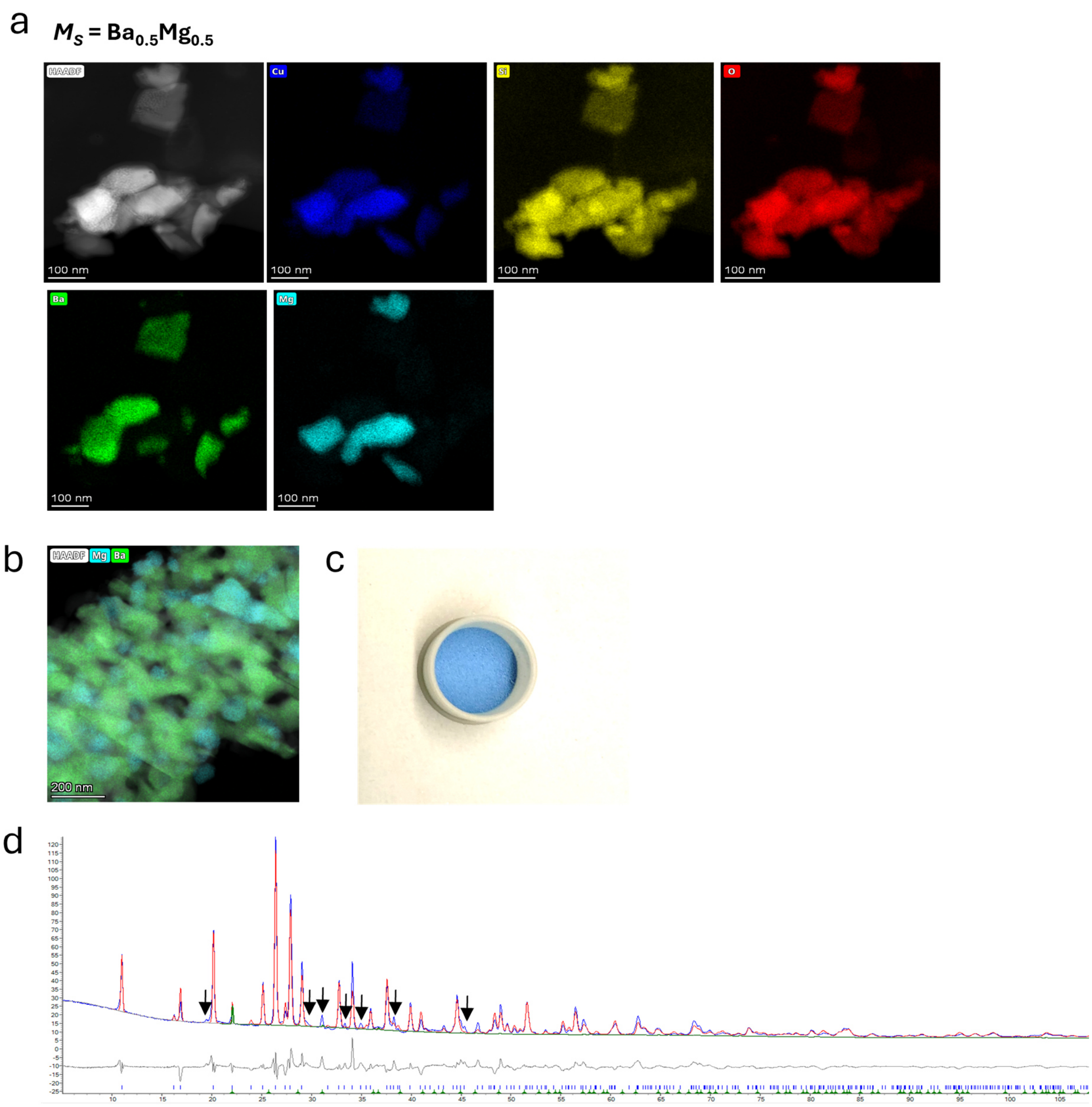

M_S = $Ba_{0.5}Mg_{0.5}$	phase 1 = $BaCuSi_4O_{10}$	phase 2 = SiO_2
Space group	P 4/n c c; #130	P 4_1 2_1 2; #92
Lattice parameters (Å)	a =7.44 0<br>c = 16.134	a = 5.015<br>c = 7.059
Rietveld quantification (%)	96.9	4.7
Rwp = 7.73 %		

Supplementary Figure S22: Characterization of Mg-doped $BaCuSi_4O_{10}$. a) STEM analysis of single nanosheets shown as HAADF image and EDX-based elemental maps. Strong segregation of Mg-containing phases is visible on single NS level and (b) within bulk material. c) Photograph of bulk M_S = $Ba_{0.5}Mg_{0.5}$ material. d) Rietveld analyses reveal no significant shift in lattice parameters after slight Mg-doping. Black arrows indicate the presence of a minor, unidentified phase, most likely containing Mg.

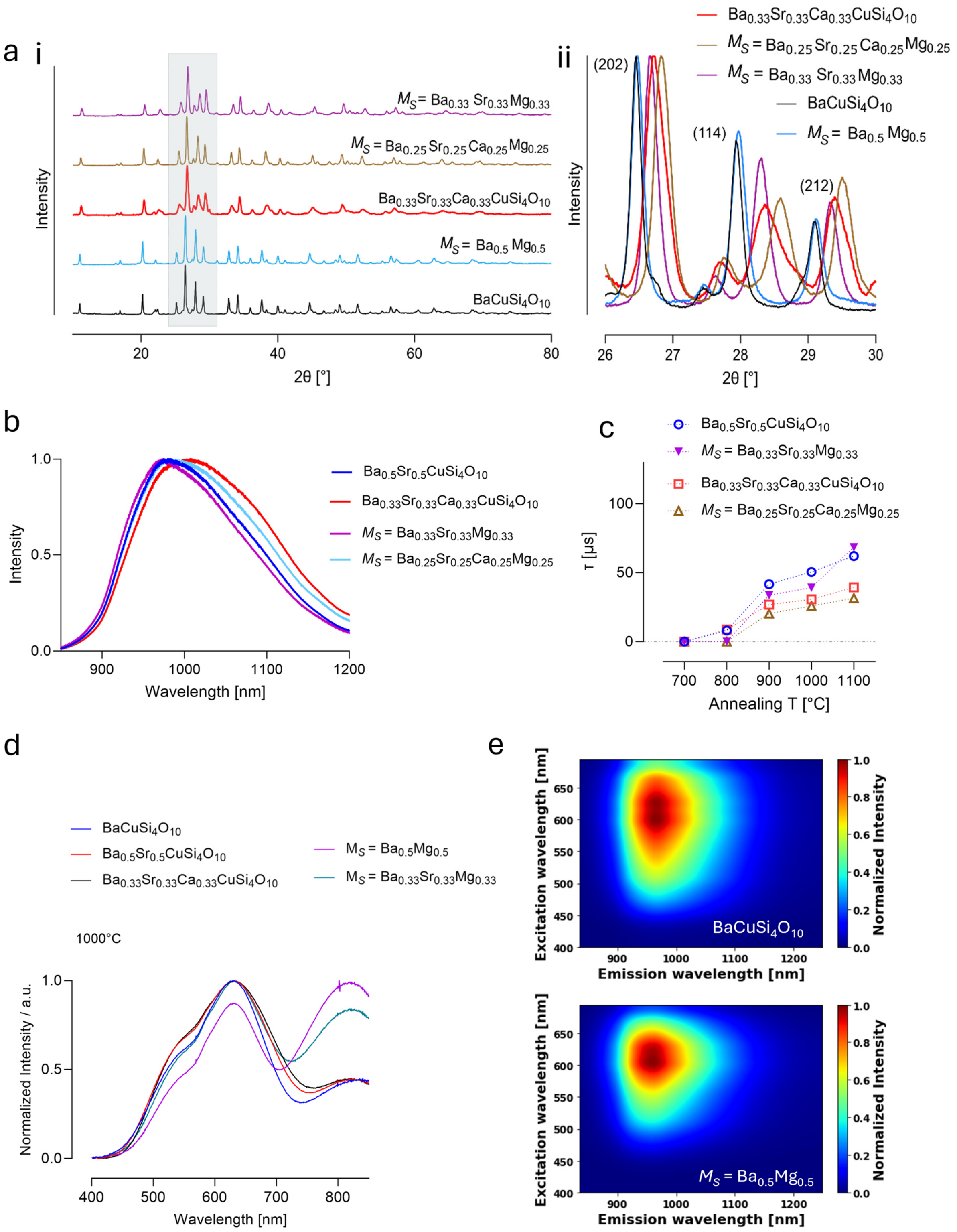

Supplementary Figure S23: Characterization of multielement doping of copper tetrasilicates. a) XRD profiles of different copper tetrasilicate materials (i) with zoom-in region (ii) between 23.5-32° 2θ and normalized to the (202) reflex of the copper tetrasilicate material. Slight shift to larger reflex positions was observed for Mg-doped $Ba_{0.33}Sr_{0.33}Ca_{0.33}CuSi_4O_{10}$. b) PL emission spectra. c) Fluorescence lifetime values depending on the annealing temperature. d) UV-Vis-NIR absorption spectra show a B_{1g}-B_{2g} transition after Mg introduction. e) 2D excitation-emission PL map for Mg-doped $BaCuSi_4O_{10}$ compared to undoped $BaCuSi_4O_{10}$.

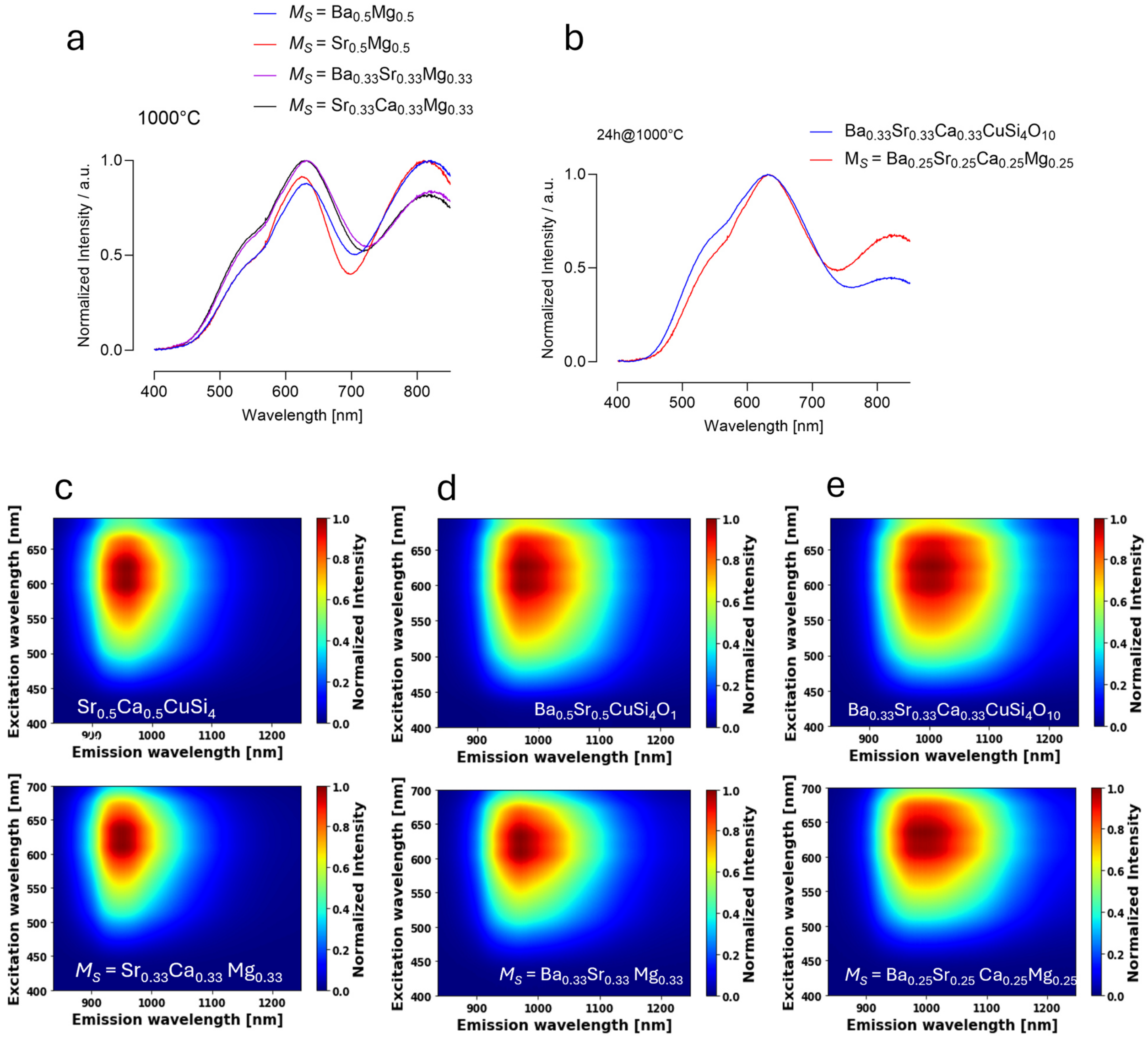

Supplementary Figure S24: Extended optical characterization of multielement doped copper tetrasilicates. a) UV-Vis-NIR absorption spectra of different Mg-doped copper tetrasilicates. b) UV-Vis-NIR absorption spectra of Mg-doped $Ba_{0.33}Sr_{0.33}Ca_{0.33}CuSi_4O_{10}$ and undoped $Ba_{0.33}Sr_{0.33}Ca_{0.33}CuSi_4O_{10}$. c) 2D excitation-emission PL map of different Mg-doped copper tetrasilicates.

a $M_S = Ba_{0.33}Sr_{0.33}Mg_{0.33}$

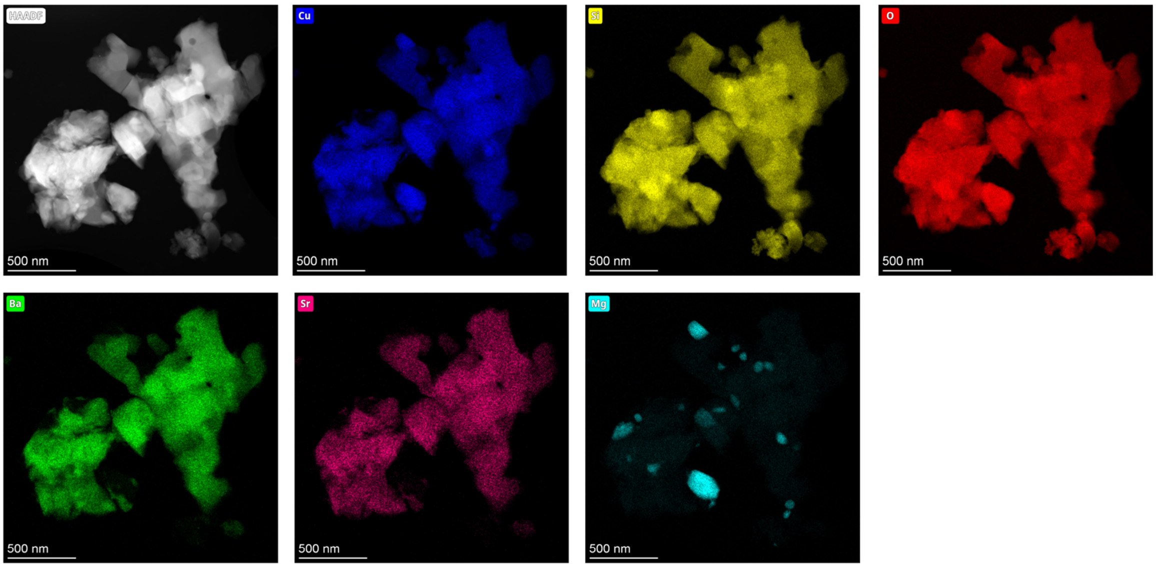

b

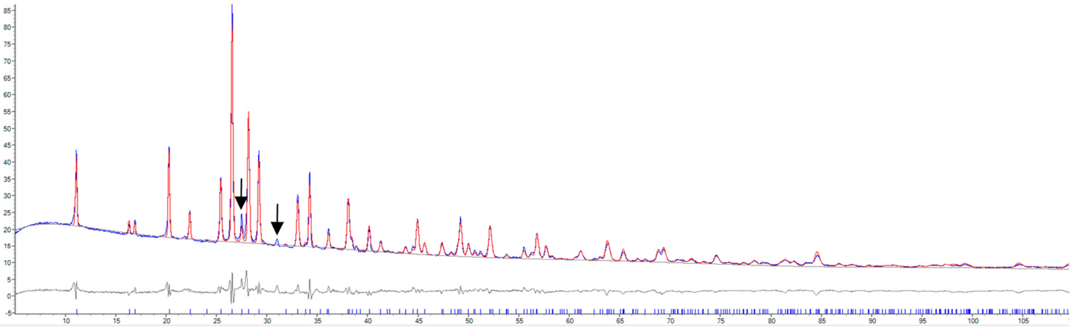

**$M_S = Ba_{0.33}Sr_{0.33}Mg_{0.33}$ (24h@1000°C)**	**phase 1 = $Ba_{0.5}Sr_{0.5}CuSi_4O_{10}$**
space group	P 4/n c c; #130
lattice parameters (Å)	a =7.397<br>c =15.880
Rwp = 3.01 %	

Supplementary Figure S25: Characterization of Mg-doped $Ba_{0.5}Sr_{0.5}CuSi_4O_{10}$. a) STEM analysis of nanosheets shown as HAADF image and EDX-based elemental maps. Strong segregation of Mg-containing phases becomes visible on single NS level. b) Rietveld analyses reveal no significant shift in lattice parameters compared to undoped $Ba_{0.5}Sr_{0.5}CuSi_4O_{10}$. Black arrows indicate the presence of a minor, unidentified phase, most likely containing Mg.

a $M_S = Sr_{0.33}Ca_{0.33}Mg_{0.33}$

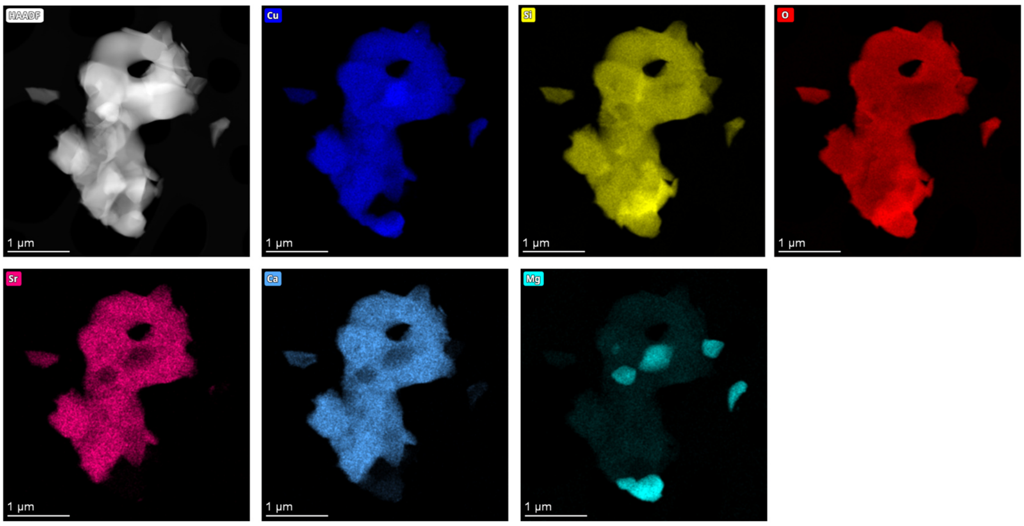

b

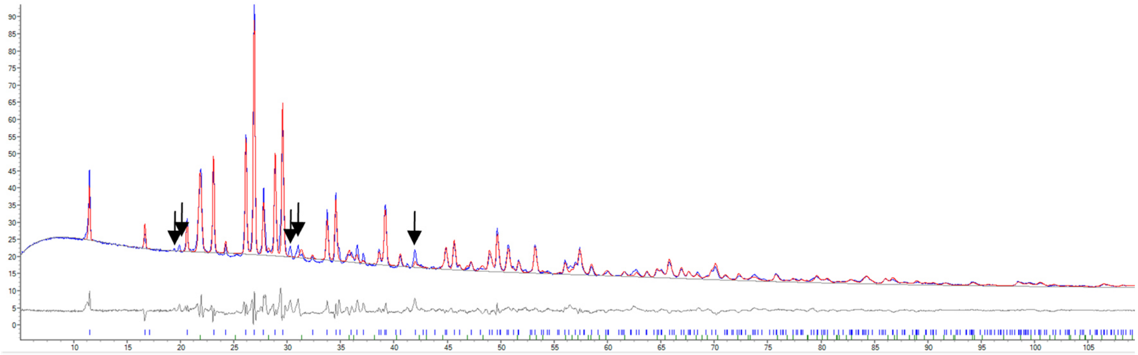

$M_S = Sr_{0.33}Ca_{0.33}Mg_{0.33}$ (24h@1000°C)	phase 1 = $Sr_{0.5}Ca_{0.5}CuSi_4O_{10}$	phase 2 = SiO_2
Space group	P 4/n c c; #130	P 4_1 2_1 2; #92
lattice parameters (Å)	a =7.333<br>c = 15.381	a = 5.010<br>c = 6.939
Rietveld quantification (%)	84.8	15.2
Rwp = 3.14 %		

Supplementary Figure S26: Characterization of Mg-doped $Sr_{0.5}Ca_{0.5}CuSi_4O_{10}$. a) STEM analysis of nanosheets shown as HAADF image and EDX-based elemental maps. Strong segregation of Mg-containing phases becomes visible on single NS level. b) Rietveld analyses reveal no significant shift in lattice parameters compared to undoped $Sr_{0.5}Ca_{0.5}CuSi_4O_{10}$, whereas in addition to it crystalline SIO_2 was detected. Black arrows indicate the presence of a minor, unidentified phase, most likely containing Mg.

a

M_S = $Ba_{0.25}Sr_{0.25}Ca_{0.25}Mg_{0.25}$

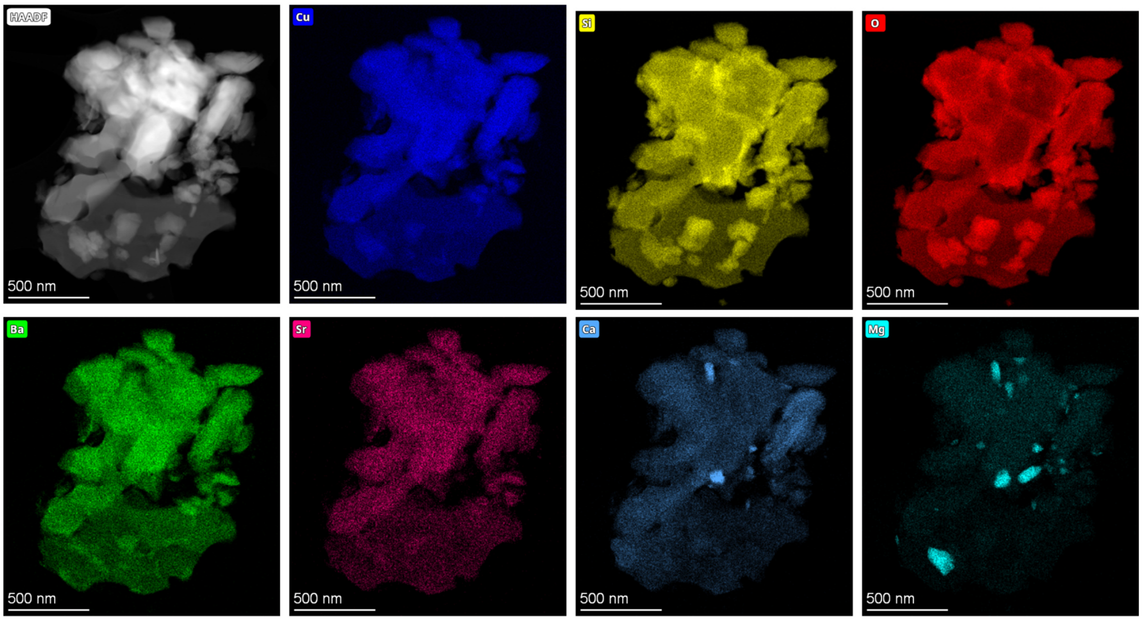

b

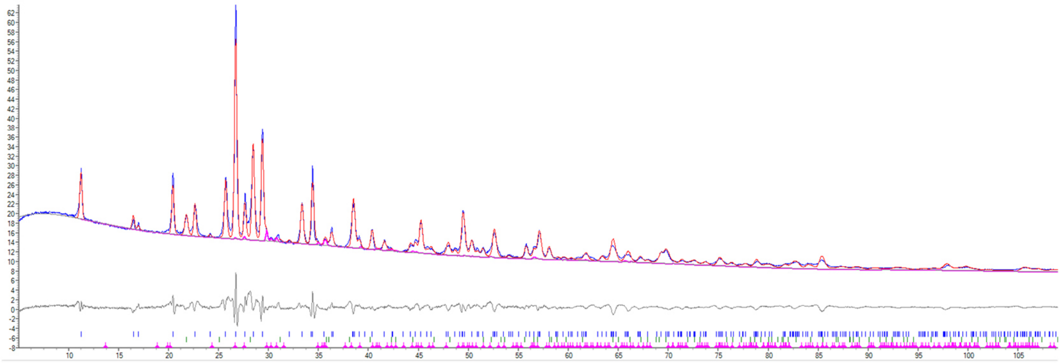

M_S = $Ba_{0.25}Sr_{0.25}Ca_{0.25}Mg_{0.25}$	**phase 1 =** $Ba_{0.33}Sr_{0.33}Ca_{0.33}CuSi_4O_{10}$	**phase 2 =** $CaMgSi_2O_6$	**phase 3 =** SiO_2
Space group	P 4/n c c; #130	C 2/c; #15	P 4_1 2_1 2; #92
Lattice parameters (Å)	a =7.365<br>c = 15.684	a = 9.775<br>b = 8.916<br>c = 5.252	a = 4.99<br>c = 6.95
Rietveld quantification (%)	89.3	5.3	4
Rwp = 3.13 %			

Supplementary Figure S27: Characterization of Mg-doped $Ba_{0.33}Sr_{0.33}Ca_{0.33}CuSi_4O_{10}$. a) STEM analysis of nanosheets shown as HAADF image and EDX-based elemental maps. Strong segregation of Mg-containing phases becomes visible on single NS level. b) Rietveld analyses reveal slight shift in lattice parameter *c* compared to undoped $Ba_{0.33}Sr_{0.33}Ca_{0.33}CuSi_4O_{10}$, whereas in addition to it crystalline diopside $CaMgSi_2O_6$ and a minor fraction of SiO_2 was detected.

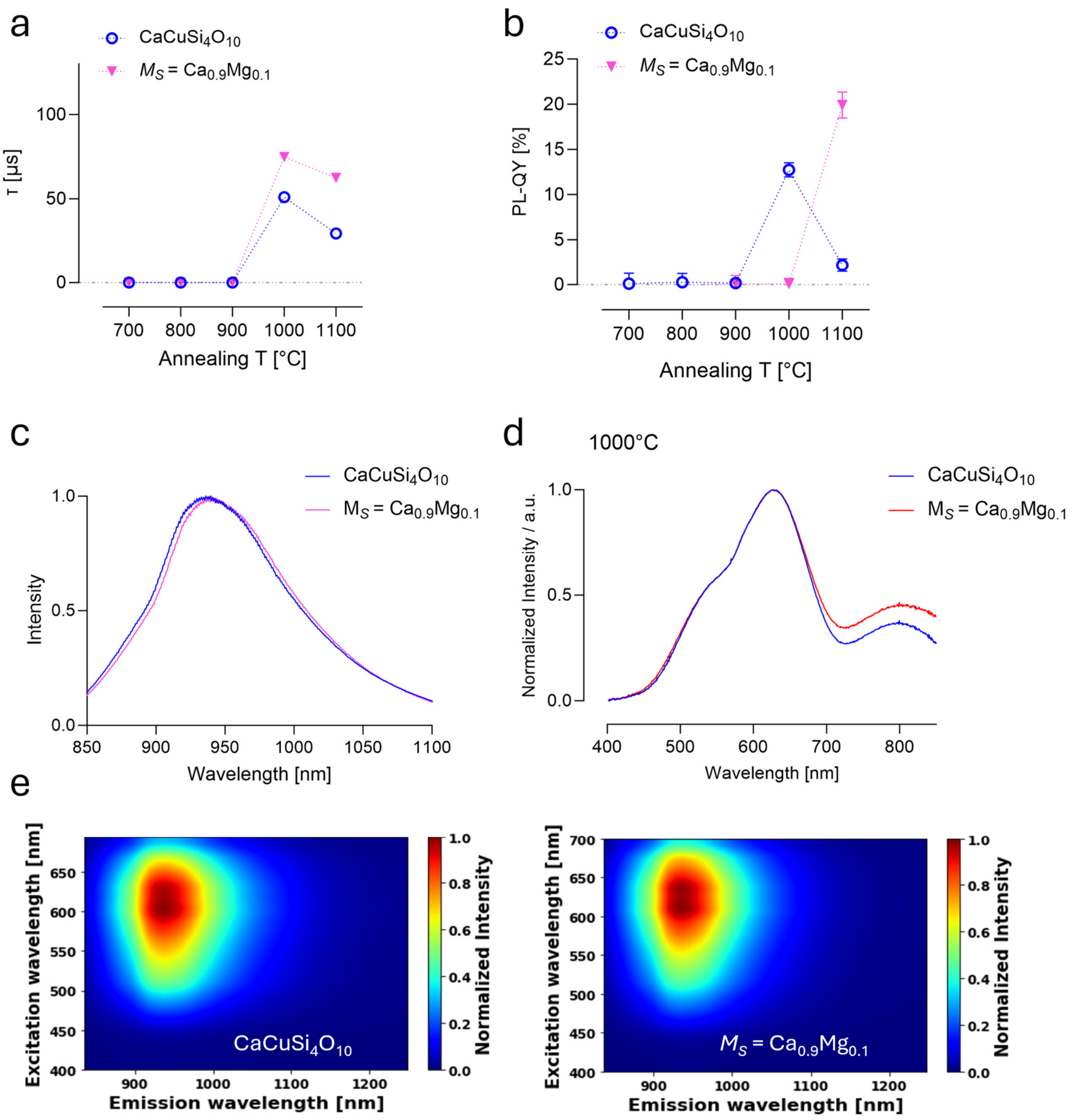

Supplementary Figure S28: Optical characterization of Mg-doped $CaCuSi_4O_{10}$. a) Modulation of fluorescence lifetime values and b) PL-QY with different annealing temperatures for 10 min each. c) PL emission and d) UV-Vis-NIR absorbance spectra of $M_S = Ca_{0.9}Mg_{0.1}$ and $CaCuSi_4O_{10}$ both annealed for 24 h at 1000 °C. Short annealing of $M_S = Ca_{0.9}Mg_{0.1}$ at 1000 °C is not sufficient to yield a copper tetrasilicate phase, whereas 10 min at 1100 °C or long annealing is. e) 2D excitation-emission PL map of $CaCuSi_4O_{10}$ compared to $M_S = Ca_{0.9}Mg_{0.1}$.

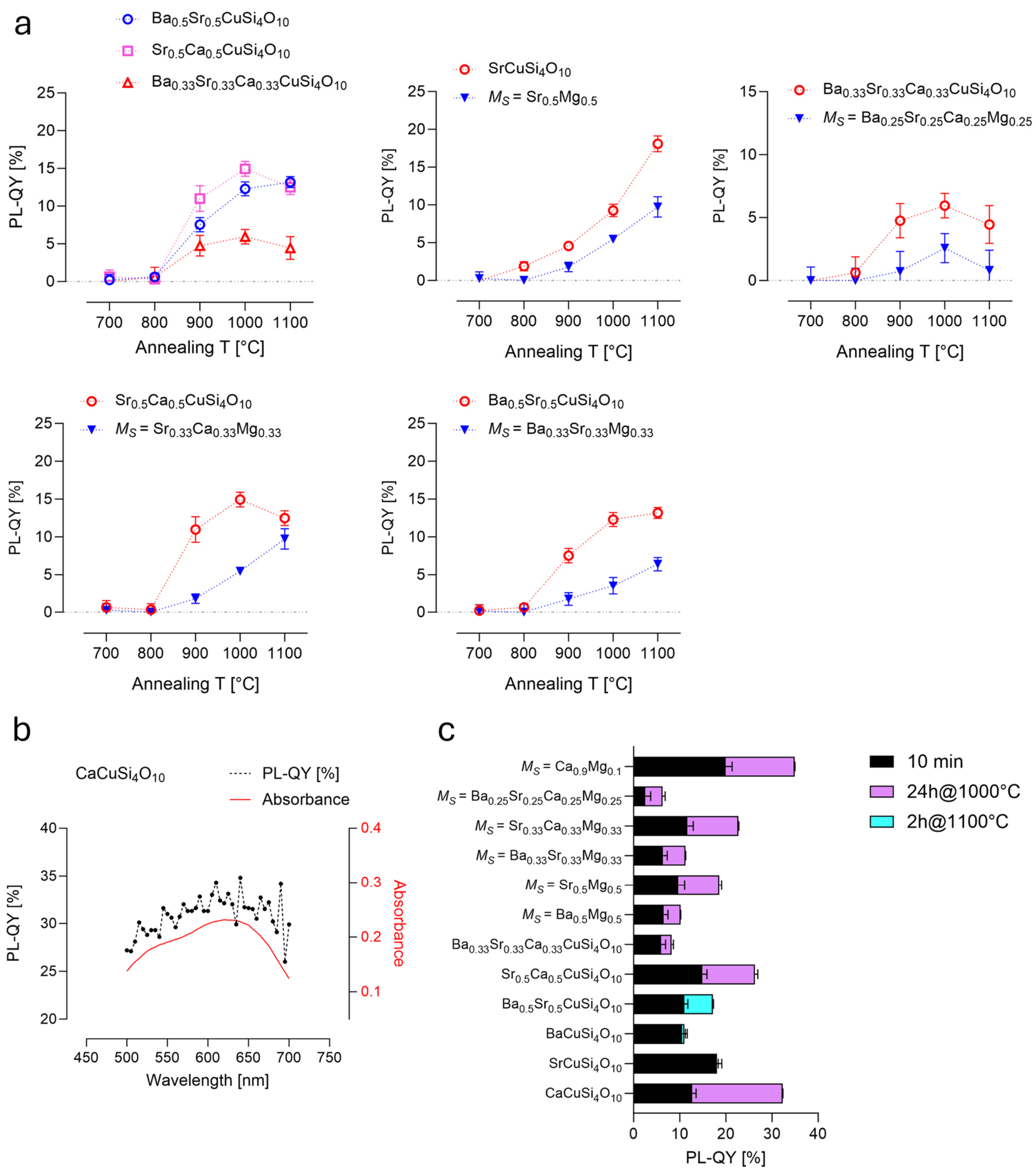

Supplementary Figure S29: Absolute photoluminescence quantum yield (PL-QY) engineering of multielement doped copper tetrasilicates. a) Overview of PL-QY dependency on the annealing temperature (for 10 min). b) Simultaneous absorbance and PL-QY measurement of $CaCuSi_4O_{10}$ indicates that about a third of the incoming light is absorbed (A ≈ 0.2), whereas max. 32% of the absorbed light is emitted again. Measurements performed with a Hamamatsu Quantaurus-QY® (C11347-12). c) Impact of annealing time on the tested materials. For 10 min annealing, the observed maximum was used, depending on the copper tetrasilicates either at 1000 °C or 1100 °C.

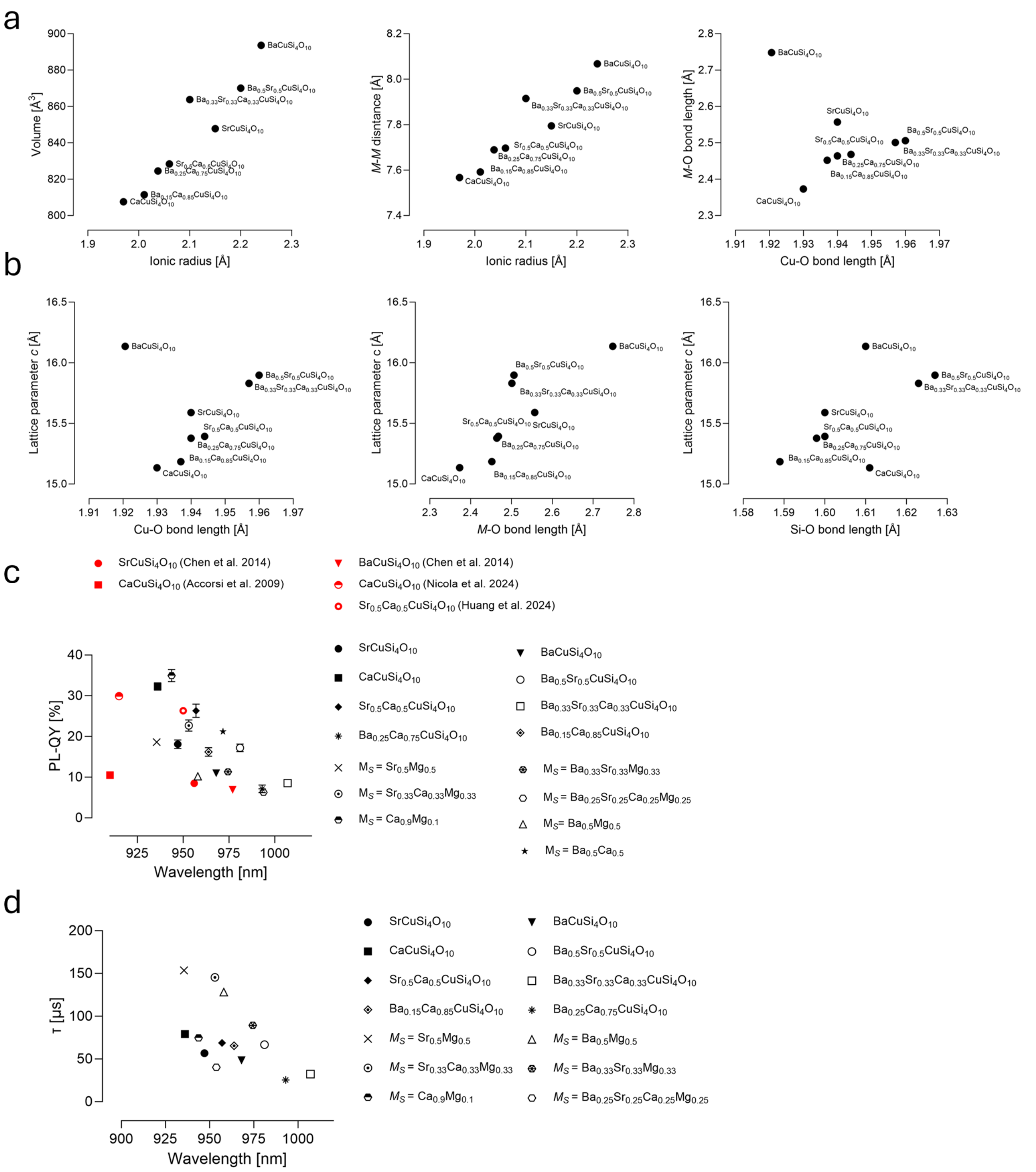

Supplementary Figure S30: Correlation of lattice parameters and optical properties of all synthesized 2D copper tetrasilicates variations with MCuSi$_4$O$_{10}$. a) Extension to Figure 5h; correlating the cell volume to the mean ionic radius, the *M*-*M* distance to the mean ionic radius and the mean *M*-O bond lengths to the Cu-O bond lengths. b) Correlation of lattice parameter *c* to mean *M*-O (3), Cu-O and mean Si-O bond lengths of the obtained CTS structures. c) Extension to Figure 5i,j: Optimized PL-QY compared to the fluorescence emission wavelengths, including reference values[1–5]. c) Fluorescence lifetime values compared to the fluorescence emission wavelengths.

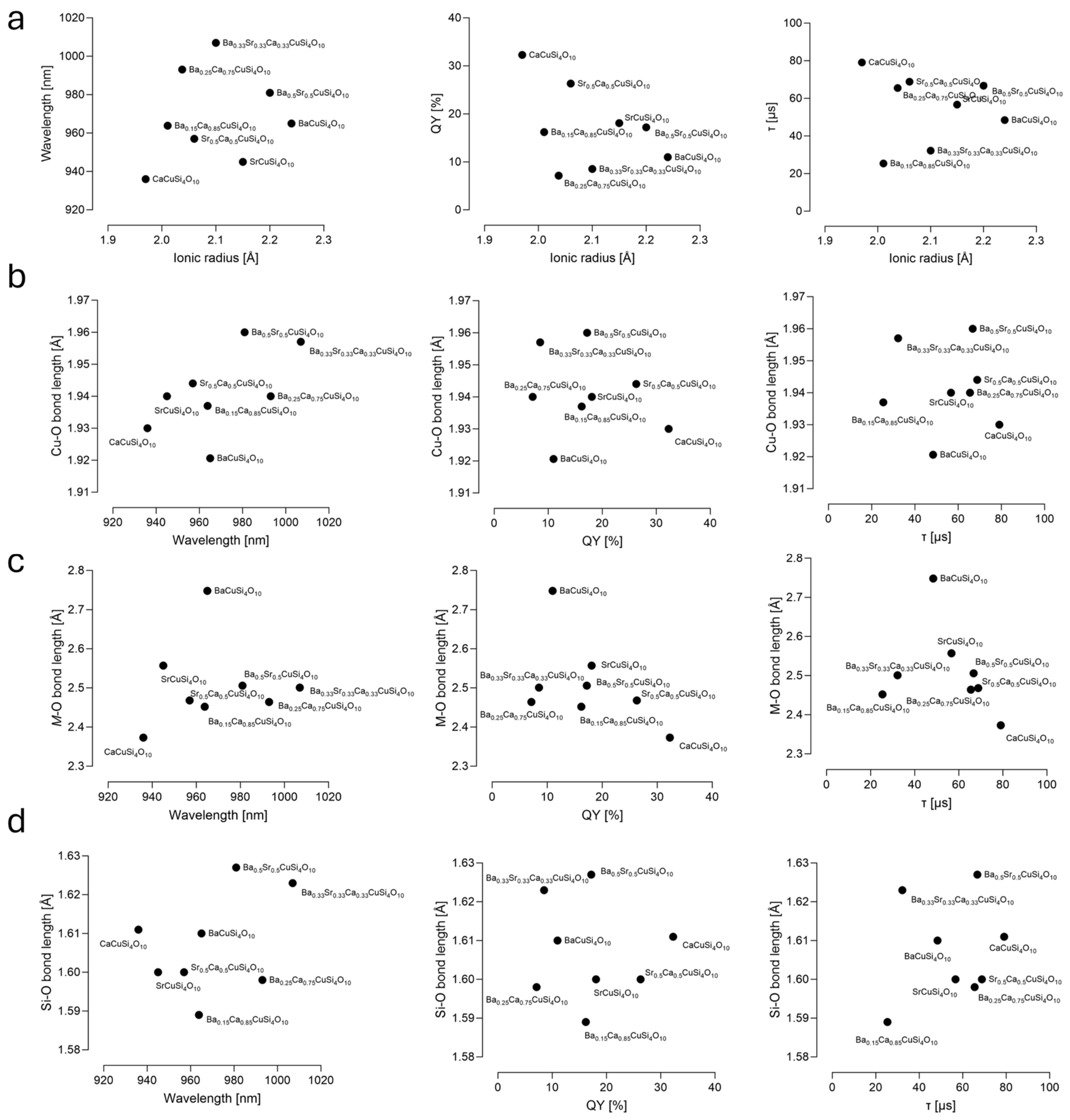

Supplementary Figure S31: Correlation between CTS lattice parameters and their optical properties, comparing the fluorescence emission maxima, QY and lifetimes against the mean ionic radius (a), the Cu-O bond lengths (b), the mean *M*-O (3) bond lengths (c) and the mean Si-O bond lengths (d).

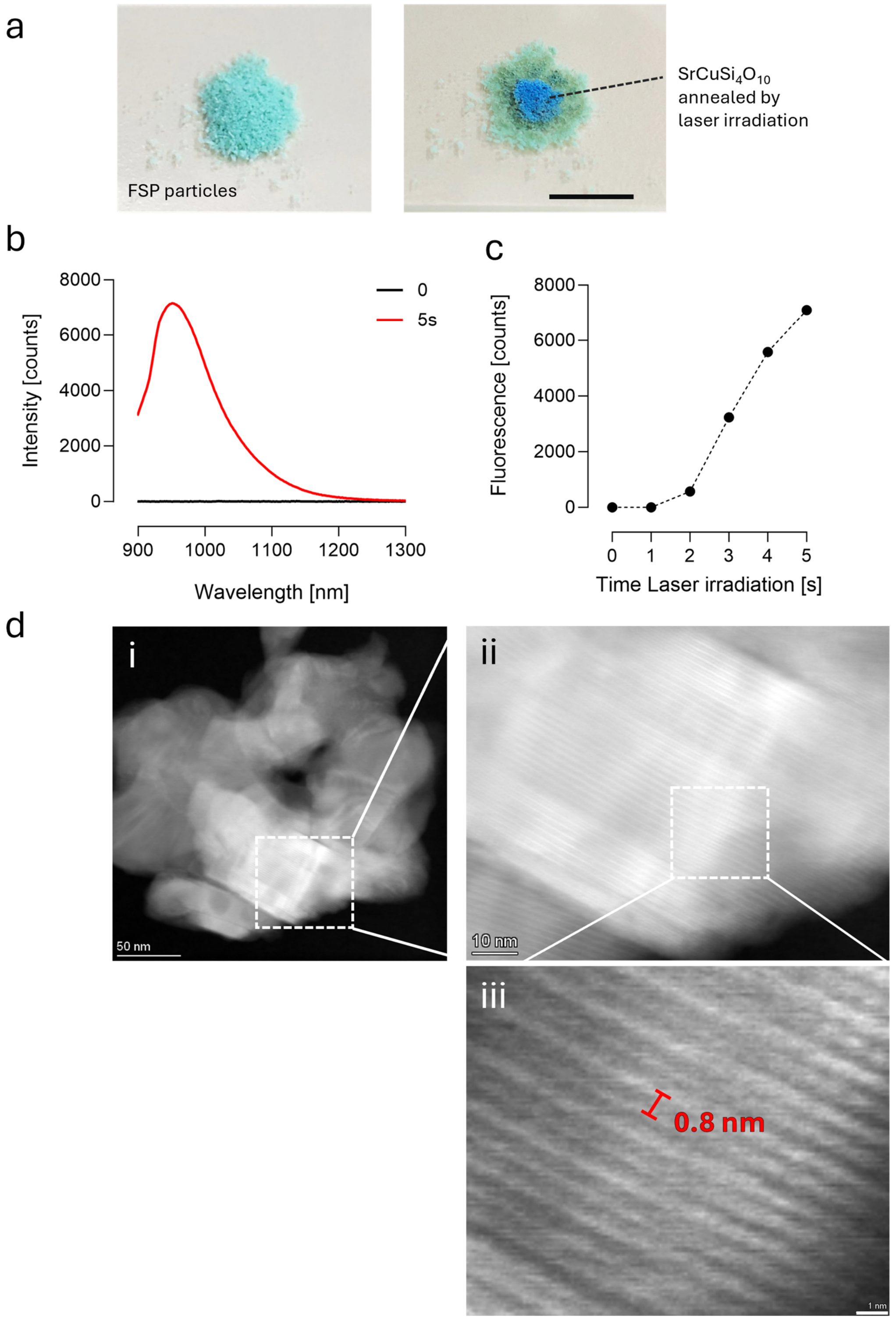

Supplementary Figure S32: Laser-induced annealing of $SrCuSi_4O_{10}$. a) Photographs of as-prepared FSP-particles before (left) and after (right) laser irradiation (scale bar = 0.5 cm). b) Fluorescence emission spectra of FSP-particles without (black) and after 5 seconds (red) of laser irradiation (808 nm, 15.3 W/cm^2). c) Fluorescence emission maxima plotted against the laser irradiation time. A clear signal is visible after 2-3 seconds of illumination. d) HR-STEM analysis of $SrCuSi_4O_{10}$ annealed through laser irradiation. i – HAADF image; ii – DF image (zoomed in region); iii – DF image (zoomed in region). A highly crystalline lattice with a spacing of approximately 0.8 nm is clearly observable.

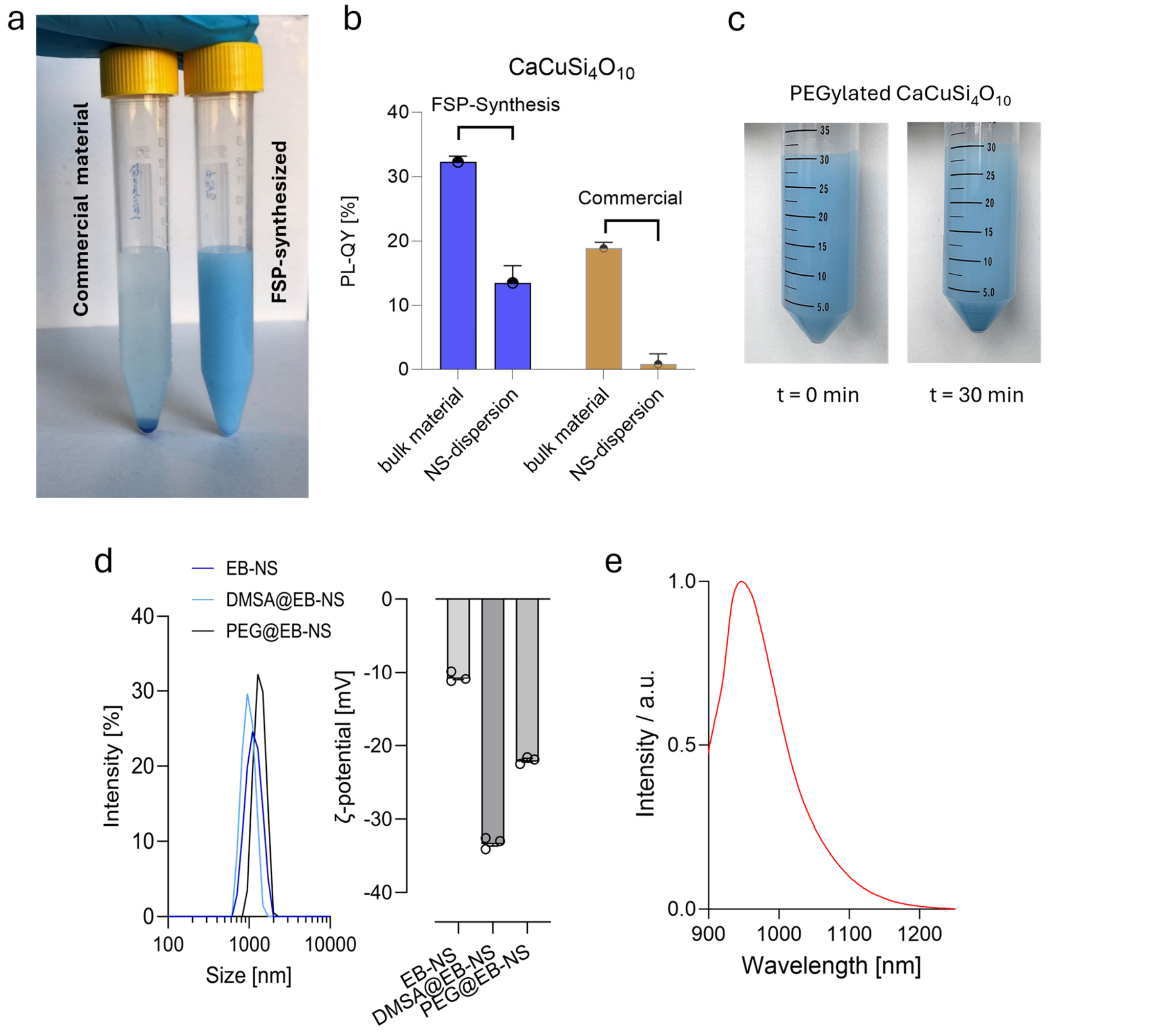

Supplementary Figure S33: Dispersion of $CaCuSi_4O_{10}$. a) Photograph of copper tetrasilicate material dispersed in water, using the same protocol as described in the Material and Method section (10 mg/mL; photograph taken about 10 min post dispersion). Left: commercial powder (Kremer Pigmente; <120 µ particle size). Right: FSP-derived material. b) PL-QY analysis of dispersed $CaCuSi_4O_{10}$ nanosheets (~13.5%) was performed in comparison with bulk material obtained from FSP synthesis followed by 24h annealing at 1000°C, as well as with commercial powder and its nanosheet dispersion derived through ball milling[6] (~0.8%). c) Photograph of PEGylated $CaCuSi_4O_{10}$ dispersed in water (3.3 mg/mL). After 30 minutes, only a small fraction of larger, sedimented particles is visible at the bottom of the tube, while the supernatant was collected as the nanosheet fraction for bioimaging tests. d) DLS-based particle size and Zeta-Potential evaluation for pristine $CaCuSi_4O_{10}$ NS and surface-modified versions via PEGylation and DMSA-coating. e) Photoluminescence emission spectrum of $CaCuSi_4O_{10}$ excited at 808 nm.

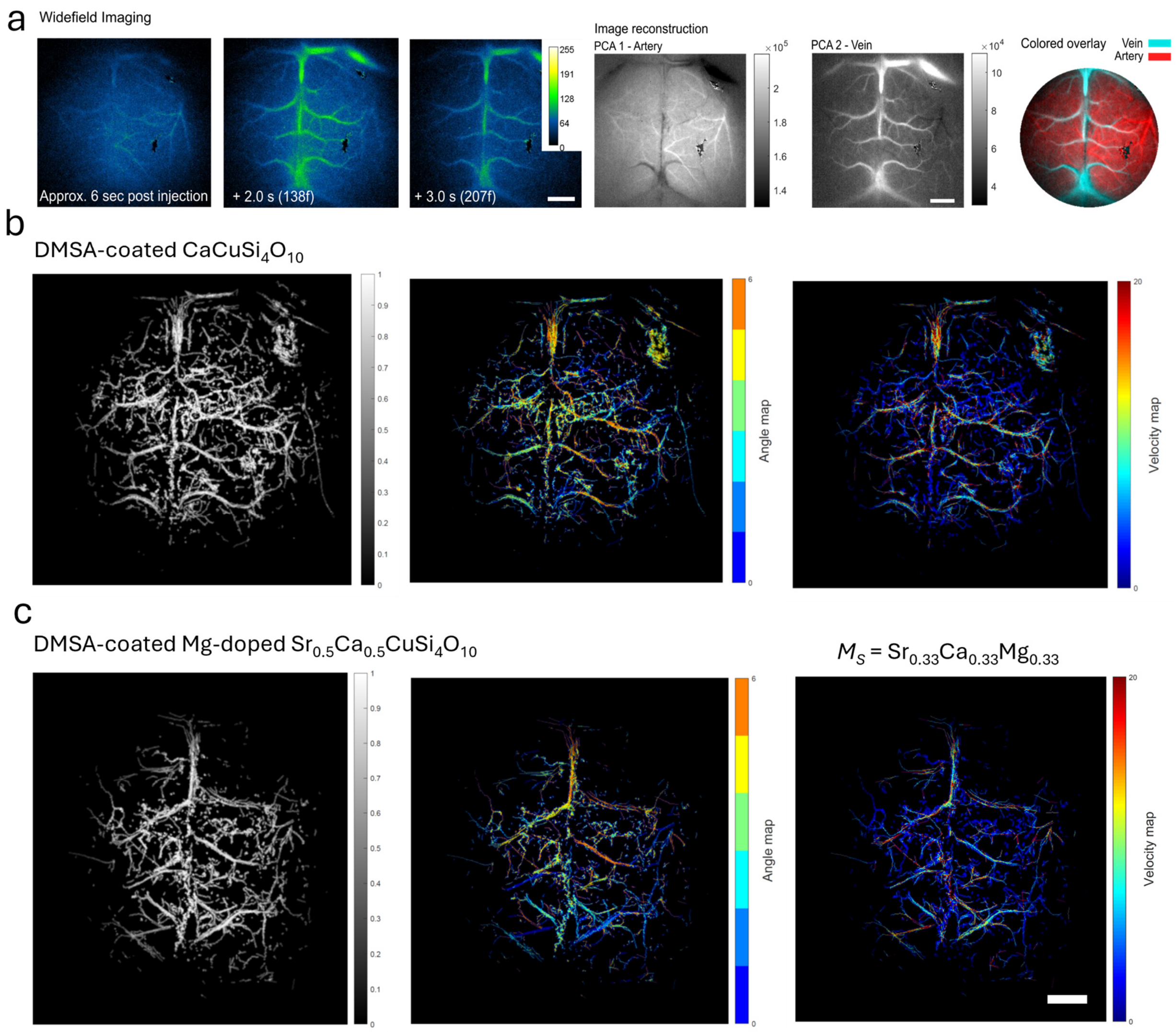

Supplementary Figure S34: In vivo bioimaging with DMSA-coated CTS-NS. a) Time-lapse widefield images post DMSA-stabilized $CaCuSi_4O_{10}$ NS injection. Veins and arteries could be differentiated based on principal component analysis (PCA) (scale bar = 1 mm). Note: The dark spots in the figure were caused by water reflection, which was removed during image processing. b) Diffuse optical localization imaging (DOLI) with structural (left), blood flow angle (middle) and velocity (right, mm/s) maps of cerebral vasculature rendered from continuous localization and tracking of circulating NS shown for DMSA-stabilized $CaCuSi_4O_{10}$ and in c) for DMSA-modified Mg-doped $Sr_{0.5}Ca_{0.5}CuSi_4O_{10}$ (M_S = $Sr_{0.33}Ca_{0.33}Mg_{0.33}$ annealed for 24 h at 1000 °C). Scale bar = 1 cm.

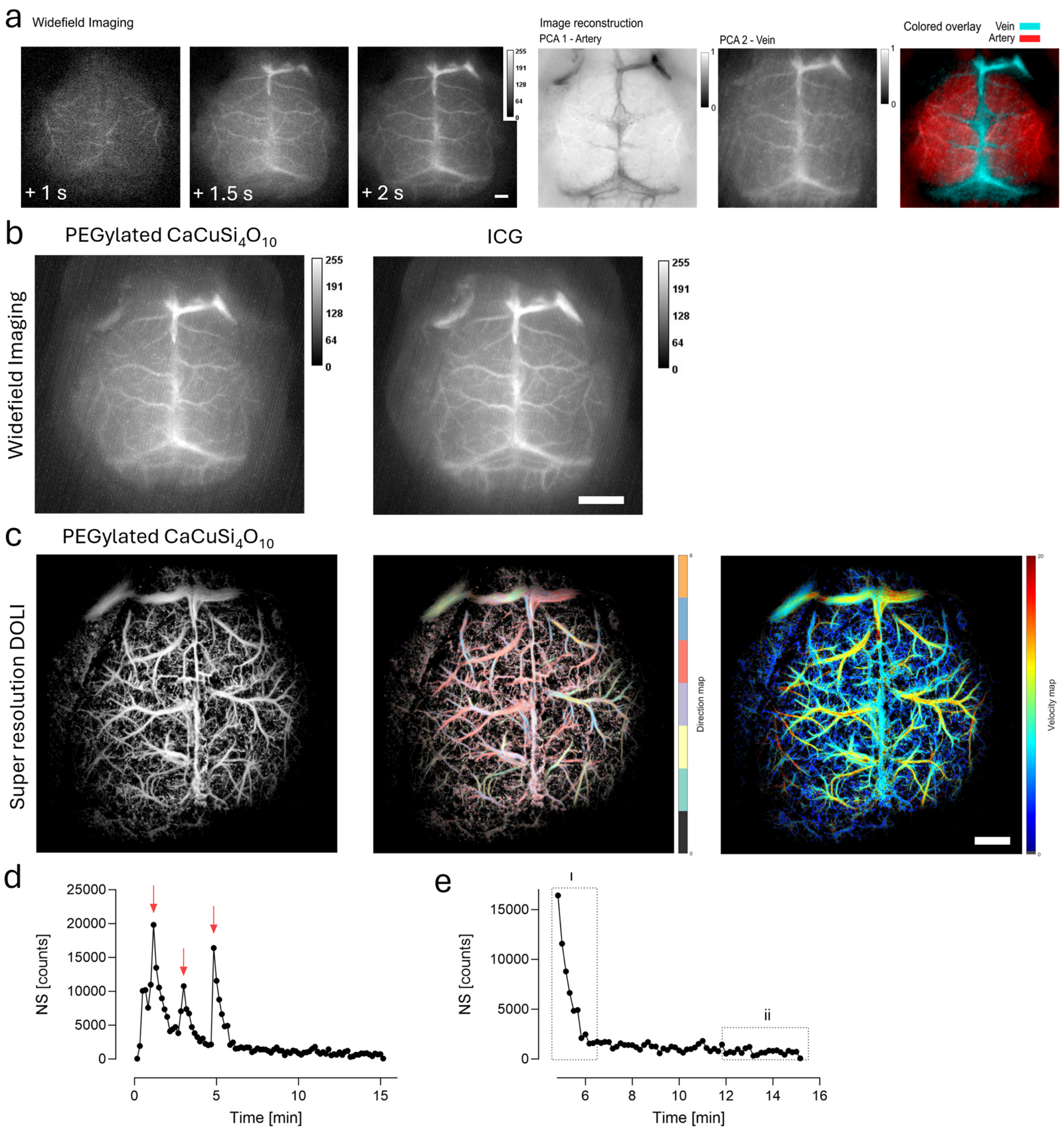

Supplementary Figure S35: In vivo bioimaging with PEGylated CTS-NS. a) Time-lapse widefield images post PEGylated $CaCuSi_4O_{10}$ NS injection. Veins and arteries could be differentiated based on principal component analysis (PCA) (scale bar = 1 mm). b) Widefield imaging performance of the NS compared to indocyanine green (ICG; 50 µL injection, 1 mg/mL) shows a similar, though less detailed, vascular image (scale bar = 2 mm). c) Diffuse optical localization imaging (DOLI) with structural (left), blood flow angle (middle) and velocity (right, mm/s) maps of cerebral vasculature rendered from continuous localization and tracking of circulating NS, representing the super resolution structural image from Figure 8c. d) Pharmacological evaluation of intravenously applied $CaCuSi_4O_{10}$ NS. Cumulative NS counts over 10 second intervals, visible on the whole cerebral region. Three injections (red arrows) of a total of 100 µL NS solution (10 mg/mL), leading to the detection of up to ~20.000 particles. e) Zoomed-in region for the third injection. Approximately 90% of the NS are cleared within the first 1.5 min after the injection (i), whereas about 5% of the particles (~800 individual particles) do circulate up to 10 min post injection (ii).

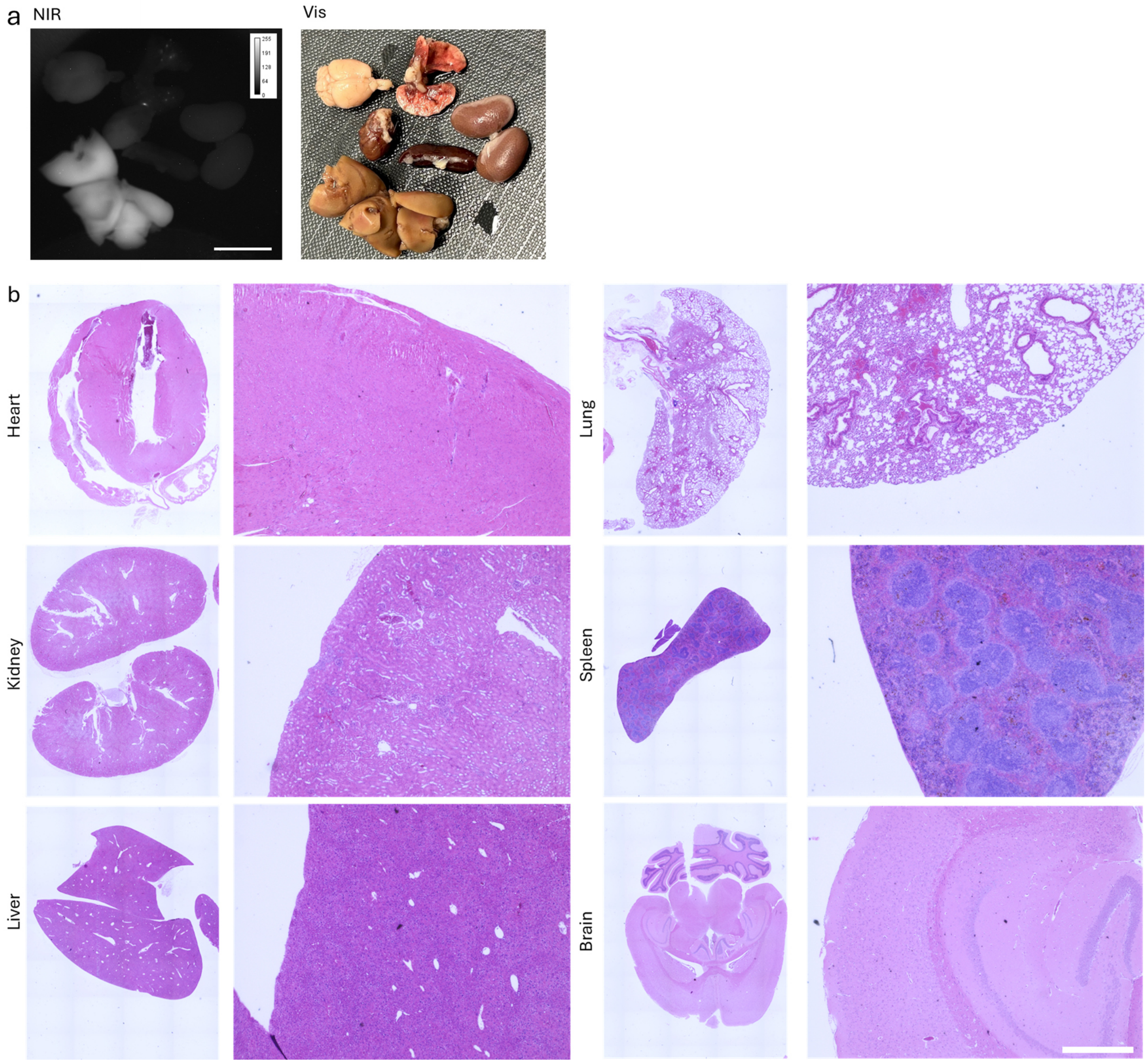

Supplementary Figure S36: Nanosheet biocompatibility assessment. a) Key mouse organs harvested after terminal in vivo bioimaging tests with PEGylated $CaCuSi_4O_{10}$ NS, imaged under NIR (900 nm LP; right) and visible light (left) conditions (scale bar = 2 cm). Most of the nanosheets appear to be retained in the liver. b) Histological analysis of the key organs (hematoxylin-blue, eosin-red (H&E) staining) obtained approximately 45 min after NS administration. No significant tissue damage was observed (scale bar = 650 μm). Organs were fixed in 4% formaldehyde solution (ROTI®Histofix, Carl Roth), then embedded, sectioned, and stained with hematoxylin and eosin (H&E) by Sophistolab AG (Muttenz, Switzerland). The slides were scanned using an EVOS M7000 microscope (Invitrogen) with a 4× objective.

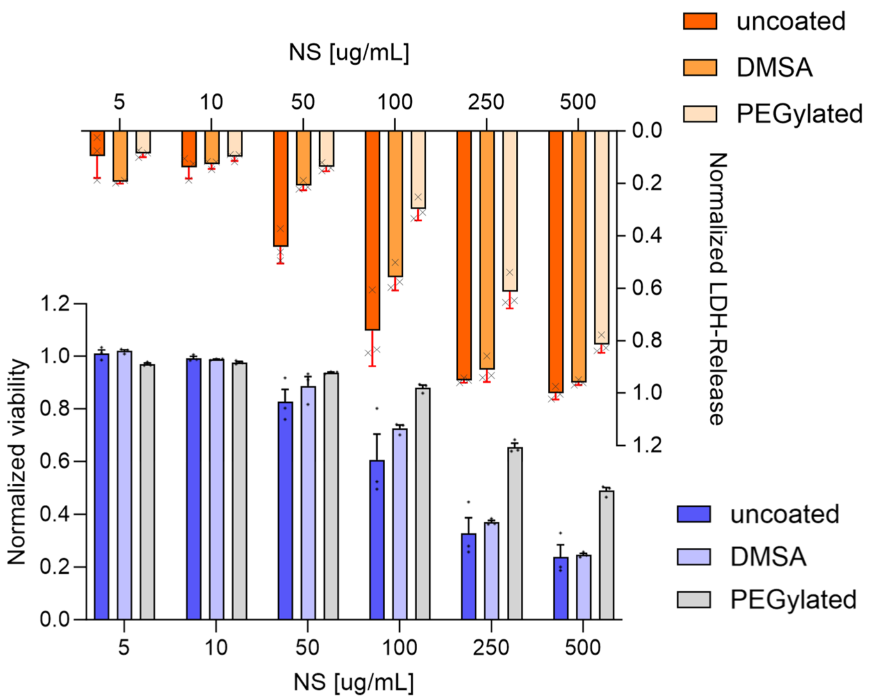

Supplementary Figure S37: Macrophage cell toxicity test for $CaCuSi_4O_{10}$ NS with different surface modifications, compared to uncoated / bare NS. PEGylation significantly improves the biocompatibility of the NS, enhancing the nanomaterial uptake.

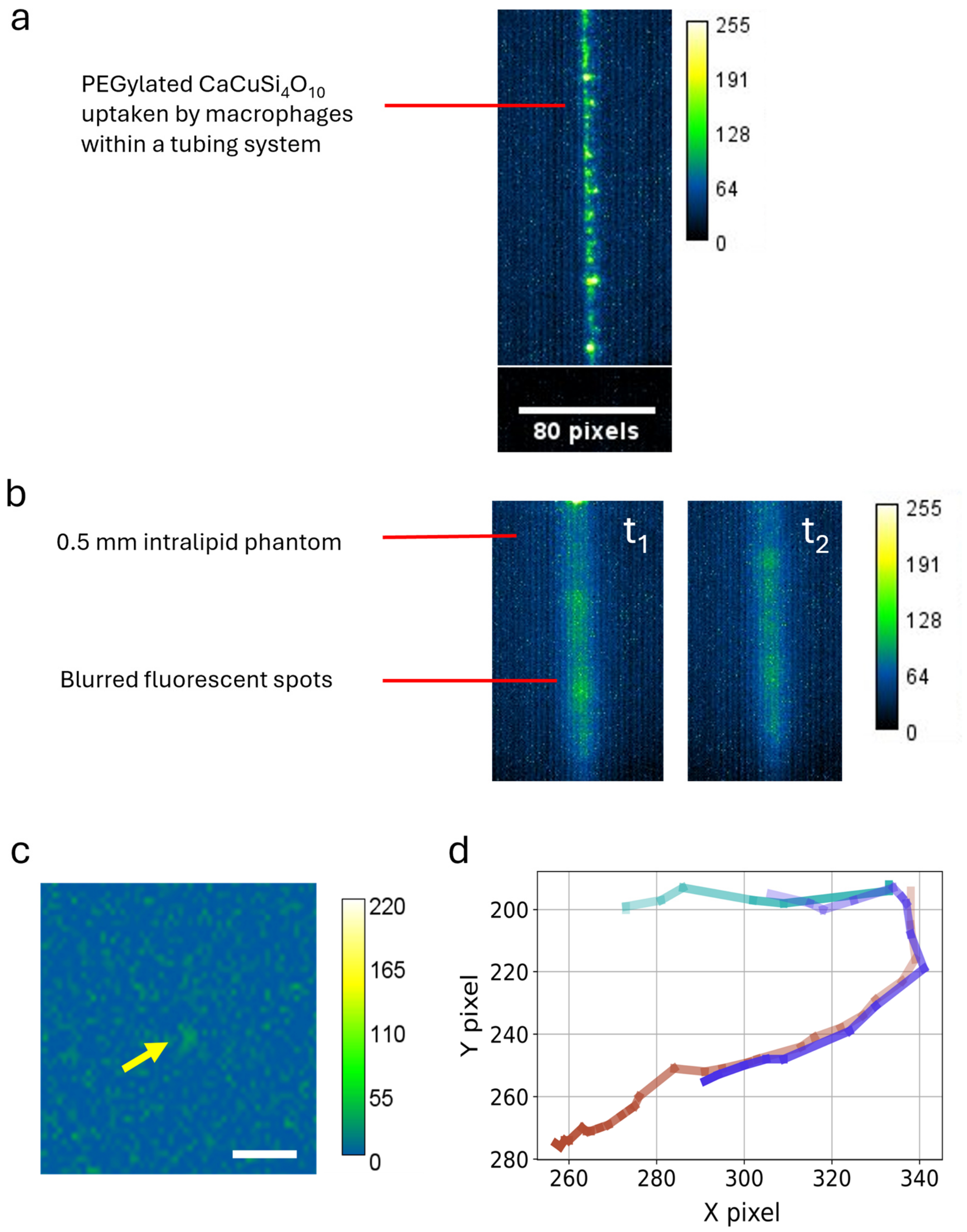

Supplementary Figure S38: High-frame-rate imaging of NS-labeled macrophages. a) Single-cell suspension of macrophages injected into the same microtubing used for NS imaging (Figure 6d). b) Fluorescence images of the same target, blurred after covering the tube with a tissue-mimicking intralipid (IL) phantom (1 pixel = 13.3 µm). c) Snapshot of a single, NS-loaded macrophage re-injected into the mouse via tail-vain injection and imaged on the whole cerebral scale (69 fps, scale bar = 173 µm). d) Trajectories of three individually tracked macrophages (1 pixel = 13.3 µm).

Table S2: Typical FSP-precursor solution to yield mono-alkaline earth metal copper tetrasilicates (total of 80 mL and 0.2 M metal ion concentration, optimized Ca and Cu concentrations). Cu - Borchers® Deca Copper 8 (8 wt% Cu); Si – Hexamethyldisiloxane.

**Sample**	***M***	**Cu**	**Si**	**1:1 (2-EHA:THF)**
$CaCuSi_4O_{10}$	20 mL Calcium naphthenate in mineral spirits (4 wt% Ca)	11.2 mL	6.8 mL	46 mL
$SrCuSi_4O_{10}$	3.29 g Strontium acetate dissolved in 2-EHA under reflux at 130 °C	11.2 mL	6.8 mL	62 mL
$BaCuSi_4O_{10}$	4.09 g Barium acetate dissolved in 2-EHA under reflux at 130 °C	11.2 mL	6.8 mL	62 mL

Table S3: List of mineral names, chemical formula and ICSD code for the identified phases.

**Mineral name**	**Chemical formula**	**Crystal system**	**Space group (#)**	**ICSD code**
Cuprorivaite	$CaCuSi_4O_{10}$	tetragonal	P4/ncc (130)	72612
Wesselsite	$Sr_{1-x}Ba_xCuSi_4O_{10}$	tetragonal	P4/ncc (130)	168533
Effenbergerite	$BaCuSi_4O_{10}$	tetragonal	P4/ncc (130)	75970
Diopside	$CaMgSi_2O_6$	monoclinic	C12/c (15)	10222
Cristobalite	SiO_2	tetragonal	$P4_12_12$ (92)	72612
Sanbornite	$BaSi_2O_5$	orthorhombic	Pcmn (62)	15486

References:

(1) Chen, Y.; Zhang, Y.; Feng, S. Hydrothermal Synthesis and Properties of Pigments Chinese Purple BaCuSi2O6 and Dark Blue BaCu2Si 2O7. *Dye. Pigment.* **2014**, *105*, 167–173. https://doi.org/10.1016/j.dyepig.2014.01.017.

(2) Chen, Y.; Shang, M.; Wu, X.; Feng, S. Hydrothermal Synthesis, Hierarchical Structures and Properties of Blue Pigments SrCuSi4O10 and BaCuSi4O10. *CrystEngComm* **2014**, *16* (24), 5418–5423. https://doi.org/10.1039/c3ce42394h.

(3) Nicola, M.; Garino, C.; Mittman, S.; Priola, E.; Palin, L.; Ghirardello, M.; Damagatla, V.; Nevin, A.; Masic, A.; Comelli, D.; Gobetto, R. Increased NIR Photoluminescence of Egyptian Blue via Matrix Effect Optimization. *Mater. Chem. Phys.* **2024**, *313* (December 2023), 128710. https://doi.org/10.1016/j.matchemphys.2023.128710.

(4) Huang, W.; Zhang, J.; Fan, J.; Chen, P.; Zhou, L.; Zhang, X. From Ancient Blue Pigment to Unconventional NIR Phosphor: A Thermal-Stable Near-Infrared I/II Broadband Emission from Ca1-XSrxCuSi4O10 Solid Solution. *Inorg. Chem.* **2024**, *63* (1), 812–823. https://doi.org/10.1021/acs.inorgchem.3c03811.

(5) Accorsi, G.; Verri, G.; Bolognesi, M.; Armaroli, N.; Clementi, C.; Miliani, C.; Romani, A. The Exceptional Near-Infrared Luminescence Properties of Cuprorivaite (Egyptian Blue). *Chem. Commun.* **2009**, No. 23, 3392–3394. https://doi.org/10.1039/b902563d.

(6) Hill, B.; Abraham, S.; Akhtar, A.; Selvaggio, G.; Tschulik, K.; Kruss, S. Surfactant Assisted Exfoliation of near Infrared Fluorescent Silicate Nanosheets. *RSC Adv.* **2023**, *13* (30), 20916–20925. https://doi.org/10.1039/d3ra04083f.
